# Practical application of good participatory practices for trials of emerging pathogens: Developing materials for use in ACTIV-3, -3b, and ACTIV-associated COVID-19 trials

**DOI:** 10.1017/cts.2024.485

**Published:** 2024-10-15

**Authors:** Paola del Carmen Guerra-de-Blas, Rubria Marines-Price, Olga Milman, Danae Deal, Jonathan Marchand, Jessica Linton, Sue Meger, John Rule, Thomas L. Holland, Jonathan Kitonsa, Yvette Delph, for the INSIGHT OTAC, ACTIV-3 TICO, ACTIV-3 substudy VATICO, and ACTIV-3b TESICO Study Groups

**Affiliations:** 1 The Mexican Emerging Infectious Diseases Clinical Research Network (LaRed), Mexico City, Mexico; 2 Office of Advanced Practice Providers, University of Texas Southwestern Medical Center, Dallas, TX, USA; 3 The Bronx Veterans Medical Research Foundation Inc., James J. Peters Veteran Affairs Medical Center, Bronx, NY, USA; 4 Clinical Monitoring Research Program Directorate, Frederick National Laboratory for Cancer Research, Frederick, MD, USA; 5 Division of Biostatistics and Health Data Science, School of Public Health, University of Minnesota, Minneapolis, MN, USA; 6 National Association of People with HIV, Sydney, NSW, Australia; 7 Division of Infectious Diseases, Duke University, Durham, NC, USA; 8 MRC/UVRI & LSHTM Uganda Research Unit, Entebbe, Uganda; 9 Axle Informatics, North Bethesda, MD, USA

**Keywords:** Good Participatory Practice, stakeholder engagement, participant-facing materials, COVID-19 trials, informed consent process, recruitment, clinical trial implementation

## Abstract

The emergence of the COVID-19 pandemic required an immediate global clinical research response. The ACTIV (Accelerating COVID-19 Therapeutic Interventions and Vaccines)-3 trials and the ACTIV-associated Outpatient Treatment with Anti-Coronavirus Immunoglobulin trial used Good Participatory Practices (GPP) to develop materials for study implementation from a global network perspective. GPP guidelines offer a framework for engaging stakeholders throughout the research process. This paper provides an overview of the materials developed and their applicability in various settings, reports results from a survey of study site personnel on the materials’ usefulness, summarizes important lessons learned, and serves as a reference for networks eager to apply GPP. Survey results showed that flipbooks and overview videos were highly ranked. Stakeholder input was valuable in developing easily understandable participant-facing materials with culturally appropriate images. Materials should be available to submit with the initial protocol submissions to ethics committees, and in formats that accommodate a wide range of institutional resources, policies, and infection-control practices. This article emphasizes the importance of GPP, including stakeholder consultation, in developing materials that support clinical research and address language, cultural, and sociopolitical barriers during a pandemic. The findings will be used to optimize efforts and resource allocation for new and ongoing studies.

## Introduction

Conducting multi-country trials requires considering differences in culture, infrastructure, research experience, health policies, resources, and community dynamics. Stakeholders are those who can impact or who are impacted by the proposed research. Defining and involving stakeholders for the particular study enhances understanding across differences, promotes acceptance, and improves the quality of research findings [1,2,3]. The origins of Good Participatory Practices (GPP) trace back to the late 1990s and early 2000s when the human immunodeficiency virus (HIV)/acquired immunodeficiency syndrome pandemic prompted heightened awareness of the importance of community involvement in research [4]. In 2007, the first GPP guidelines were published to provide a structure for creating successful stakeholder engagement for HIV trials [5,6]. GPP offers a platform for integrating regional and international viewpoints to meet stakeholders' interests more effectively, as well as to enhance participant welfare, health equity, and overall research quality [5,6].

In 2012, GPP guidelines were published for tuberculosis drug trials [7]. The Ebola outbreak of 2014–2016 prompted the formulation of GPP guidelines for trials of (re-) emerging pathogens (GPP-EP) [8]. The GPP-EP highlights that involving stakeholders in clinical trials could strengthen epidemic response. GPP offers principles and processes for meaningful engagement with stakeholders to integrate local, regional, and international viewpoints and concerns into the design and conduct of clinical trials [9,10]. The principles that guide this engagement reflect values that are essential to initiating and sustaining partnerships: respect, fairness, integrity, transparency, accountability, and autonomy. The GPP-EP delineates optimal practice in nine different areas throughout the research life cycle: 1. Formative research activities; 2. Stakeholder engagement plan; 3. Communications and issues management plan; 4. Protocol development; 5. Informed consent process; 6. Standard of prevention and care; 7. Policies on trial-related harms; 8. Trial accrual, follow-up, and exit; and 9. Trial closure, results dissemination, and post-trial access to trial products or procedures [8].

COVID-19 required an immediate global response to rapidly implement clinical trials worldwide to identify effective medical countermeasures. This was met in part by international networks. However, the practical application of GPP-EP to develop materials for study implementation within the context of COVID-19 posed several challenges, including limitations on in-person interactions, time constraints for GPP planning and stakeholder engagement, and healthcare worker burnout. Not all aspects of the GPP-EP guidelines were utilized due to the rapid growth of the unanticipated COVID-19 pandemic and the relatively late formation of a stakeholder team.

The aim of this manuscript is to describe the GPP Stakeholder Team’s practical application of GPP-EP guidelines for developing and producing materials for study implementation in several clinical trials from a global network perspective within the context of the COVID-19 pandemic, to report the results from a survey of study site personnel on materials’ usefulness, and to summarize the main lessons learned.

## ACTIV COVID-19 therapeutic clinical trials

At the outset of the COVID-19 pandemic, there was no single infrastructure capable of implementing the global clinical research response required [11]. The United States (US) National Institutes of Health (NIH) unveiled the Accelerating COVID-19 Therapeutic Interventions and Vaccines (ACTIV) public-private partnership on April 17, 2020, with the goal of prioritizing and accelerating the development of the most promising therapeutics and vaccines [11,12,13]. Coordinated by the Foundation for the National Institutes of Health, ACTIV partnered the NIH with other agencies in the US Department of Health and Human Services (DHHS); other US government agencies including the Department of Defense and Department of Veterans Affairs; the Countermeasures Acceleration Group (formerly known as Operation Warp Speed (OWS)); the European Medicines Agency; and representatives from academia, nonprofit and philanthropic organizations, and numerous biopharmaceutical companies [14]. ACTIV tested 37 therapeutic agents using 11 master protocols that were designed and launched during the pandemic. In addition, there were several ACTIV-associated clinical trials: four Adaptive COVID-19 Treatment Trials of remdesivir, three trials of convalescent plasma, and two trials of anti-coronavirus hyperimmune intravenous immunoglobulin (hIVIG) [15]. The ACTIV Strategies and Treatments for Respiratory Infections and Viral Emergencies (STRIVE) master protocol was launched at the end of 2022 – materials produced for STRIVE were not evaluated for this manuscript. The initial two STRIVE trials focus on COVID-19, but the master protocol has the potential to study additional pathogens [16,17].

The International Network for Strategic Initiatives in Global HIV Trials (INSIGHT) was selected by the ACTIV Therapeutic Clinical Working Group and funded by the US government to work with three other networks (the Cardiothoracic Surgical Trials Network (CTSN), the Prevention and Early Treatment of Acute Lung Injury (PETAL) Network, and the Veterans Affairs Research Network (VA)) to develop the inpatient antiviral ACTIV master protocol, ACTIV-3. The networks all had extensive experience managing clinical trials and collectively worked with more than 300 sites across Africa, Asia, Australia, Europe, and North and South America [18].

INSIGHT had experience conducting HIV treatment trials since 2006, and more recently in other infectious diseases including influenza. Both CTSN and PETAL were funded by the NIH’s National Heart, Lung, and Blood Institute and had expertise in developing and conducting clinical trials in the intensive care unit (ICU) for participants experiencing heart and lung diseases. The VA network, funded by the US Department of Veterans Affairs, has a large, diverse patient population in many underrepresented areas. This collaboration of large, experienced networks was thought to be essential for recruiting a demographically and geographically diverse study population, thus increasing the applicability of beneficial treatments. Furthermore, the collaboration enabled dissemination of the most effective and efficient approaches for operations and trial conduct from each network across the combined network and allowed for cross-study use of templates and study materials.

**Figure 1.**
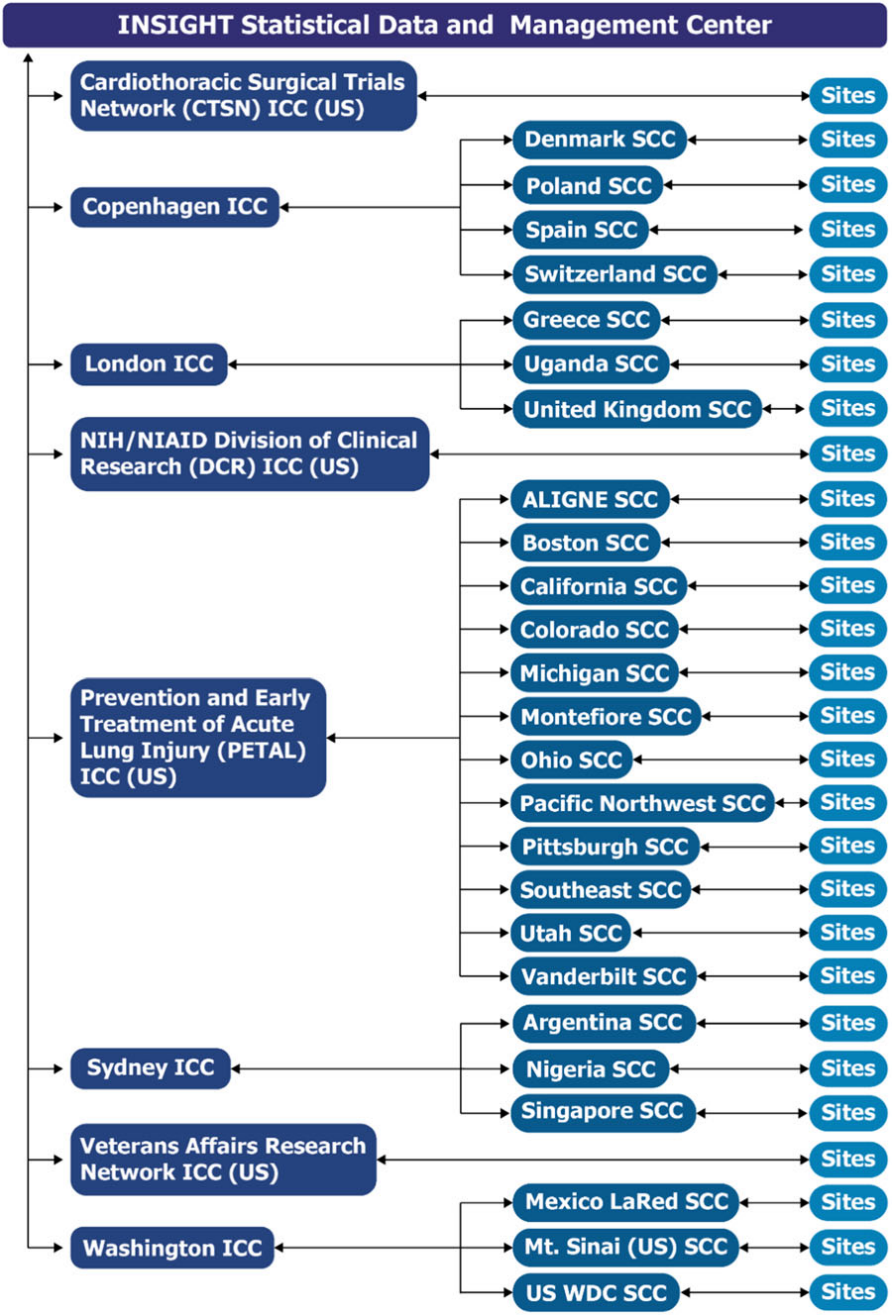
ACTIV -3, -3 substudy, -3b and outpatient treatment with anti-coronavirus immunoglobulin (OTAC) trials infrastructure and communication pathways. The infrastructure established for developing and implementing ACTIV (Accelerating COVID-19 Therapeutic Interventions and Vaccines)-3, -3 substudy, -3b, and ACTIV-associated COVID-19 clinical trials consisted of a central International Network for Strategic Initiatives in Global HIV Trials (INSIGHT) Statistical and Data Management Center (SDMC) at the University of Minnesota that oversaw various international coordinating centers (ICCs) that managed site coordinating centers (SCCs) and sites in various countries. Sites listed in the figure are those which enrolled in at least one of the studies through July 31, 2022. Abbreviations: ALIGNE = Acute Lung Injury Group New England; CTSN = Cardiothoracic Surgical Trials Network; DCR = Division of Clinical Research; LaRed = Mexican Emerging Infectious Disease Clinical Research Network; NIH/NIAID = National Institutes of Health/National Institute of Allergy and Infectious Diseases; PETAL = Prevention and Early Treatment of Acute Lung Injury; SCC = Site Coordinating Centers; US = United States of America; WDC = Washington, District of Columbia.

The Division of Clinical Research (DCR), of the National Institute of Allergy and Infectious Diseases (NIAID) within the NIH, funded ACTIV-3, ACTIV-3b, and other ACTIV-associated trials studying anti-coronavirus hIVIG [18,19]. DCR also funded the STRIVE trials developed near the end of the COVID-19 pandemic, to which lessons learned in implementing the ACTIV and ACTIV-associated trials were applied. The infrastructure established for developing and implementing these trials consisted of the central INSIGHT Statistical and Data Management Center (SDMC) at the University of Minnesota that oversaw various INSIGHT international coordinating centers (ICCs) that managed site coordinating centers (SCCs) and sites in various countries (Fig. 1). The CTSN, PETAL, and VA networks became ICCs. As a temporary measure to bolster the collaborative network infrastructure and increase enrollment, DCR formed an ICC and provided other network operational support, including for GPP.

A total of 134 sites in 12 countries enrolled 3,530 participants in four trials (Table 1) through July 31, 2022.

**Table 1.** Summary of DCR-funded COVID-19 trials. The table provides a brief description, enrollment information through July 31, 2022, ClinicalTrials.gov National Clinical Trial (NCT) number, and study products investigated for each of the four COVID-19 trials funded by the Division of Clinical Research (DCR), National Institute of Allergy and Infectious Diseases, National Institutes of Health, United States government for which materials were provided for study implementation

Trial	Brief Description	Enrollment Period and Number of Participants	Location of Sites and Enrollment Through July 31, 2022	Study Product(s) Investigated
OTAC (Outpatient Treatment with Anti-Coronavirus Immunoglobulin) NCT04910269	International multicenter, randomized, double-blind, placebo-controlled trial evaluating the safety and efficacy of anti-coronavirus hyperimmune intravenous immunoglobulin (hIVIG) for the treatment of adult outpatients, over 55 years of age or over 18 years of age with immune deficiency, in early stages of COVID-19	August 2021 and ongoing Planned enrollment: 820 participants. 239 enrolled through July 31, 2022	US: 80 Greece: 64 Denmark: 44 Mexico: 35 Spain: 10 Argentina: 6	Anti-coronavirus hyperimmune intravenous immunoglobulin (hIVIG)
ACTIV-3/TICO (Therapeutics for Inpatients with COVID-19) NCT04501978	Adaptive platform trial for investigating the safety and efficacy of new drugs for treating COVID-19 in people who have been hospitalized with COVID-19	August 2020 to December 2021 2,752 participants	US: 2,166 Spain: 150 Uganda: 123 Denmark: 100 Greece: 84 Singapore: 43 United Kingdom: 35 Switzerland: 24 Poland: 18 Nigeria: 9	LY-CoV555 (LY3819253, bamlanivimab) VIR-7831 (sotrovimab, Xevudy®) BRII-196 plus BRII-198 (amubarvimab/ romlusevimab) AZD7442 (tixagevimab/ cilgavimab, Evusheld®) MP0420 (ensovibep) PF-07304814
ACTIV-3b/ TESICO (Therapeutics for Severely Ill Inpatients With COVID-19) NCT04843761	Master protocol to evaluate the safety and efficacy of investigational agents aimed at improving outcomes for participants with acute respiratory failure related to COVID-19	April 2021 to May 2022 473 participants	US: 473	Aviptadil (Vasoactive Intestinal Peptide) Remdesivir (Veklury®)
ACTIV-3 Substudy/VATICO (Vaccination for Recovered Inpatients with COVID-19) NCT04969250	Open-label trial with a factorial design (one vs. two doses, and immediate vs. deferred vaccination) in a subset of ACTIV-3/INSIGHT014/TICO participants who receive one of two mRNA vaccines	August 2021 to February 2022 66 participants	US: 28 Uganda: 20 Spain: 14 Switzerland: 4	Moderna mRNA-1273 vaccine (Spikevax®) Pfizer BNT162b2 vaccine (Comirnaty®)

Abbreviations: ACTIV = Accelerating COVID-19 Therapeutic Interventions and Vaccines; DCR = Division of Clinical Research; COVID = coronavirus disease; hIVIG = (anti-coronavirus) hyperimmune intravenous immunoglobulin; INSIGHT = International Network for Strategic Initiatives in Global HIV Trials; mRNA = messenger ribonucleic acid; NCT = National Clinical Trial; OTAC = Outpatient Treatment with Anti Coronavirus Immunoglobulin; TESICO = Therapeutics for Severely Ill Inpatients with COVID-19; TICO = Therapeutics for Inpatients with COVID-19; VATICO, Vaccination Strategies for Recovered Inpatients with COVID-19.

## Implementation of GPP-EP guidelines in DCR-funded ACTIV COVID-19 trials

The challenges of conducting clinical research evolved as the pandemic evolved, as did the genesis of fears and potential risk-benefit analyses of participating in clinical research. Very early in the pandemic, there was chaos and disorganization: there was not enough personal protective equipment (PPE) and healthcare workers rationed and reused PPE; people died without family members present; temporary morgues were established on hospital grounds to deal with the large numbers of deaths; healthcare workers were under enormous stress [20,21]; there was no coordinated US research agenda; pharmaceutical companies had already started doing trials; and many underpowered studies utilized precious research capacity [11,22]. During that time, over 2,000 patients were dying per day in the US [23]. The Therapeutics Lead for the US government’s OWS and the NIH Director wrote to the research leaderships at sites conducting ACTIV-3 and the ACTIV-associated DCR-funded study “Inpatient Treatment with Anti-Coronavirus Immunoglobulin (ITAC)” (see Supplementary Figure 1). They stressed the high priority of these studies to OWS and NIH and requested as much support for these trials as possible, including:

- Prioritization of these trials for rapid contractual and institutional approvals
- Provision of space and personnel to facilitate study product/placebo infusions
- Dedication of rapid testing resources as available

In April 2021, one year after the ACTIV program was launched, a GPP Stakeholder Team (GPP Team) was formed to develop a plan for materials and activities for Outpatient Treatment with Anti-Coronavirus Immunoglobulin (OTAC), ACTIV-3, -3 substudy, and -3b, and to review and provide feedback on draft materials and activity plans. At the time, ACTIV-3 and -3b were already enrolling, protocol development for OTAC was complete, and the ACTIV-3 substudy was in the final stages of protocol development. Engagement with local communities, as recommended by GPP-EP guidelines, was not planned because sites were overwhelmed caring for patients, there was no specific “COVID community” since everyone was at risk, and in-person gatherings were generally prohibited. The GPP Team did not engage with regulators or national COVID-19 response teams as this was left to DHHS/NIH staff in the US, and to staff at the ICCs and sites outside the US, to manage. INSIGHT had a long-standing, independent, committed network-level Community Advisory Board (CAB) comprised of community members whose role was to represent the research-related perspectives of trial participants in the diverse sites and communities within the network.

The GPP Team consisted of representatives from the ICCs, sites, the SDMC, the INSIGHT CAB, and DCR. The site staff on the GPP Team had extensive experience in the conduct of clinical trials and brought real-time experience with recruitment and implementation challenges for COVID-19 trials. The CAB representatives were highly experienced in reviewing and providing input on protocols, informed consent documents, and other participant-facing materials. Collectively, GPP Team members came from multiple countries, were involved in all four studies, and had considerable expertise and experience in developing and using participant-facing materials.

**Figure 2.**
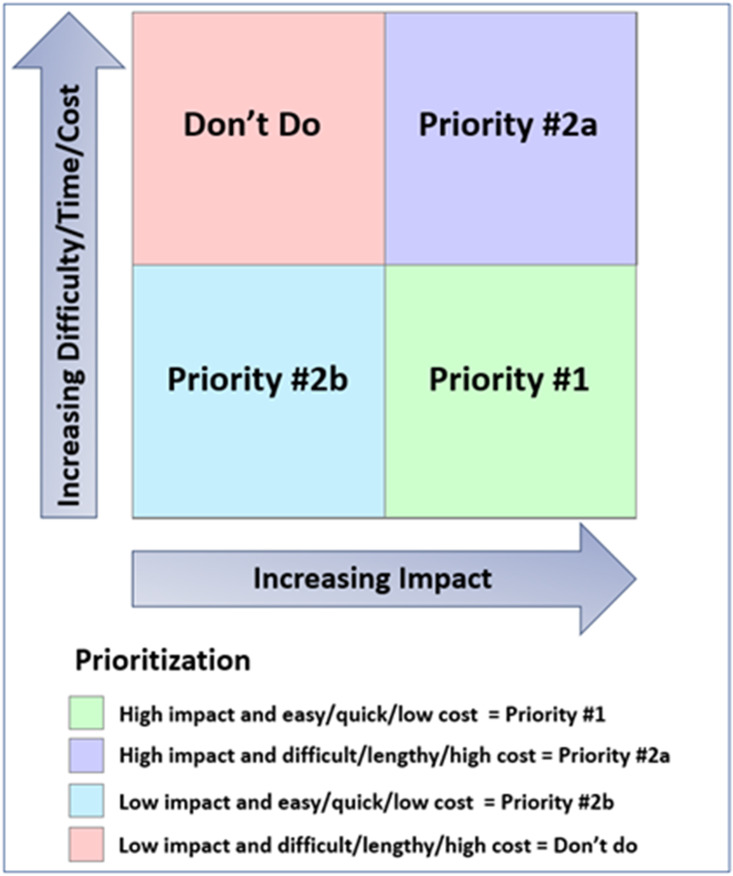
Framework for prioritization of potential study materials and activities. Matrix used to prioritize activities and materials for study implementation based on anticipated impact and predicted difficulty, time, and cost for development, approval, and production.

The GPP Team held a series of discussions to identify activities and materials needed for study recruitment and implementation. They used a matrix (Fig. 2) to prioritize material development based on anticipated impact and projected difficulty, time, and cost for production.

The GPP Team identified the following top three priorities:

1. **Addressing Recruitment Challenges with Participant-Facing Videos, Brochures, Flyers, and Other Materials.** Early in 2021, in the face of the escalating sociopolitical climate of fear, misinformation, and scientific skepticism, sites were reporting that a growing proportion of eligible participants were declining to enroll. Some patients declined because they did not want to add anything else to their treatment plan or they did not want anything experimental; several did not believe COVID-19 existed [24,25] and did not wish to participate in a COVID-19 study. Unlike earlier in the pandemic, many were younger patients who didn't expect to get very sick and believed that they would recover, so were not interested in participating in a trial. In addition, hospitals were saturated, and hospital staff were overworked [26,27]. Some sites were screening dozens of patients to enroll a single participant in both outpatient and inpatient studies. In response, the GPP Team decided that developing videos, brochures, flyers, and other materials was the top priority. GPP Team members expected this effort would have medium-to-high impact and low-to-medium difficulty/cost/effort to produce, with videos having a greater impact and difficulty/cost/effort than hard-copy materials.
2. **Sharing Proven Site Resources via a Study Recruitment and Implementation Toolkit.** GPP Team members thought a centrally available toolkit of resources would enable sites to exchange effective tools, reducing the burden of producing new materials. They determined this could be high impact with low difficulty/cost/effort to implement. Ultimately, requests for sites to share materials they found useful were met with little response, and the toolkit was abandoned.
3. **Helping Participants Adhere to Study Requirements with a Schedule of Study Visits.** GPP Team members thought that a personalized schedule of study visits and activities, with graphics, may improve follow-up and increase participant satisfaction and adherence to study procedures. Members expected this would have medium impact with very low difficulty/cost/effort to produce. Sites could already produce a visit schedule from the clinical database that was intended for site use, was only in English, had medical terminology, and lacked graphics. The GPP Team determined that it would be difficult to ensure ongoing synchronization with the schedules from the clinical database. This effort was abandoned.

Concurrent with this prioritization process, discussions began with the CombatCOVID program, which was funded by the US DHHS. Through these discussions, the GPP Team agreed that study-specific web pages and overview videos should also be prioritized. A proposal was drafted that included the rationale, timelines, high-level budget estimates, staff requirements, and other resources necessary for developing each of the prioritized materials and activities. DCR endorsed all aspects of the proposal and allocated funds and other resources for their expeditious implementation.

## Process for developing and producing materials

**Figure 3.**
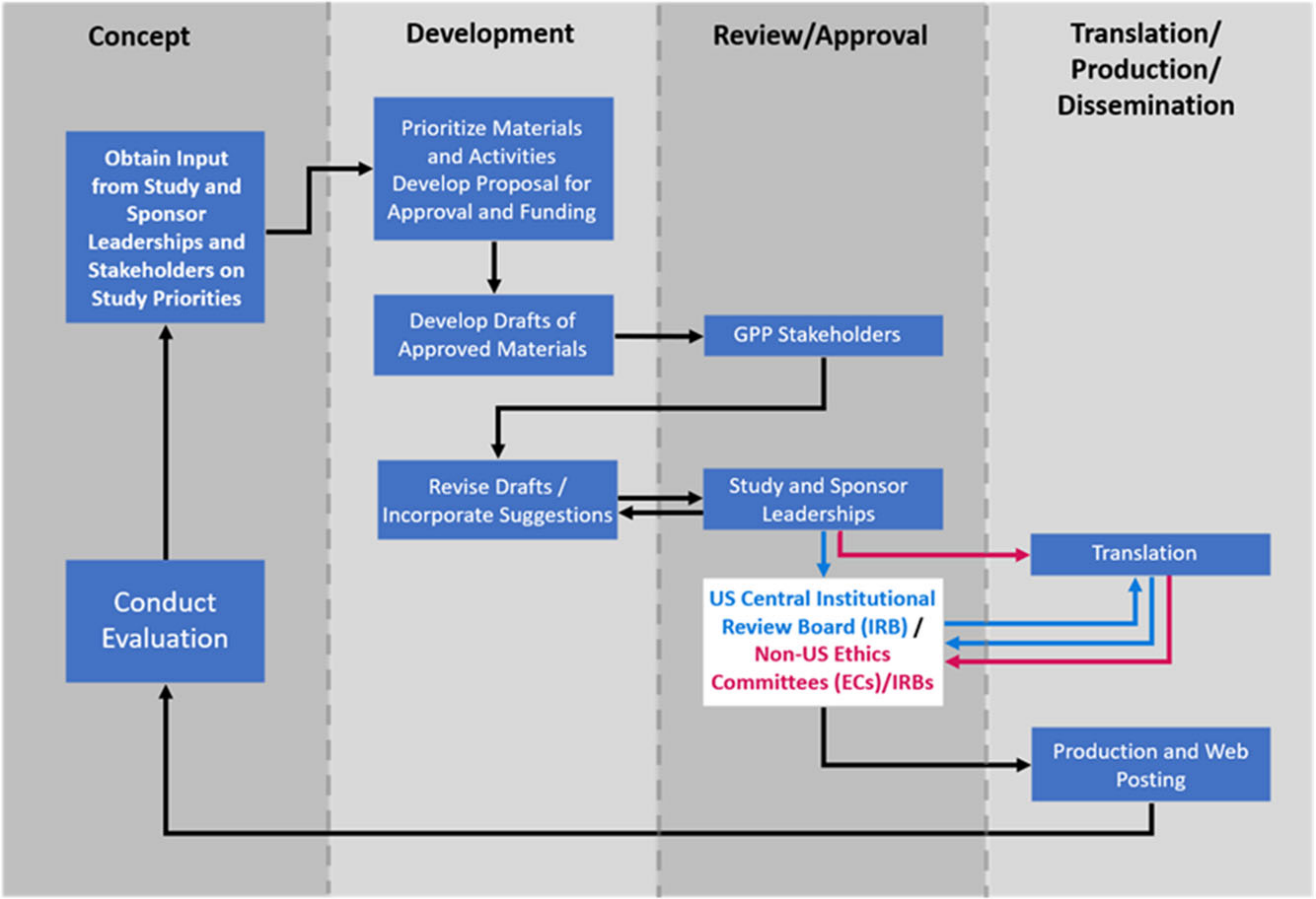
Process for development and production of participant-facing materials. The process for developing and producing material included a continuous interaction with stakeholders from conceptualization, development, review, and approval through translation. Final materials were posted on the password-protected International Network for Strategic Initiatives in Global HIV Trials (INSIGHT) member website where sites could download them at any time. Sites could choose to download the items to a computer or mobile device, print them locally, and/or request that they be printed and shipped to them. The study overview videos and flipbooks were also posted for public access on the CombatCOVID study-specific web pages and, subsequently, on the National Institute of Allergy and Infectious Diseases (NIAID) study-specific web pages. Abbreviations: EC = ethics committee; GPP = Good Participatory Practices.

Initial material designs and drafts were created through collaboration among study leadership, DCR scientists, and graphic designers (Fig. 3). They used the NIAID HIV Language Guide [28] and created and continually updated a glossary of preferred terms and phrases (Supplementary Figure 2). Attention was paid to providing materials that were accessible to diverse populations, including people with hearing and vision impairment, and suited to various learning preferences. Materials were developed in English. Drafts were reviewed by the GPP Team and INSIGHT CAB to ensure that participant-facing materials were easy to read and understand and used diverse, inclusive, and culturally appropriate language, graphics, and images. Materials were assessed using appropriate software to ensure accessibility and a reading level of US Grade 8 (approximately 13–14 years of age) or lower [29]. Quick Response (QR) codes were generated for study-specific web pages and placed on participant-facing materials to facilitate navigation to those web pages and to provide website analytics. Recruitment materials contained placeholders for customization with site-specific contact information. After GPP Team and CAB input, materials were reviewed by the protocol and sponsor leaderships to ensure scientific accuracy and fidelity to protocol requirements. Once the materials were approved, they were submitted for ethics committee (EC)/institutional review board (IRB) reviews. A central IRB (cIRB) was used for US sites. The ICCs were responsible for obtaining all required EC/IRB approvals for other countries. Materials were translated by a certified translator into the languages requested by the ICCs. Collectively, participant-facing materials were translated into 35 languages. Translated materials were submitted to the US cIRB or national and local ECs/IRBs as appropriate.

The GPP Team, study leadership, and sponsor leadership reviewed draft video scripts with proposed visuals. These were submitted for provisional cIRB approval prior to filming and creating the final videos. The same groups reviewed the final videos before they were submitted for cIRB review.

Because COVID-19 required infection-control procedures and isolating participants, the materials provided to sites needed to account for these challenges. This excluded durable materials that could not be sanitized between uses. Acceptable formats included single-use paper, vinyl paper and laminated copies that could be sanitized, and electronic documents. Several sites requested electronic tablets to assist with providing participant-facing materials to potential participants, families, caregivers, and legally authorized representatives (LARs). Tablets that could be sanitized were pre-loaded with the materials and shipped to the requesting sites.

Final materials were posted on the password-protected INSIGHT member website where sites could download materials at any time. Sites could choose to download the items to a computer or mobile device, print them locally, and/or request that they be printed and shipped to them. The study overview videos and flipbooks were also posted for public access on the CombatCOVID and, subsequently, on the NIAID study-specific web pages. Videos could be streamed via the respective YouTube channels.

## Materials produced

According to GPP-EP Guidelines “Trial accrual, follow-up, and exit activities include the recruitment, screening, enrolment, follow-up, and exit of trial participants” [8]. The materials were developed primarily to engage with various study stakeholders at sites, including ICUs. They were designed to align with GPP principles to promote understanding and acceptance of a study while emphasizing autonomy, to facilitate study conduct, to thank participants, and to provide participants with study results at the end of the study. In developing materials and providing tools (electronic tablets), the GPP Team considered the characteristics of the eligible trial population, as well as site needs, infrastructure, and resources. Specific materials that were developed are summarized below and in Table 2. Additional details and examples of the materials are located in the Supplementary Material.

**Table 2.** Overview of materials developed by the GPP team for the DCR-funded COVID-19 trials. The table provides information about the materials developed for study recruitment, implementation, and exit applying Good Participatory Practice guidelines for trials of (re-) emerging pathogens (GPP-EP)

Material	Content	Format and Distribution Media	Purpose	IRB Approved	Intended Distribution Targets	Studies Developed For:			
						Inpatient		Outpatient	
						ACTIV-3 TICO	ACTIV-3b TESICO	ACTIV-3 Substudy VATICO	OTAC
**Posters/Flyers** Expanded information about the study (Supplementary Figure 3)	Study name and logo, eligible population, aim of study, duration of study participation, standard of care statement, URL and QR code for the study-specific web page, customizable local site contact information	PDF, paper, vinyl/laminated paper (multiple sizes) INSIGHT member web site	Recruitment	Yes	Hospitals, clinics, COVID-19 testing sites, potential participants and their families*		●		●
**Recruitment Flyers** One-page format, basic information about study (Supplementary Figure 4)	Study name and logo, eligible population, information about testing new treatment for COVID-19, QR code for the study-specific web page, customizable local site contact information	PDF, paper INSIGHT member website	Recruitment	Yes	Clinics, testing sites, potential participants and their families*				●
**Yard Signs and Social Media Messages** Large-size format (Supplementary Figure 5)	Study name and logo, information about testing new treatment for COVID-19, URL and QR code for the study-specific web page, central email address for study information, customizable local site contact information	PDF, plastic-coated cardboard on metal stand Yard sign shipped to sites image posted to INSIGHT member website	Recruitment	Yes	Study sites, potential participants and their families*, social media users, including followers of the study site				●
**Social Media Messages and Images** Short messages, images, and graphics (Supplementary Figures 3, 5, 6)	Study name, statement about seeking study volunteers, eligible population (short), treatment name (hIVIG), URL to NIAID study-specific website, customizable local site contact information, hashtags used #volunteers and #hIVIG Images of posters and yard signs	PNG (images), digital INSIGHT member website Social media accounts of NIAID and study sites	Recruitment	Yes	Social media users, including followers of study site, clinic, and NIAID social media accounts				●
**Email Messages** Email text (Supplementary Figure 7)	Eligible population, statement about seeking study volunteers, study drug name (hIVIG), URL for NIAID study-specific website	Word document INSIGHT member website NIAID and site email distribution lists, listservs	Recruitment	Yes	Subscribers to NIAID and study site listservs				●
**Web pages on the** **CombatCOVID and, Subsequently, NIAID Websites** Detailed information about studies (Supplementary Figure 8)	Study name; eligible population; aim of the study; schedule of study participation activities; information about study drug(s), randomization, and placebo; URL for clinicaltrials.gov study web page section with locations of sites and contact information; email address for more information; and study-specific flipbooks, overview videos, and video flipbooks	Web page Link from INSIGHT member website	Recruitment	Yes	Potential participants and their families*, healthcare providers and other non-study medical personnel	●	●		●
**Recruitment Letters for Potential Participants** Three versions: One- and two-page text formats, and a 2-page graphic format (Supplementary Figures 9A-C)	Letter to ACTIV-3/TICO participants about participating in ACTIV-3b/VATICO in Q&A format. Study name, aim of study, information about mRNA COVID-19 vaccines, study vaccination and visit schedule, participant’s time commitment	PDF, paper INSIGHT member website	Recruitment	Yes	ACTIV-3 TICO participants			●	
**Study Overview Videos** 5- to 10-minute length, detailed study information in simple language using graphics, animation, and on-screen narration by a study team member	Study name, study aims, information about study drug (s), potential risks and benefits, side effects, randomization, placebo, schedule of study participation and activities, funding source, standard-of -care statement, national study phone number and CombatCOVID website URL	MP4 video file Electronic file on tablet CombatCOVID and NIAID web pages YouTube channels INSIGHT member website	Recruitment, aid to the informed consent process	Yes	Potential participants and their families*	●	●	●	●
**Flipbooks** Detailed study information in simple language, Q&A format, heavy use of graphics (Supplementary Figure 10)	Study name, study aims, local standard of care statement, general information about clinical trial, need for informed consent, study drugs, potential risks and benefits, side effects, placebo, randomization, schedule of study participation and activities, pregnancy and contraception requirements, protection of privacy, use of samples and data collected, funding source, URLs for study-specific information on EudraCT and clinicaltrials.gov	PDF (developed in PowerPoint) Hard copy vinyl paper in pocketless binders Electronic file on tablet CombatCOVID and NIAID web pages YouTube channels INSIGHT member website	Recruitment, aid to the informed consent process	Yes	Potential participants and their families*	●	●	●	●
**Audio Flipbook** Flipbook read by narrator (12- to 20-minutes long)	Same as flipbook with added narration – slides have to be advanced manually	PowerPoint Electronic file on tablet INSIGHT member website	Recruitment, aid to the informed consent process	Yes	Potential participants and their families*	●	●	●	●
**Video Flipbook** 12 to 20-minute video of flipbook with narration	Same as flipbook with added narration – slides advance automatically	MP4 video file Electronic file on tablet CombatCOVID and NIAID web pages YouTube channels INSIGHT member website	Recruitment, aid to the informed consent process	Yes	Potential participants and their families*	●	●	●	●
**Participant Brochure** Trifold, double sided, single sheet participant instructions with graphics for monitoring and recording temperature and oxygen saturation levels at home as required by the study (Supplementary Figure 11)	Study name, Q&A format instructions, instructions for measuring temperature and peripheral oxygen saturation levels, instructions for what to do if temperature or oxygen saturation is out of range, customizable contact information, and table for recording temperature and peripheral oxygen saturation measurements	PDF, paper INSIGHT member website	Study conduct	Yes	Participants				●
**Thank-You Letters** Customizable letters with information on study enrollment and results (Supplementary Figure 12)	Thanked participant, stressed importance of research, described final study enrollment and basic study results, and provided instructions on how to get additional information	Word document, paper INSIGHT member website	Study exit	Yes	Participants	●		●	
**Letter for Participants to Share with mRNA COVID-19 Vaccine Providers** Letter format. One page is for the participant to keep and the other pages are for the participant to give to the vaccine provider. (Supplementary Figure 13)	**Participant letter:** study name, thank-you statement, study aim, customizable date range for study vaccination, customizable study coordinator’s and site investigator’s contact information	Word document, paper INSIGHT member website	Study conduct	Yes	Participants			●	
	**Provider letter:** study name, overview of study objectives, study information in Q&A format, information about randomization groups, statement of which group the participant was randomized to with customizable date range for study vaccination, customizable site investigator’s contact information	Word document, paper INSIGHT member website	Study conduct	N/A	Participants and their COVID-19 vaccine providers			●	
**Proof of Recovery Letter and mRNA COVID-19 Vaccination Card** Two-page document (Supplementary Figure 14)	Customizable document provided to sites to complete, print on local letterhead, and give to participants indicating recovery from COVID-19 and clearance to end isolation; vaccination card with record of study participation and date(s) of vaccination	Word document, paper INSIGHT member website	Record of recovery from COVID-19 and vaccine study participation to mitigate constraints on unvaccinated persons	Yes	For participant to show to anyone who required proof of recovery or vaccination			●	
**Protocol Quick Reference Guide** Two-page format, key points about the study (Supplementary Figure 15)	Study name, diagram of study design, timeline of protocol assessments, primary objective and endpoint, clinical assessment scale, detailed inclusion and exclusion criteria, customizable local site contact information	PDF, paper INSIGHT member website	Recruitment, study conduct, staff training	N/A	Study staff, healthcare providers and other non-study medical personnel				●
**Study Summary Presentation** Summary of study for medical colleagues (Supplementary Figure 16)	Study name, scientific rationale, hypothesis, study product, potential advantages of study product over convalescent plasma and monoclonal antibodies, primary objective and endpoint, study design, inclusion and exclusion criteria, aim of the study, schedule of assessments, and duration of study participation	PowerPoint presentation INSIGHT member website	Recruitment, staff training	N/A	Study staff, healthcare providers and other non-study medical personnel				●
**FAQ: Hyperimmune IVIG in OTAC** Q&A format, medical language (Supplementary Figure 17)	Study name and hypothesis, information about hIVIG and rationale for studying it, potential activity against SARS-CoV-2 variants, potential advantages over convalescent plasma and monoclonal antibodies, concomitant use of monoclonal antibodies, and relevance to different populations globally	PDF, paper INSIGHT member website	Recruitment and study conduct	N/A	Study staff				●
**Dear Colleague Letter** One-page format, medical language (Supplementary Figure 18)	Study name, eligible population, study aims, information about hIVIG, schedule of study participation activities, customizable site staff contact information	Word document, paper INSIGHT member website	Recruitment	N/A	Healthcare providers and colleagues of OTAC clinicians				●

Abbreviations: ACTIV = Accelerating COVID-19 Therapeutic Interventions and Vaccines; COVID = coronavirus disease; DCR = Division of Clinical Research; EudraCT = European Union Drug Regulating Authorities Clinical Trials Database; GPP = Good Participatory Practice; GPP-EP = Good Participatory Practice guidelines for trials of (re-) Emerging Pathogens; FAQ = frequently asked questions; hIVIG = (anti-coronavirus) hyperimmune intravenous immunoglobulin; INSIGHT = International Network for Strategic Initiatives in Global HIV Trials; IRB = Institutional Review Board; IVIG = intravenous immunoglobulin; mRNA = messenger ribonucleic acid; MP4 = MPEG-4 (motion picture experts group-4); N/A = not applicable; NIAID = (United States) National Institute of Allergy and Infectious Diseases; OTAC = Outpatient Treatment with Anti-Coronavirus Immunoglobulin; PDF = portable document format; PNG = portable network graphic; Q&A = question and answer; QR = quick response; SARS-CoV-2 = severe acute respiratory syndrome coronavirus 2; TESICO = Therapeutics for Severely Ill Inpatients With COVID-19; TICO = Therapeutics for Inpatients with COVID-19; URL = uniform resource locator; VATICO = Vaccination for Recovered Inpatients with COVID-19.

*Families include caregivers and legally authorized representatives (LARs).

### Participant-facing materials

#### Recruitment materials

These materials included posters, flyers, yard signs, social media messages and images, email messages, study-specific web pages on the CombatCOVID and NIAID websites, and recruitment letters (Supplementary Figures 3-9). They were designed to draw attention to and solicit interest in the respective studies by eligible populations and their families, caregivers, LARs, and healthcare providers. Hard copies of some materials could be placed strategically around the hospital and doctors’ offices, outside site facilities and COVID-19 testing locations, and elsewhere in the community. Social media messages and images were designed for NIAID and sites to post on their social media accounts. Email messages were developed for NIAID and site email listservs and other group distribution mechanisms. All these materials were customizable with local site contact information. The web pages served as central hubs for study information and participant-facing resources in English and Spanish. They were located on websites that also provided information about COVID-19 for healthcare professionals and the public.

Recruitment into the ACTIV-3 substudy (VATICO) was limited to ACTIV-3 study participants. Thus, recruitment materials intended for the general public were not needed, and no web pages, yard signs, posters, or social media messages were developed for the ACTIV-3 substudy. Recruitment materials for the substudy that site staff could share with ACTIV-3 participants included: study overview videos; long, short, and graphic versions of recruitment letters (Supplementary Figures 9 A-C); and flipbooks available in hard copy as well as audio, video, and standard electronic formats (Supplementary Figure 10).

#### Materials to assist with the informed consent process

Study-specific overview videos and flipbooks in various formats were developed to assist with providing the comprehensive information necessary for potential participants’, families’, or LARs’ full understanding of the respective study during the consent process [30, 31]. These materials relied heavily on the use of graphics, audio, video, and/or animation to deliver information for better comprehension by different types of learners or in diverse populations (e.g., people who were ill, had low literacy levels, or had visual and/or hearing impairment). The GPP Team focused on ensuring appropriate reading level, simplicity, clarity, accessibility, ethnic and cultural diversity, as well as accommodating the range of site information-technology resources and infection-control policies. The study overview videos were generally easy to share via websites, email, YouTube, and other platforms. Flipbooks were prepared as PowerPoint presentations with each slide containing study information in the lower half and an accompanying graphic in the upper half (Supplementary Figure 10). Flipbooks were reorganized as the ACTIV-3 platform trial evolved with investigational products being added and removed. The US cIRB approved a proposal to include optional modules in the flipbooks that could be removed. The main module included slides addressing the master portion of the adaptive platform: study aims, study visits and procedures, and contraceptive requirements. Separate modules contained slides about placebo, randomization, and the various investigational products – sections that could be added or removed by sites depending on study drug availability. A final module contained information about data and specimen use, privacy, and where to find information about study compensation and ancillary care costs. If human genetic testing was an option for the study, an additional module addressed this.

#### Trial conduct and exit materials

Trial conduct materials were designed to help participants during follow-up. For OTAC, a participant brochure was developed with instructions for performing and recording oral temperature and pulse oximetry measurements at home. It also included information about what to do in case temperature or peripheral oxygen saturation was out of range, or if the participant experienced severe or worsening COVID-19 symptoms, as well as customizable site contact information (Supplementary Figure 11). The brochure was illustrated, simple to follow, and helped to avoid mistakes and protocol deviations. Trial exit materials were created to be provided to participants at study conclusion. Thank-you letters expressed gratitude for participants’ commitment to the study and provided a summary of the results of the study as well as instructions about how to contact the study site for additional information (Supplementary Figure 12).

ACTIV-3 substudy investigated COVID-19 vaccination schedules that differed from the schedule generally recommended. Participants received either one or two doses of vaccine, initiated immediately upon randomization or deferred for 12 weeks. This posed challenges for participants who might face restrictions because they were not considered “fully vaccinated.” Addressing this challenge required unique participant materials: a letter for participants to share with COVID-19 vaccine providers explaining the ACTIV-3 substudy and its vaccination schedules, and a proof of recovery letter and COVID-19 vaccination card to enable participants to return to school and work, travel, and enter premises restricted to people who were fully vaccinated (Supplementary Figures 13 and 14).

### Materials for study staff and non-study medical personnel

Several materials were developed to inform study staff and non-study medical personnel about the studies, for example at site and faculty meetings, as a quick reference, and for orienting new or temporary staff. They presented information about key aspects of the specific study and underlying science in an understandable and easy-to-remember format, highlighting important points and answering common questions medical providers might have about the trial. These materials included quick reference guides about the protocol, study summary PowerPoint presentations, and frequently asked questions on hIVIG for OTAC (Supplementary Figures 15–17). A “Dear Colleague” template letter was designed to provide information about the study and solicit referrals from local medical providers who might encounter potential participants (Supplementary Figure 18).

## Evaluation of the materials

To evaluate materials, from July 14 to August 3, 2022, the GPP Team conducted an anonymous survey of study site personnel (Supplementary Figure 19). The intention of the survey was to learn about how study materials were used, their value, and to understand operational processes to optimize resource allocation and intensify efforts toward new and ongoing studies. The survey link was sent to the ICCs to distribute to their respective sites. A few ICCs and SCCs responded on behalf of their sites. As of July 31, 2022, 134 sites had enrolled 3,530 participants in the four DCR-funded COVID-19 studies, with many sites conducting more than one study. Responses represented a total of 119 sites, with ICC and SCC responses representing 40 sites.

Usefulness was measured using a 5-point rating scale (not useful, minimally useful, somewhat useful, very useful, extremely useful) and not used. The survey requested the sites to rank the top three materials for each study they conducted, based on which materials were most helpful, second most helpful, and third most helpful. Open-ended questions were asked about successes and challenges they encountered. Finally, to understand the regulatory challenges for participant-facing materials to be approved, the survey included questions about EC/IRB approvals.

According to the survey responses, most of the US sites used a cIRB for review of participant-facing materials; over half required additional or local site EC/IRB approval for participant-facing materials. Fewer than half of the non-US sites reported that their local EC/IRB deferred to a central EC/IRB for approvals. Table 3 summarizes study-specific survey results.

**Table 3.** Summary of results from the survey of study site personnel about the study-specific materials provided. Table shows the materials that sites found most useful for each of the four COVID-19 trials funded by the Division of Clinical Research, National Institute of Allergy and Infectious Diseases, National Institutes of Health, United States government

Study	Main uses for materials and tools	Materials and tools rated “★★★★★ - *extremely useful*”	Materials and tools ranked as most helpful	Materials and tools ranked as second most helpful	Materials and tools ranked as third most helpful
OTAC	Inform potential participants, families, and caregivers about the study Inform or educate site staff Solicit referrals from healthcare providers in the institution	Flipbook (PDF) Study Summary PowerPoint Presentation FAQ: “hIVIG in OTAC” Flyers Protocol Quick Reference Guide Dear Colleague Letter CombatCOVID/NIAID Web pages Participant brochure “Checking Your Temperature and Oxygen Levels at Home”	Flipbook	Study Summary PowerPoint Presentation	Protocol Quick Reference Guide
ACTIV-3/ TICO	Inform potential participants, families, and caregivers about the study Assist with participant consent Inform or educate site staff	Flipbook (PDF, audio, and video) Study overview video Day 28 VATICO recruitment letter (pictures, short, and long versions) CombatCOVID/NIAID web pages Electronic tablets	Flipbook	Study overview video	Flipbook (video version)
ACTIV-3b/ TESICO	Inform potential participants, families, or caregivers about the study Inform or educate site staff Assist with participant consent	Flipbook (PDF, audio, and video) Study overview video Poster Electronic tablet	Flipbook	Study overview video	Electronic tablet Poster
ACTIV-3 Substudy/ VATICO	Inform or educate site staff Inform potential participants, families, or caregivers Assist with participant consent	Flipbook (PDF and audio) Recruitment letter Proof of recovery letter and vaccination card Participant letter to share with vaccine providers	Participant letter and study information	Participant letter for vaccination centers	Flipbook

Abbreviations: ACTIV = Accelerating COVID-19 Therapeutic Interventions and Vaccines; COVID = coronavirus disease; FAQ = frequently asked questions; hIVIG = (anti-coronavirus) hyperimmune intravenous immunoglobulin; NIAID = National Institute of Allergy and Infectious Diseases; OTAC = Outpatient Treatment with Anti-Coronavirus Immunoglobulin; PDF = portable document format; TESICO = Therapeutics for Severely Ill Inpatients With COVID-19; TICO = Therapeutics for Inpatients with COVID-19; VATICO = Vaccination for Recovered Inpatients with COVID-19.

**Figure 4.**
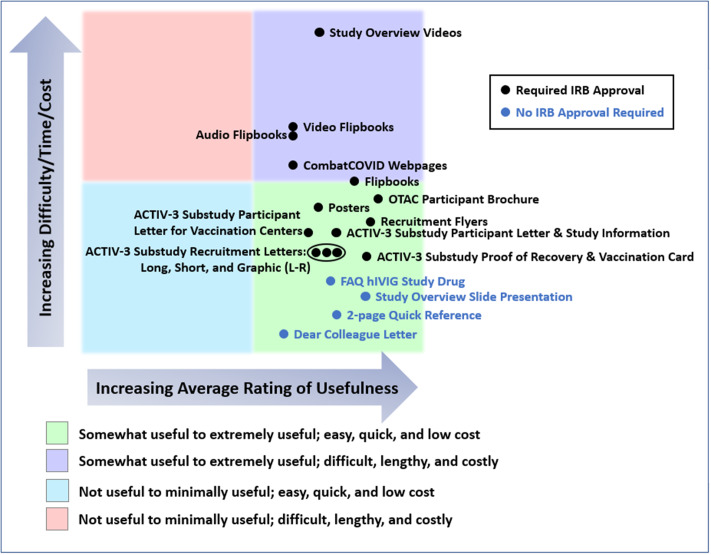
Average usefulness of the materials provided for ACTIV-3, -3 substudy, -3b, and OTAC, and estimates of resources used in producing them. This chart presents a rough estimate of the relative costs, difficulty, and time required to produce each type of material, including securing United States central institutional review board approval, as appropriate. Survey respondents were requested to rate the usefulness of each type of material for all studies in which they used them, or indicate if they had not used the material in particular studies. Usefulness of each material for each study was rated on a 5-point scale (not useful, minimally useful, somewhat useful, very useful, and extremely useful). The chart also shows the combined average ratings of materials for all four studies provided by the international coordinating centers (ICCs), site coordinating centers (SCCs), or sites that used the materials. Abbreviations: ACTIV = Accelerating COVID-19 Therapeutic Interventions and Vaccines; COVID = coronavirus disease; FAQ = frequently asked questions; hIVIG = (anti-coronavirus) hyperimmune intravenous immunoglobulin; IRB = Institutional Review Board; L-R = Left to Right; OTAC = Outpatient Treatment with Anti-coronavirus Immunoglobulin.

In Fig. 4, we have attempted to provide a rough estimate of the costs, difficulty, and time required to produce each group of materials relative to the others. Specific metrics related to cost, effort, or timelines were not measured or tracked. Using the 5-point rating scale, we also show the combined average ratings of materials for all studies provided by the ICCs, SCCs, or sites that used the materials.

All materials developed were somewhat to extremely useful. Comparing inpatient and outpatient studies, audio and video materials were slightly more highly rated for inpatient studies, while web pages were more highly rated for the outpatient study, OTAC. The most preferred format for the recruitment letter for the outpatient ACTIV-3 substudy (VATICO) was the graphic format followed by the short form and finally the long form.

Respondents were asked to provide information to confirm their site used the materials produced by the GPP Team and what characteristics possibly caused rejection of the materials by a site’s national and/or local EC/IRB. It was important to have diverse populations represented in the photographs and graphics. In Denmark, the EC did not approve the materials with photographs of diverse people because they did not represent the local community; however, the Danish ECs approved other materials like flipbooks with demographic diversity depicted in cartoons. The Greece SCC produced a poster locally because the photographs in the posters developed were not considered representative of the population. The EC/IRB in Greece approved both the locally produced poster and the translated flipbooks. Some sites noted that black-and-white paper and lower-tech materials may be more acceptable to their populations than high-quality, colorful, glossy materials. Some patients did not have updated devices to be able to view some of the electronic materials.

Sites were asked to identify challenges in recruitment, consenting, and study conduct that the materials may not have addressed. Recruitment challenges listed included patients hesitant to participate in clinical trials due to doubts concerning their COVID-19 diagnosis, desire to receive active treatment and not placebo, wanting to involve family members yet families were not allowed to visit the hospital, and difficulties anticipated with completing follow-up. In the ACTIV-3 substudy, some patients were vaccine-hesitant or wanted to be vaccinated as soon as possible (before the timeframe for the group randomized to a 12-week deferral).

Concerning study conduct challenges, some sites specified institutional barriers related to infection control. For example, some institutions did not have designated areas where participants with COVID-19 could receive the clinical trial infusion for OTAC or complete in-person follow-up visits and/or blood draws. Other institutional barriers included conducting competing studies at the same site, delays in contractual agreements that continued until the study closed to enrollment, and slow internet in hospitals and clinics that made it difficult to stream large documents such as audio PowerPoint presentations and MP4 video files. Responses related to sites in lower-resourced settings included not having medical supplies required to prepare experimental drugs which delayed opening to enrollment . Sites expressed the need to have meetings among site pharmacists and the network specialist pharmacist to discuss product management issues.

## Discussion and lessons learned

COVID-19 has required continuous adaptation in clinical research. Utilizing GPP principles has facilitated trial conduct and consistency across trials even as rapid changes were made in response to a fast-moving pandemic. This manuscript presents, for the first time, the application of GPP-EP guidelines in creating resources for supporting the implementation of multiple clinical trials from a global network viewpoint amid the COVID-19 pandemic. The centralized development, production, and translation of materials reduced site burden. Stakeholder involvement in the prioritization and review of materials proved very helpful and materials were all deemed somewhat to extremely useful by the sites. Leveraging GPP to develop study materials during a pandemic poses various challenges: limited in-person interactions, limited time for planning for GPP and stakeholder engagement, limited time and ability to engage marginalized populations, public fear, and healthcare worker burnout from the overwork and stress caused by the pandemic. The high level of misinformation, rumor, and distrust of science and health care was not anticipated.

Better planning and preparation for stakeholder engagement and implementation of GPP-EP guidelines for future health emergencies is essential. This should include post-COVID-19 pandemic engagement with stakeholders – including regulatory authorities; national emergency response agencies; professional, healthcare, patient advocacy, faith-based and community organizations; and the media – to build trusting relationships, develop communication channels, exchange information, and forge agreements in preparation for the next public health emergency. Our experience in developing the materials along with the results of the evaluation survey provide several key lessons.

### Preparation and timeliness are important

It is vital to plan now to incorporate GPP-EP into the research response to the next public health emergency and to include stakeholder input early in the development of the research agenda and protocol concepts. This will help to anticipate challenges and develop strategies to prevent or address them. As mentioned previously, the ACTIV public-private partnership was formed in the wake of chaos and disorganization to develop a US national research response to identify effective medical countermeasures against COVID-19. The GPP Team first met a full year after the ACTIV initiative was established, the ITAC trial had completed enrollment and follow-up for the primary endpoints, and enrollment was underway or about to begin for the ACTIV-3, ACTIV-3 substudy, ACTIV-3b, and OTAC trials. This precluded input from the GPP Team during the protocol development process and meant that the Team was reacting to problems that had arisen rather than anticipating and preventing them to the extent possible.

Preparations and plans for implementing GPP-EP from the outset of the public health emergency and throughout the study life cycle should be established prior to the next public health emergency. This should include identification of necessary resources and development of practical mechanisms for tracking and contemporaneous evaluation – utilizing easily measurable and meaningful metrics – of stakeholder engagement and the impact of GPP materials and activities in a variety of global settings. This should enable well-informed, timely strategic decisions.

Furthermore, feedback received from the non-US sites indicated materials should be made available at the same time as the informed consent form. This will allow sites to secure translations and other site-specific approvals, add institutional logos, customize with local site information, or edit to meet local requirements, and submit them to their EC/IRB concurrently with the protocol and informed consent. Providing materials early in the process saves time and money, allows more time to train staff, and expedites study implementation.

### Materials and strategies should be tailored to specific needs and audiences

Timely referral is crucial in studies of acute illnesses with narrow eligibility windows. Strategies for enrolling and consenting acutely ill inpatient and outpatient populations should consider the following questions: Where and how would this potential participant most likely learn about the study? How would the study team learn about this participant? What types of media and formats are most useful in the setting of acute illness? What are the barriers to identifying, reaching, and fully informing potential participants, their families, caregivers, or LARs about the proposed study?

Many sites expressed a preference for study materials that were concise. Designing participant-facing materials to optimize the amount of information provided would improve the enrollment process. Hospitalized patients with COVID-19 are often weak and need help performing even simple tasks; sites found the study overview videos somewhat more helpful for inpatient than for outpatient studies. The GPP Team developed audio flipbooks to make flipbooks more accessible and more cost-effective than videos. Audio flipbooks were made by adding a voice-over to the PowerPoint flipbook. When the GPP Team learned it was difficult for ill patients to advance the slides, they developed video flipbooks, which did not require manual slide advancement. Audio and video flipbooks were both rated more highly for inpatient studies.

For outpatient studies, recruitment efforts were focused on outreach to the public and to primary healthcare providers, testing and diagnostic centers, and pharmacies as the less-ill outpatient population was more likely to actively seek trial participation. Sites rated the CombatCOVID web pages as slightly more helpful for the outpatient OTAC study than for the ACTIV-3 and ACTIV-3b inpatient studies. For inpatient studies, efforts were focused on hospital clinicians, emergency departments, and review of information from health records. While it is unlikely that hospitalized patients with COVID-19 would search the internet for relevant clinical trials, site staff often sent hyperlinks to study-specific web pages for inpatient studies to families, caregivers, or LARs so they could read about the study, watch the study overview video and video flipbook, and download the flipbook.

Graphics can be an acceptable way to depict population diversity across many countries. They may be more acceptable to some ECs/IRBs and sites than photographs of people not considered representative of the local population. Providing materials in the languages of eligible populations is essential. Even when an expert translation service is used, site staff who know the local language and culture should review translations to ensure that the materials accurately reflect the local dialect and are appropriate for the population.

For some sites, high-quality color materials and high-technology tools may be preferable. For some low-resourced settings, black-and-white materials and low-technology tools may be more acceptable.

### Soliciting input from a wide range of stakeholders is imperative

Stakeholders include anyone who may impact or be impacted by the study. Members of the GPP Team and other stakeholders shared their experiences about recruitment challenges at the sites, made suggestions for and participated in the prioritization of study-specific materials and activities to aid study recruitment and implementation, and provided feedback on draft materials and plans for activities. Their input, intended to minimize the impact of differing health literacies in disparate populations [32], greatly improved the quality of the materials including their clarity, ease of understanding, simplicity, and acceptability in participating countries. Use of software was valuable in ensuring materials were accessible and at an appropriate reading level.

### Flexibility is crucial in effective GPP-EP implementation

Although some materials were originally intended to aid in a specific process, they were also used for other activities. For example, the flipbooks were developed to assist with informed consent; they were also used to aid recruitment by educating potential participants, their families, caregivers, medical providers, and the general public about the study as well as to inform and train site staff.

Flipbooks were printed on vinyl paper, which was cheaper than laminating paper, and collated in pocketless binders to allow for disinfection. However, vinyl paper and pocketless binders can be difficult to source, especially in resource-limited settings and during supply chain problems. Exploring sources and considering alternatives, such as laminating and using spiral binding, early in the process helps avoid delays in implementation. Some sites chose to print flipbooks and discard them after a single use.

Organizing flipbooks into separate modules allowed sites to remove slides based on study drug availability at the site and to remove modules to create a shorter flipbook. It also helped to save time and costs as slides that were unchanged between versions did not need to be printed and shipped again.

### Understanding IRB submission requirements improves efficiency and cost-effectiveness

Since draft video scripts with proposed visuals were submitted to the cIRB for provisional approval, the cIRB did not request any edits to the final study overview videos, thus minimizing costs and approval timeframes. Conversely, the process for submitting final videos proved challenging since the files were too large to be uploaded into the cIRB’s system, resulting in delays as solutions were devised. In addition, for accessibility, the study overview videos and video flipbooks had closed captions, which the cIRB could not save in their system and review. Open captions had to be developed solely for cIRB review.

Recruitment materials included placeholders for customization with site-specific contact information. cIRB approval covered this customization by US sites, and site-specific approvals were not required unless additional edits were made.

### A wide range of options is necessary

Considering the resources available to sites and the participants, low-resourced sites had difficulty with access to electronics and preferred printed materials. Settings with unreliable internet had difficulty downloading large files but could send emails or text messages with hyperlinks to the videos for families and LARs who were often remote. Furthermore, many ICUs did not allow electronics to be moved into or between rooms.

## Conclusion

This paper highlights the importance of GPP and how developing participant-facing materials, through consultation with stakeholders, is supportive of clinical research activities and effectively helps to address the language, cultural, and sociopolitical barriers during a pandemic. The results of the evaluation survey will be used to maximize efforts and allocate resources for new and ongoing studies like STRIVE.

## Supporting information

Guerra-de-Blas et al. supplementary material Guerra-de-Blas et al. supplementary material

## Supplementary material

The supplementary material for this article can be found at https://doi.org/10.1017/cts.2024.485.
