# Supplementary material for: Practical application of good participatory practices for trials of emerging pathogens: Developing materials for use in ACTIV-3, -3b, and ACTIV-associated COVID-19 trials
Source: J Clin Transl Sci. 2024 Oct 15;8(1):e157. doi: 10.1017/cts.2024.485 (PMC11602519; doi:10.1017/cts.2024.485)
Supplement: Guerra-de-Blas et al. supplementary material [file S2059866124004850sup001.pdf]

SUPPLEMENTARY MATERIAL to PRACTICAL APPLICATION OF GOOD PARTICIPATORY PRACTICES FOR TRIALS OF EMERGING PATHOGENS: Developing materials for use in ACTIV-3, -3b and ACTIV-associated COVID-19 trials by Guerra-de-Blas et al

TABLE OF CONTENTS

Supplementary Figure 1. Letter to Sites. 2
Supplementary Figure 2. Glossary of Terms Recommended by the GPP Team. 4
Supplementary Figure 3. Posters/Flyers. 6
Supplementary Figure 4. Recruitment Flyers. 7
Supplementary Figure 5. Yard Signs and Social Media Messages. 8
Supplementary Figure 6. Social Media Messages and Images. 9
Supplementary Figure 7. Email Messages. 12
Supplementary Figure 8. Webpages on the CombatCOVID and, Subsequently, NIAID Websites. 13
Supplementary Figure 9. Recruitment Letters for Potential Participants. 18
Supplementary Figure 10. Flipbooks. 23
Supplementary Figure 11. Participant Brochure. 38
Supplementary Figure 12. Thank You Letters. 40
Supplementary Figure 13. Letter for Participants to Share with mRNA COVID-19 Vaccine Providers.................... 42
Supplementary Figure 14. Proof of Recovery Letter and mRNA COVID-19 Vaccination Card. 45
Supplementary Figure 15. Protocol Quick Reference Guide. 46
Supplementary Figure 16. Study Summary Presentation. 48
Supplementary Figure 17. FAQ: Hyperimmune IVIG in OTAC. 52
Supplementary Figure 18. Dear Colleague Letter. 56
Supplementary Figure 19. Site Survey Questionnaire. 57

Note: Participant facing materials were collectively translated into 35 languages. Highlighted sections in the materials below provided information for the US cIRB and for sites to customize as appropriate.

Supplementary Figure 1. Letter to Sites. Letter sent from the Therapeutics Lead for the US Government Operation Warp Speed (OWS) and the Director of the National Institutes of Health (NIH) to the research leaderships at sites conducting ACTIV-3 and the ACTIV-associated DCR-sponsored study, Inpatient Treatment with Anti-Coronavirus Immunoglobulin (ITAC), asking them to prioritize the ACTIV studies.

DEPARTMENT OF HEALTH & HUMAN SERVICES Public Health Service

National Institutes of Health
Bethesda, Maryland 20892

[redacted]
Vice Dean for Research, [redacted]
Professor of Medicine, [redacted]

Re: [redacted]
Hospital Participation in ACTIV 3 Trial with Monoclonal Antibodies and Inpatient Treatment with Anti-Coronavirus Immunoglobulin (ITAC) by [redacted] **[*name of site principal investigator*]**

Dear Dr. [redacted]**:**

We are writing on behalf of the National Institutes of Health and the United States Government's Operation Warp Speed, dedicated to accelerating the development of COVID-19 therapeutics. Monoclonal antibodies and hyperimmune intravenous immunoglobulin (hIVIG) are among a number of very high-priority therapeutics under evaluation for the treatment of COVID-19. As you know, monoclonal antibodies have in general demonstrated an acceptable safety profile and promising in vitro activity. They are being studied in a trial called ACTIV-3. Highly concentrated antibodies obtained from multiple COVID-19 survivors have the potential to neutralize SARS-CoV-2 and are being studied in a trial called ITAC. These well-powered and rigorously designed randomized trials, in combination with the standard of care remdesivir, will hopefully determine their efficacy, understand dose-response relationships, and inform treatment guidelines.

Rapid implementation and completion of these trials represent a very high priority for NIH and Operation Warp Speed. As a potential clinical trial site, we ask for your maximum support of this trial to the degree possible including

- **Prioritization of these trials for rapid contractual and institutional approvals**

- **Provision of space and personnel to facilitate monoclonal antibody, hyperimmune globulin or placebo study infusions**
- **Dedication of rapid testing resources as available**

Please refer any questions regarding these clinical trials to the [redacted] Hospital Investigator, [redacted], the Protocol Chair for ITAC, [redacted], the Protocol Chair for ACTIV-3, [redacted] or the leader of the INSIGHT Washington International Coordinating Center, [redacted].

Thank you in advance for your willingness to prioritize these important efforts.

[redacted]

Janet Woodcock, MD
Operation Warp Speed

[redacted]

Francis S. Collins, MD, PhD
Director, National Institutes of Health

P.S. Other critical trials are testing the safety and efficacy of the Regeneron cocktail of monoclonal antibodies REGN-COV2 in outpatients and inpatients. This inpatient trial is also enrolling at [redacted] Hospital, please also consider it a high priority.

Supplementary Figure 2. Glossary of Terms Recommended by the GPP Team.

Instead of	Use
1:1 randomization	**Equal chance; 50:50 chance; 50% chance of receiving study drug**
2:1 randomization	**2:1 chance; 67% chance of receiving study drug**
Active drug or experimental drug	**Investigational drug or investigational study drug**: Used to refer to active study drug
Adverse events	**Possible side effects**
Ailment	**Disease or illness**
Anorexia	**Decreased or lower appetite**
Antibodies (without explaining them)	**Your immune system makes antibodies to fight germs/the virus.**
Arthralgia	**Joint ache/pain**
Blinded	**You and the study staff will not know if you are getting a study drug or placebo.**
Clinical manifestations, symptomatology	**Symptoms**
Clinical trials evaluate the safety and efficacy of experimental drugs	**Studies are needed to find out if new drugs are safe and help people get better faster.**
Coded specimens	**Your samples and study information will be coded. Your name is never used.**
Contraception (without explaining it)	**Birth control (contraception)**
Data	**Information**
Deep vein thrombosis	**Blood clot in a large vein (DVT, deep vein thrombosis)**
Discharged	**Leave the hospital**
Dyspnea	**Breathing problems; difficulty breathing; shortness of breath**
Edema	**Swelling of**
Efficacious; effective	**Helps people get better sooner/faster**
Eligibility	**To see if you qualify to be in the study**
Enroll	**Join the study**
Ethics committee	**Group or committee that protects your rights as a participant in the study**
Experimental	**Investigational: Use "investigational drug" which means it has not been proven to work, or "study drug" when describing investigational product.** Some people associate "experimental" with being experimented on like a guinea pig. IRB may have requirements on how investigational products are described.
Genetic testing (without explaining it)	**Testing of your genes**
Hospitalized	**In (the) hospital**
Immunization	**Vaccine dose; vaccination**
Infusion	**Given through a plastic tube into one of your veins**
IV or intravenous (alone) without explaining the term	**Intravenously (or IV through a plastic tube into one of your veins)**

Instead of	Use
Monoclonal antibody (without explaining it)	**When the virus that causes COVID-19 (SARS-CoV-2) enters your body, your immune system makes antibodies to fight it. Monoclonal antibodies can be made in a lab to act like natural antibodies.**
Myalgia	**Muscle aches**
Nasal congestion	**Stuffy nose**
Nasal swab	**Nose swab**
Nausea	**Feel sick**
Pathogen	**Germ**
Personal Identifiable Information (PII)	**Name and personal details; personal information**
Phlebotomy	**Blood draw or take blood or take a blood sample**
Placebo (without defining it in lay terms)	**Liquid/salt water/tablet/pill/capsule that looks like the study drug but does not have any drug in it and should not make you feel better or worse.**
Pneumonia	**Lung infection**
Pregnant woman	**Pregnant person**
Pulmonary embolism (PE)	**Blood clot in your lung**
Random	**Decided by chance (like flipping a coin)**
Remdesivir (without explaining what it is)	**Remdesivir (a drug that may decrease COVID-19 virus levels and shorten the time people with COVID-19 have to stay in the hospital)**
Rhinorrhea	**Runny nose**
Rigors	**Shaking chills**
SARS-CoV-2 (without explaining it)	**SARS-CoV-2 or the virus that causes COVID-19**
Severe dyspnea	**Serious breathing problems**
Specimen analysis	**Testing of samples**
Specimen repository	**Place where samples are stored**
Specimens	**Samples**
Sponsor (without explaining it)	**The sponsor or group paying for the study**
Standard of care (without explaining it)	**Standard care; medications or treatments you would usually receive**
Subject	**Participant or study volunteer**
Tachycardia or rapid heart rhythm	**Fast heart rate**
Trial or experiment	**Study; clinical research; clinical research study**
Unblinded or blinded	**You and the study staff will/will not know which group you are in.**
Ventilator	**A machine to help you breathe; breathing machine**
Woman of child-bearing potential	**Person who is able to get pregnant**

Supplementary Figure 3. Posters/Flyers. Contained expanded information about the study. Some of the items included are study name, eligible population, aim of study, duration of study participation, standard of care statement, uniform resource locators (URLs) and quick response (QR) codes for the respective study-specific webpage, and customizable local site contact information. Approval was secured from the United States (US) central institutional review board (cIRB) to use the digital image in social media messages.

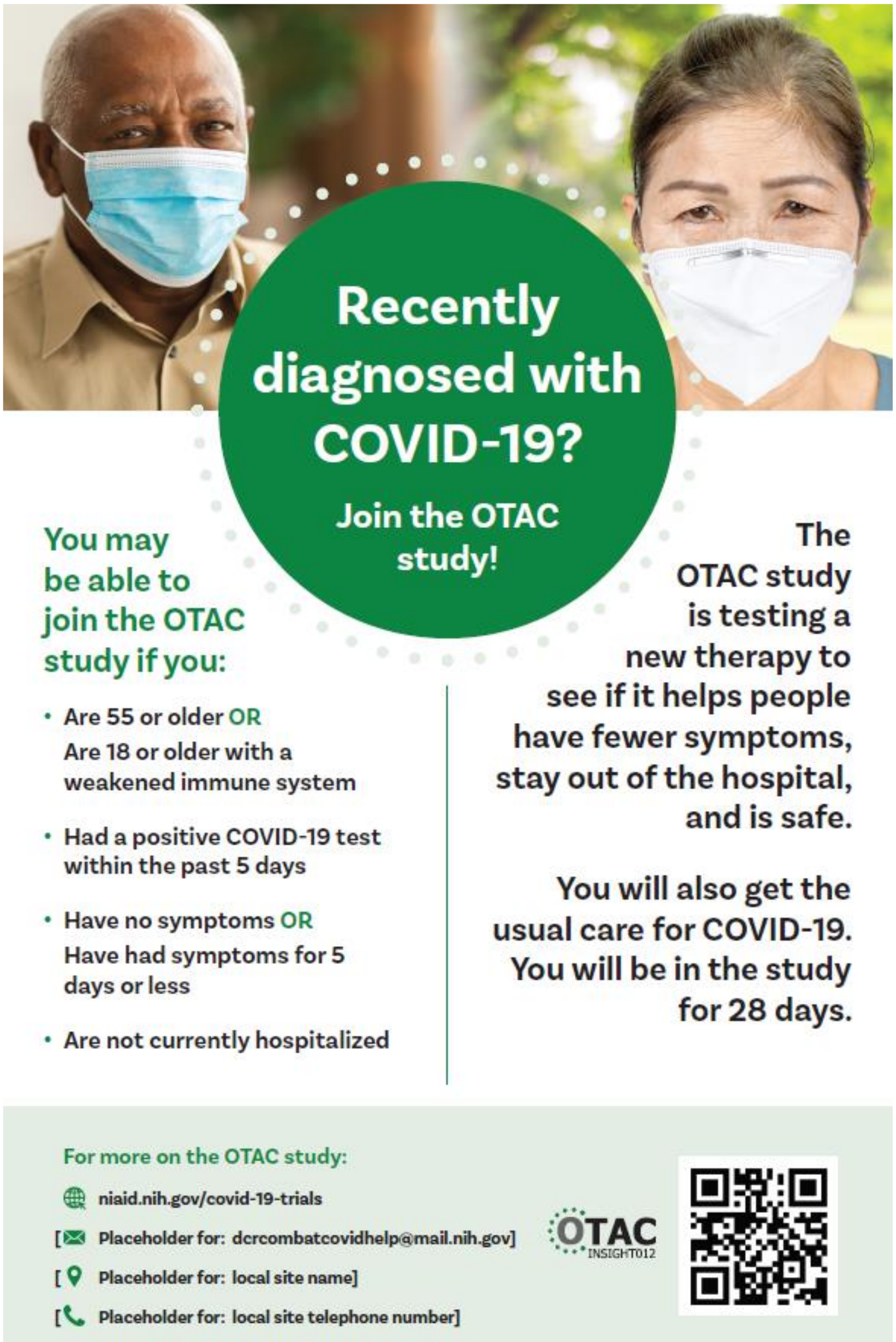

Supplementary Figure 4. Recruitment Flyers. Recruitment flyers were designed to be placed in clinics and facilities conducting testing for COVID-19 where potential participants, their families, caregivers, and legally authorized representatives could see them. They presented minimal information about a study testing an investigational treatment for people who have tested positive for COVID-19, the eligible population, the QR code for the study website, and space for local site contact information.

Tested Positive for COVID? Now What?

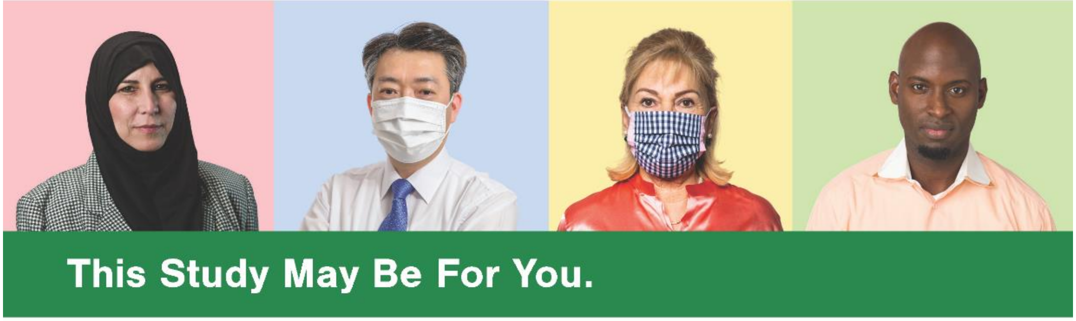

This Study May Be For You.

Researchers want to learn if an experimental drug can reduce the harmful effects of COVID-19 and help people stay out of the hospital.

You may be able to join the study if you:

☑	Are 55 or older<br>**OR**<br>Are 18 or older with a weakened immune system
☑	Have tested positive for COVID-19 within the past 5 days
☑	Are not having symptoms<br>**OR**<br>Have had symptoms for 5 days or less
☑	Are not currently hospitalized

There are other study requirements that the study team will explain to you to determine if you are able to join this study.

To learn more or to find out if you qualify for this study, contact:

[placeholder for study site information/google map]

Place holder for QR Code

NIH NIAID OTAC INSIGHT012 insight

Supplementary Figure 5. Yard Signs and Social Media Messages. Yard signs had similar content to flyers, designed to be placed outside the facilities conducting the trial and presenting minimal information: testing new treatment for COVID-19, URL and QR code for the study-specific webpage, customizable local site contact information. Approval was secured from the US cIRB to use the digital image in social media messages.

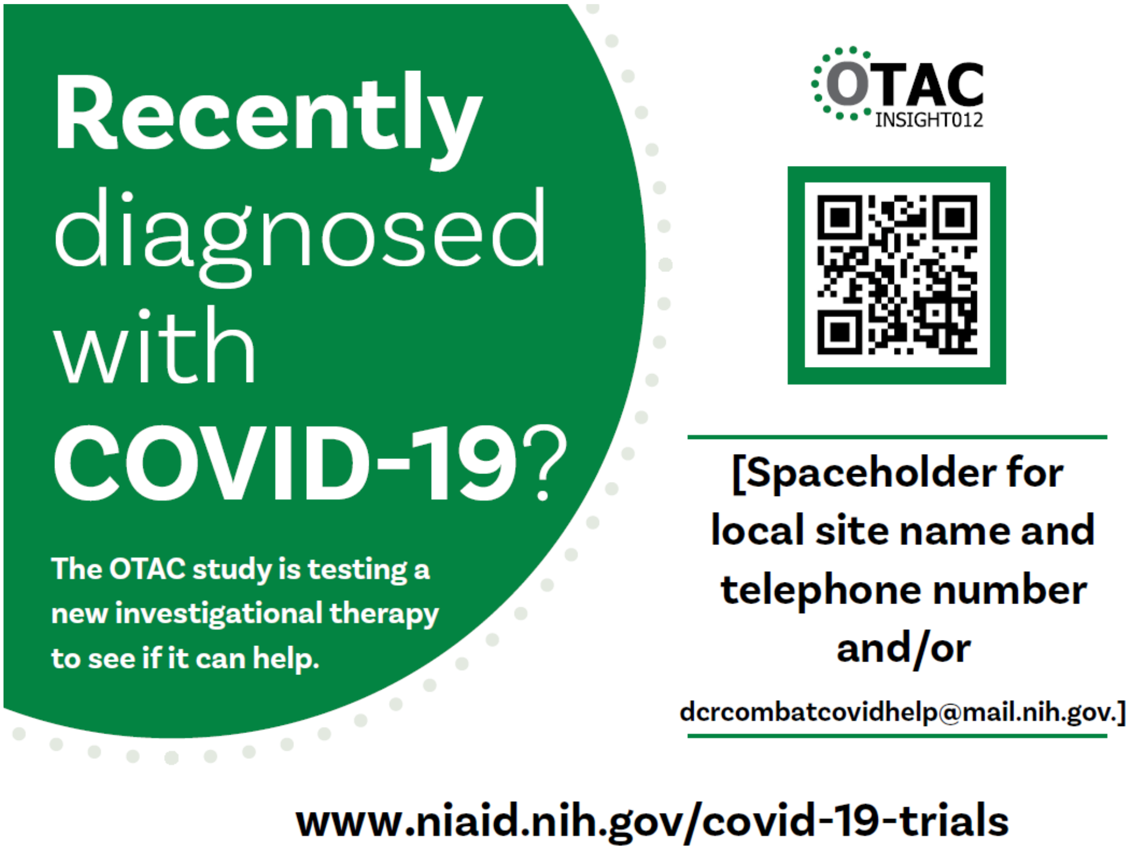

Supplementary Figure 6. Social Media Messages and Images. The social media messages were designed for research sites and NIAID to use in their various social media accounts. The messages included images of the posters and yard signs. There were also messages about the need for volunteers for a study testing anti-coronavirus hIVIG, eligibility criteria, URL for the study website, and customizable local site contact information. The digital images of posters and yard signs (Supplementary Figures 3 and 5) were also approved for use in social media messages.

insight

OTAC INSIGHT012

Using Social Media and E-mail for OTAC Recruitment

Sites are encouraged to use social media and e-mail to recruit participants into OTAC. **These communications must be IRB/EC approved.** [redacted] approval for use by US sites is noted in the descriptions of materials below. Non-US sites may wish to translate or modify these materials and obtain local EC approval.

NIAID Social Media Accounts

NIAID maintains the following social media accounts:

- Facebook: https://www.facebook.com/niaid.nih
- Twitter: @NIAIDNews
- Instagram: www.instagram.com/niaid (posted as 24-hour story)

These accounts have shared the following messages regarding OTAC; sites are encouraged to link to these messages on their own social media accounts:

- Facebook: https://www.facebook.com/niaid.nih/posts/pfbid051jhtFRgqUT1cBgiCgDqrabbDhAdxpidtrCvknomthTxC6cFQYDqzf2wS7hAwfnjl
- Twitter: https://twitter.com/NIAIDNews/status/1567236952319004673
- Instagram: https://www.instagram.com/stories/highlights/18187678654083649/

Social Media Messages for Use by Sites

The following messages in English have been approved by [redacted] and can be used on any social media accounts used by US sites. Note that the parts of the message highlighted in yellow should be customized with appropriate local contact information. The first message is suitable for Facebook; the second message is shorter and more suited for Twitter.

- Have you tested positive for COVID-19 within the last 5 days? [Insert OTAC site name] is looking for volunteers to participate in a study testing anti-coronavirus hyperimmune intravenous immunoglobulin (hIVIG) as an investigational treatment for COVID-19. To learn more, [Insert "visit [insert link to OTAC site webpage]" and/or "visit https://www.niaid.nih.gov/clinical-trials/outpatient-treatment-anti-coronavirus-immunoglobulin [link to NIAID OTAC webpage in English previously approved by [redacted]]" and/or "call [insert telephone number of OTAC site]" and/or "write [insert OTAC site email address]" and/or "write dcrcombatcovidhelp@mail.nih.gov"].

- Tested positive for COVID-19 in the last 5 days? [Insert local site name] is looking for #volunteers for a study testing #hIVIG as an investigational treatment for COVID-19. Learn more: [Insert "visit [insert link to OTAC site webpage]," and/or "visit https://www.niaid.nih.gov/clinical-trials/outpatient-treatment-anti-coronavirus-immunoglobulin," [link to NIAID OTAC webpage in English previously approved by [redacted]] and/or "call [insert telephone number of OTAC site]," and/or "write [insert OTAC site email address]," and/or "write dcrcombatcovidhelp@mail.nih.gov."].

Page 1 23 September 2022

Images for Use by Sites on Social Media

US SITES: The following images have been approved by Advarra for use in social media messages on all social media platforms. They may be downloaded from the INSIGHT website on the OTAC Study Promotion page. PDFs should be edited to add local contact information. Depending on the social media platform you are using, you may need to save the edited PDF as an image file. Choose whichever images work best on the social media platforms you use.

NON-US SITES: Sites that have already received EC approval to use the OTAC Overview (English) or the OTAC Overview (Spanish) as posters may be able to use them on social media depending on how the submission and approval specified means of distribution. PDFs downloaded from the INSIGHT website can be edited with translations and to add local site contact information.

- **Recruitment/Eligibility Graphic**
  This PDF contains a pair of images that must be used together.

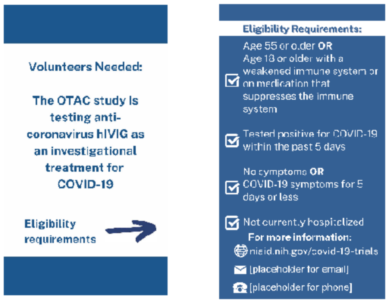

- **OTAC Overview (English)**

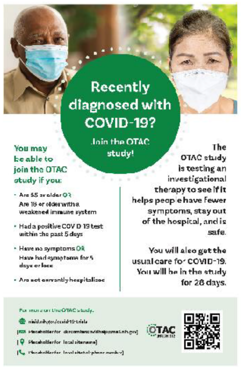

- **OTAC Overview (Spanish)**

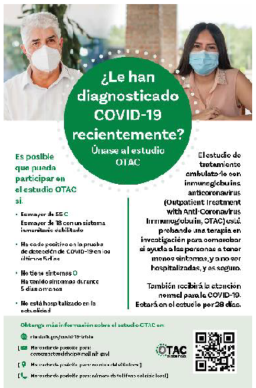

- **OTAC Sign (English)**

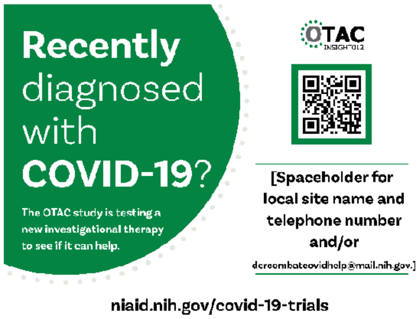

- **OTAC Sign (Spanish)**

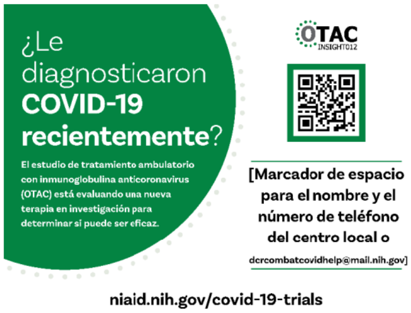

Supplementary Figure 7. Email Messages. The email messages contained information about the eligible trial population, statement about seeking study volunteers, study drug name (hIVIG), URL for NIAID study-specific website, and lists of site locations. The email messages were drafted for distribution by the research sites and for NIAID email distribution lists, including NIAID GovDelivery Email Listserv.

insight

OTAC INSIGHT012

E-mail Message for Use by Sites

The following e-mail in English has been approved by US cIRB and can be used by US sites on any relevant e-mail distribution list. Note that the parts of the message highlighted in yellow should be customized with appropriate local contact information.

Subject Line: Volunteers Needed - Have you tested positive for COVID-19 in the last 5 days?

We are seeking volunteers who have tested positive for COVID-19 within the last 5 days for a clinical trial testing an investigational COVID-19 treatment called anti-coronavirus hyperimmune intravenous immunoglobulin (hIVIG). Volunteers must meet the specific conditions shown below:

☑	Age 55 or older **OR**<br>Age 18 or older with a weakened immune system or on medication that suppresses the immune system
☑	Tested positive for COVID-19 within the past 5 days
☑	No symptoms **OR**<br>COVID-19 symptoms for 5 days or less
☑	Not currently hospitalized

For more information:

- [Insert "Visit: [insert link to OTAC site webpage]" and/or "Visit: https://www.niaid.nih.gov/clinical-trials/outpatient-treatment-anti-coronavirus-immunoglobulin [link to NIAID OTAC webpage in English previously approved by US cIRB]" and/or
- [Insert "Call: [insert telephone number of OTAC site]" and/or
- [Insert "Write: [insert OTAC site email address]" and/or "Write: dcrcombatcovidhelp@mail.nih.gov"].

Supplementary Figure 8. Webpages on the CombatCOVID and, Subsequently, NIAID Websites. The web pages contain detailed information about studies, including: study name; eligible population; aim of the study; schedule of study participation and activities; information about study drug (s), randomization, and placebo; URL for clinicaltrials.gov study webpage section with locations of sites and contact information; email address for more information; and study-specific flipbooks, overview videos, and video flipbooks.

Link example for a Spanish webpage: https://www.niaid.nih.gov/clinical-trials/tratamiento-ambulatorio-immunoglobulinas-anticoronaviral

Please note that the links may no longer be accessible; however, please find the script submitted to the cIRB below.

Outpatient Treatment With Anti-Coronavirus Immunoglobulin (OTAC)

https://www.niaid.nih.gov/clinical-trials/outpatient-treatment-anti-coronavirus-immunoglobulin

En Español

A research study testing an experimental COVID-19 treatment called anti-coronavirus hyperimmune intravenous immunoglobulin (hIVIG).

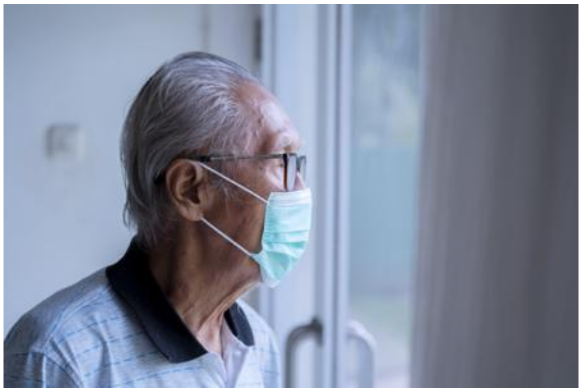

Credit: Adobe Stock

What Does the Study Involve?

The Outpatient Treatment with Anti-Coronavirus Immunoglobulin (OTAC) study is testing hIVIG as a treatment for COVID-19 (SARS-CoV-2).

What is hIVIG? hIVIG stands for “hyperimmune intravenous immunoglobulin.” hIVIG is made from blood plasma donated by healthy people who have recovered from COVID-19. Plasma is the liquid part of your blood.

The plasma is collected and purified to make hIVIG. It contains high levels of antibodies that fight the virus that causes COVID-19. Antibodies are natural proteins made by the body to fight or prevent infection.

The OTAC study is testing hIVIG to answer the following questions:

- Can this drug help people have fewer COVID-19 symptoms?
- Can the drug help people with COVID-19 stay out of the hospital?

- Is this drug safe for people with COVID-19 who may have a higher risk of getting very sick from their infection?

If you join this study, you will receive one dose of either hIVIG or a placebo (salt water which has no drug in it). Your chance of receiving the study drug will be 50%, which is just like flipping a coin. You will get the drug or placebo as a drip through a small plastic tube that is put into a vein in your arm. The study team will watch you while the dose is being given. They will also watch you after the dose is given. You will get the usual standard care for COVID-19 that you would receive even if you were not in a study. Your decision to join the study is purely up to you, and you can change your mind at any time.

[Placeholder for OTAC: Brief Study Overview video previously approved by XXX IRB]

How hIVIG Production and Administration Works

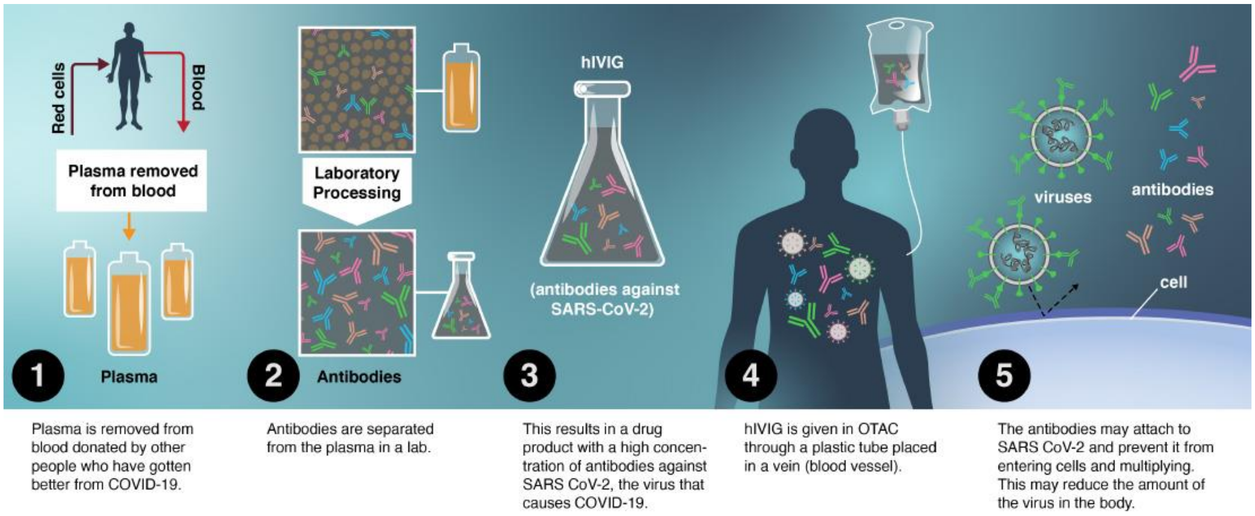

Credit: HHS

1. Plasma is removed from blood donated by other people who have gotten better from COVID-19.
2. Antibodies are separated from the plasma in a lab.
3. This results in a drug product with a high-concentration of antibodies against SARS CoV-2, the virus that causes COVID-19.
4. hIVIG is given in OTAC through a plastic tube placed in a vein (blood vessel).
5. The antibodies may attach to SARS CoV-2 and prevent it from entering cells and multiplying. This may reduce the amount of virus in the body.

Who Can Participate?

If you recently tested positive for COVID-19, this study may be for you. Clinical trials testing investigational COVID-19 treatments need people from diverse backgrounds. This will help researchers find treatments that work for more people. Each person who takes part in the OTAC study will meet specific conditions, including:

- **Age** – At least 55 years old **OR** at least 18 years old with a weakened immune system or on medication that suppresses the immune system
- **COVID-19 Status** – Not currently in the hospital, and tested positive for COVID-19 within the past 5 days
- **Symptoms** – No symptoms **OR** COVID-19 symptoms for no longer than 5 days

There are other requirements that the study team will explain to you. If you do not meet the requirements, or if there is not a study site near you, there may be other COVID-19 studies that you can participate in.

Where Is It Taking Place?

Visit ClincialTrials.gov [Link to ClinicalTrials.gov OTAC webpage with listing of OTAC site locations https://clinicaltrials.gov/ct2/show/NCT04910269?term=OTAC&cond=Covid19&draw=2&rank=1#contacts] to find an OTAC trial site near you.

Number of Visits Required

Your participation in this study will last for 28 days. You will check your temperature and your oxygen levels at home. Your study team will give you easy-to-use equipment at no cost to you to do this. Your study team will check on your health the day after the dose and then four more times after that. Most of the health checks can be done over the phone.

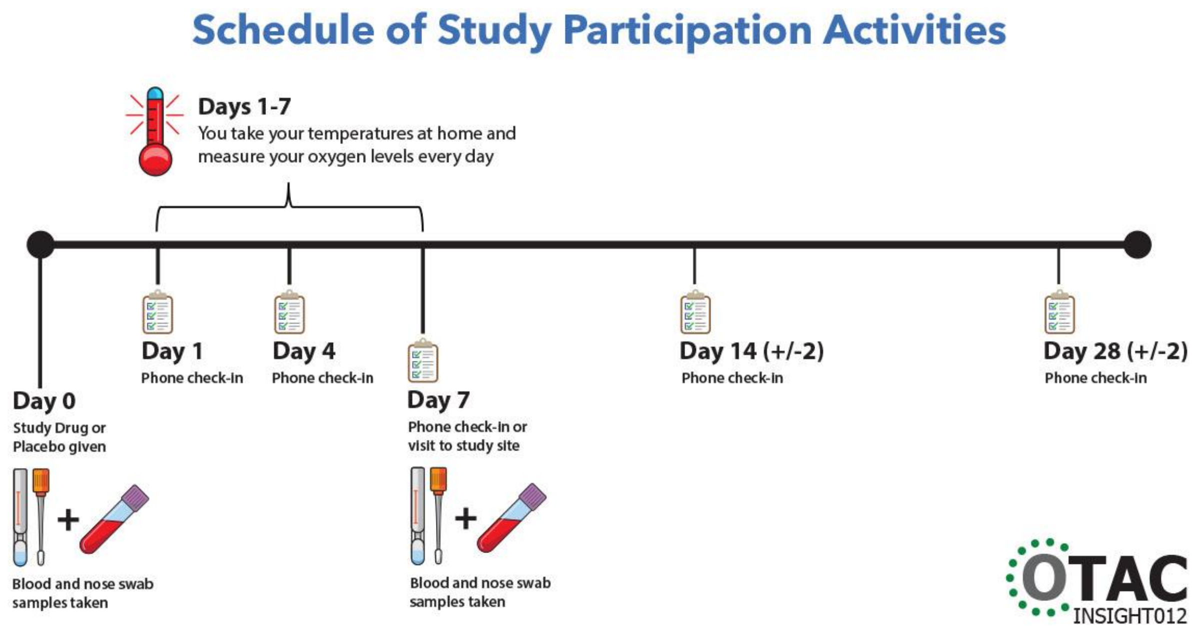

Credit: HHS

- **Day 0** – Study drug or placebo given; blood and nose swab samples taken
- **Days 1-7** – You take your temperatures at home and measure your oxygen levels every day
  - **Day 1** – Phone check-in

- Day 4 – Phone check-in
    - **Day 7** – Phone check-in or visit to study site; blood and nose swab samples taken
- **Day 14 (+/-2)** – Phone check-in
- **Day 28 (+/-2)** – Phone check-in

Steps to Participate

If you are interested in joining the OTAC clinical trial or you would like more information email: dcrcombatcovidhelp@mail.nih.gov

Resources

Study Information Video

[Placeholder for OTAC: Study Details Video (OTAC video Flipbook) approved by XXX IRB]

Study Information Downloadable Presentation

[Placeholder for downloadable OTAC Flipbook (PDF of PPT) approved by XXX IRB]

OTAC Study Information PDF

Answers to Other Questions You Might Have

What Are Clinical Research Studies?

A clinical research study helps experts find treatments that are safe and effective. It is important that these studies include people from diverse backgrounds. This helps researchers develop and test new treatments that will work for more people.

What Is a Placebo?

The placebo looks like the study medication but does not have any active ingredients in it. Researchers compare how people react to both placebos and medications to learn if the medication is safe and helps people recover more quickly. If you join this study, you will get the usual care for COVID-19.

Can I Change My Mind After I Join the Study?

Yes. Taking part in this clinical trial is voluntary; it is your choice. You can change your mind at any time and leave the study.

Participating in Research

Watch a series of short videos [link to https://www.hhs.gov/ohrp/education-and-outreach/about-research-participation/informational-videos/index.html] about participating in clinical trials. These videos are intended to help potential participants understand how research works, what questions they should consider asking, and things to think about when deciding whether or not to participate in a study.

Supplementary Figure 9. Recruitment Letters for Potential Participants. The GPP team created different versions: A two-page graphic format and one- and two-page text formats. They were letters to ACTIV-3 TICO participants about participating in the ACTIV-3 substudy VATICO.

A) Graphic Format

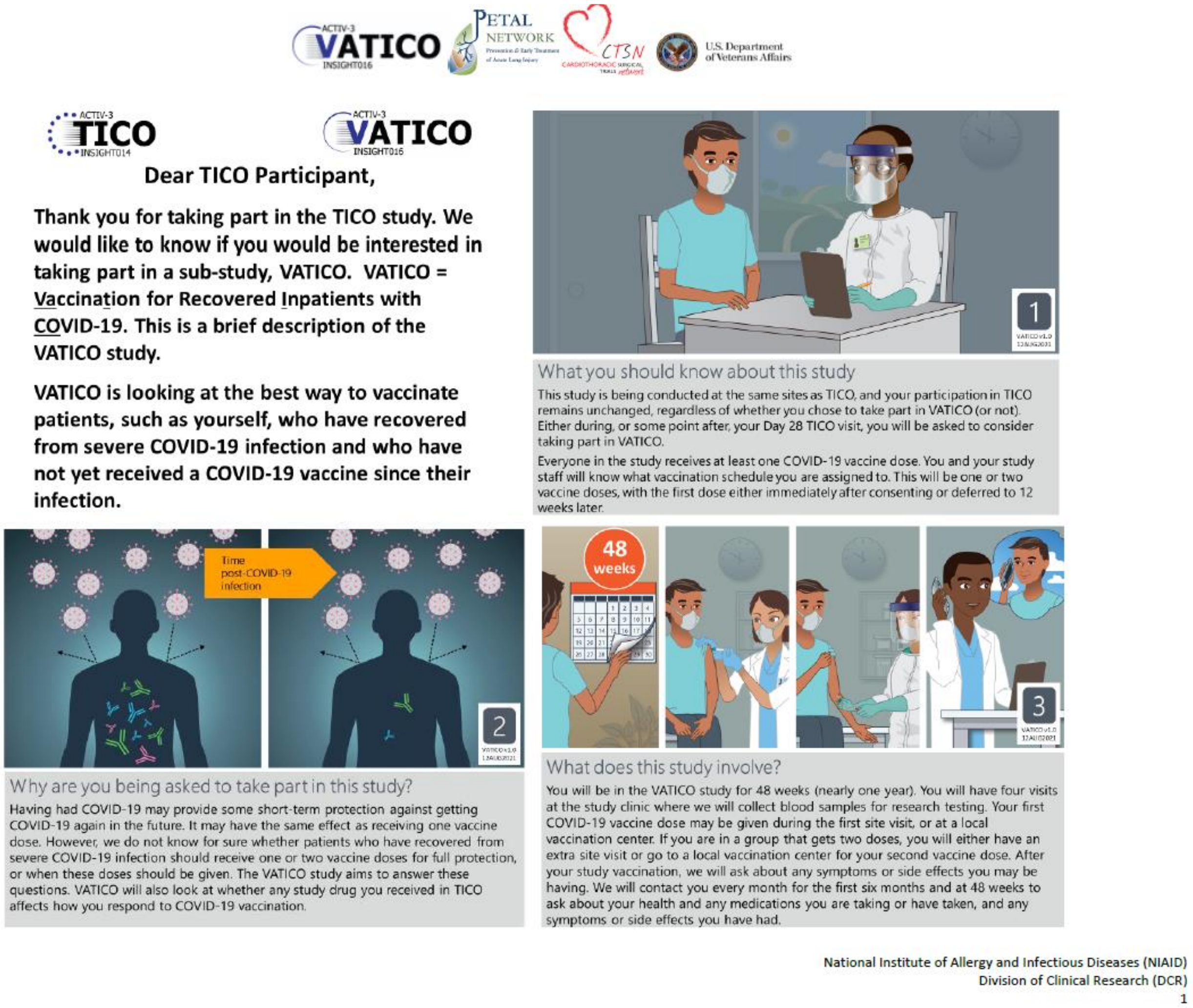

Dear TICO Participant,

Thank you for taking part in the TICO study. We would like to know if you would be interested in taking part in a sub-study, VATICO. VATICO = Vaccination for Recovered Inpatients with COVID-19. This is a brief description of the VATICO study.

VATICO is looking at the best way to vaccinate patients, such as yourself, who have recovered from severe COVID-19 infection and who have not yet received a COVID-19 vaccine since their infection.

What you should know about this study

This study is being conducted at the same sites as TICO, and your participation in TICO remains unchanged, regardless of whether you chose to take part in VATICO (or not). Either during, or some point after, your Day 28 TICO visit, you will be asked to consider taking part in VATICO.

Everyone in the study receives at least one COVID-19 vaccine dose. You and your study staff will know what vaccination schedule you are assigned to. This will be one or two vaccine doses, with the first dose either immediately after consenting or deferred to 12 weeks later.

Why are you being asked to take part in this study?

Having had COVID-19 may provide some short-term protection against getting COVID-19 again in the future. It may have the same effect as receiving one vaccine dose. However, we do not know for sure whether patients who have recovered from severe COVID-19 infection should receive one or two vaccine doses for full protection, or when these doses should be given. The VATICO study aims to answer these questions. VATICO will also look at whether any study drug you received in TICO affects how you respond to COVID-19 vaccination.

What does this study involve?

You will be in the VATICO study for 48 weeks (nearly one year). You will have four visits at the study clinic where we will collect blood samples for research testing. Your first COVID-19 vaccine dose may be given during the first site visit, or at a local vaccination center. If you are in a group that gets two doses, you will either have an extra site visit or go to a local vaccination center for your second vaccine dose. After your study vaccination, we will ask about any symptoms or side effects you may be having. We will contact you every month for the first six months and at 48 weeks to ask about your health and any medications you are taking or have taken, and any symptoms or side effects you have had.

National Institute of Allergy and Infectious Diseases (NIAID)
Division of Clinical Research (DCR)
1

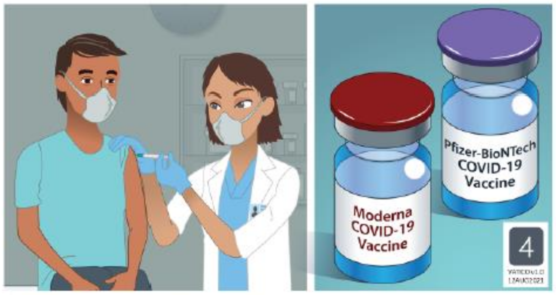

Which vaccines are being used in this study?

This study is using two COVID-19 vaccines made by Moderna and Pfizer-BioNTech. The US Food and Drug Administration (FDA) and the World Health Organization (WHO) recommend their use to protect against COVID-19 infection. Globally, hundreds of millions of people have already received these vaccines. They are safe and effective. You will get the vaccine as a shot in your upper arm. You will be observed for at least 15 minutes after the shot to ensure that if you have a rare allergic reaction, you get treated promptly.

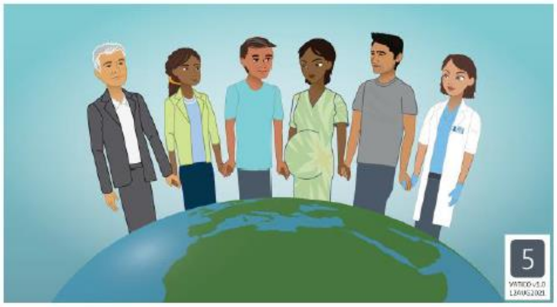

Are there benefits of being in this study?

We cannot promise you will have any direct benefit from being in this study. Getting vaccinated might help you. By taking part in this study, you will help doctors learn more about how best to vaccinate people who have recovered from severe COVID-19 infection.

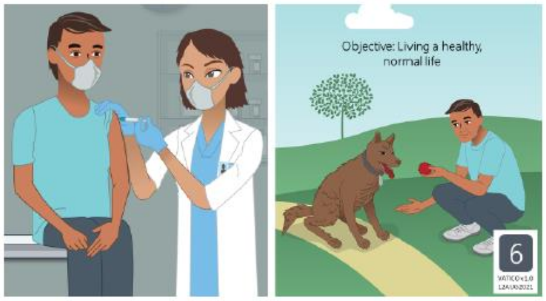

What if you do not join this study?

The U.S. Centers for Disease Control and Prevention (CDC), the Advisory Committee on Immunization Practices (ACIP), and the World Health Organization (WHO) recommend that people who have recovered from COVID-19 illness get vaccinated against COVID-19. If you decide not to join this study, you should discuss your vaccine options with your doctor.

Thank you for considering taking part in the VATICO study!

What happens next?

This letter is intended to see if you are interested in taking part in the VATICO study. Your site staff will provide you with detailed information about VATICO at your TICO Day 28 visit, as you may be eligible to participate at that time. Again, thank you so much for your continued participation in the TICO study, and we hope you consider participating in the VATICO study.

For further information on the study, please call the site coordinator:

Name: ___________________________

Phone: ____________________

B) One-Page Short Format

Dear TICO participant,
Thank you so much for your continued participation in the ACTIV-3 (TICO) trial. We are sharing information with you about an associated study, **VATICO (Vaccination for Recovered Inpatients with COVID-19)**, to see if you are interested in participating.

VATICO study
The VATICO study is looking at the optimal timing and number of doses of COVID-19 mRNA vaccines in people, such as yourself, who have recovered from severe COVID-19 infection. Everyone in VATICO will be scheduled to receive at least 1 dose of mRNA vaccine to help prevent another COVID-19 infection. We are studying whether you should receive 1 or 2 doses of the vaccines, and whether the doses are best given early, shortly after you've recovered from COVID-19, or deferred to a later time.

Vaccines in VATICO study
Moderna mRNA-1273 and Pfizer BNT 162b2 are the two licensed COVID-19 mRNA vaccines used for this trial. They have been given to millions of people worldwide and are considered safe. You will be fully informed about potential side effects of the vaccines before agreeing to participate in the VATICO study.

What is involved for you as a VATICO participant?
If you agree to participate, after hearing about the study and confirming that you are eligible, you will sign a consent form. The study involves 4-5 site visits, which include blood draws. These visits are at the beginning of the study; at weeks 12, 24, and 48 (end of study); and, depending on whether you get vaccinated at a local vaccination clinic, 1 or 2 visits to a vaccination clinic. You will also be contacted monthly by the site staff via phone for the first 6 months.

What vaccines will you receive and when?
You may be eligible to enroll into the VATICO study between Days 28 and 90 of the TICO study, depending on how long it takes you to recover from COVID-19. In VATICO, you will be randomized, by chance (like rolling dice), to 1 of 4 dosing groups to receive your first vaccine either early (within a week of when you enroll into VATICO) or deferred to 12 weeks after your VATICO enrollment date, and to receive either 1 or 2 doses of vaccine. The COVID-19 vaccine is given by a shot into the shoulder muscle. Second vaccine doses are given about 4 weeks after the first vaccine dose. Both you and your study staff will know your assigned treatment group.

What happens if I don't want to take part in the VATICO study?
It is still recommended you get vaccinated against COVID-19 to prevent future infections, unless you have a medical condition that prevents you from receiving a vaccine. Your decision on whether to take part in the VATICO study has no impact on your participation in the TICO study.

What happens next?
We hope you consider taking part in VATICO and if so, your site staff will provide you with detailed VATICO information at the time you may be eligible to participate. For further information on the study, please call the site coordinator:

Name: ____________________________________ Phone: ___________________________

National Institute of Allergy and Infectious Diseases (NIAID)
Division of Clinical Research (DCR)

C) Two-Page Long Format

Dear TICO participant,
Thank you so much for your participation in the ACTIV-3 (TICO) trial. Your continued involvement in this trial is greatly appreciated.

VATICO study
As a TICO participant, **you will be eligible to participate in another trial called VATICO**. VATICO stands for **Vaccination for Recovered Inpatients with COVID-19**. The same sites that conducted ACTIV-3 (TICO), the study you joined in the hospital, are conducting the VATICO study. VATICO is looking at the optimal timing and number of doses of COVID-19 mRNA vaccines in people, such as yourself, who have recovered from severe COVID-19 infection. We are sharing information about this study to see if you are interested in participating.

Vaccines in VATICO study
Moderna mRNA-1273 and Pfizer BNT 162b2 are the two licensed COVID-19 vaccines used for this study. You will be fully informed about potential side effects of the vaccines before agreeing to participate in the VATICO study.

What is involved for you as a VATICO participant?
If you agree to participate, after hearing about the study and confirming that you are eligible, you will sign a consent form agreeing to be in the study. The VATICO study includes 4-5 site visits for you: 1 visit at the beginning of the study; visits at weeks 12, 24, and 48; and, depending on whether you get vaccinated at a local vaccination clinic, 1 or 2 visits to a vaccination clinic. The 4 site visits involve blood draws to test how your immune system is responding to the COVID-19 infection, treatment, and vaccination. You will be contacted monthly by the site staff via phone for the first 6 months. You will also be seen at the end of the study, at week 48, to check how you are doing.

Basic information on VATICO
You will be eligible to join the VATICO study between Days 28 and 90 of the TICO study, depending on how long it takes you to recover from COVID-19. In VATICO, you will be assigned by chance (like rolling dice), to 1 of 4 dosing groups to receive your first vaccine either early (within a week of when you enroll into VATICO) or deferred to 12 weeks after your VATICO enrollment date, and to receive either 1 or 2 doses of vaccine. The COVID-19 vaccine is given by a shot into the shoulder muscle. If you are assigned to receive a second vaccine dose, this will be given about 4 weeks after the first vaccine dose.

This is an open label study, so both you and your study staff will know your assigned vaccine group. Please note, everyone in this study will be scheduled to get at least 1 dose of mRNA vaccine to help prevent another COVID-19 infection. We are studying whether you should receive 1 or 2 doses of the vaccine, and whether the doses are best given early or deferred.

Randomization Groups

Group	1st Dose	2nd Dose
1	YES<br>Within 2 weeks from now	NO
2	YES<br>Within 2 weeks from now	YES<br>About 4 weeks after 1st dose
3	YES<br>About 12 weeks from now	NO
4	YES<br>About 12 weeks from now	YES<br>About 4 weeks after 1st dose

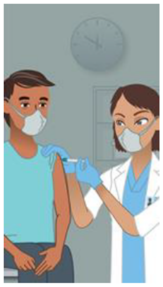

What happens if you don't want to take part in the VATICO study?
Regardless of whether you decide to take part in the VATICO study, it is recommended you receive a COVID-19 vaccination to prevent future infections, unless you have a medical condition that would prevent you from receiving a vaccine. Your decision on whether to take part in the VATICO study has no impact on your participation in the TICO study.

Safety of the vaccines in this study
The United States (US) Food and Drug Administration (FDA) and the World Health Organization (WHO) recommend the use of these vaccines to prevent COVID-19 infection. Globally, hundreds of millions of adults have already received these vaccines. They are safe and effective.

Some people have had side effects from getting a COVID-19 vaccine. Common side effects include pain, swelling (hardness), and redness in the arm of the shot, as well as side effects of tenderness and swelling of the lymph nodes near the area of the shot. Other common general side effects of the vaccines may include tiredness, headache, muscle aches, joint pain, chills, fever, nausea, and vomiting. These side effects usually happen 12-24 hours after the vaccination. They typically go away on their own in a few days. You may not have any side effects.

What if I have questions about COVID-19 vaccines in general?
Like many other people, you may have questions or concerns about the COVID-19 vaccine in general. Please discuss these with your study team.

What happens next?
This letter is intended to see if you are interested in taking part in the VATICO study. Your site staff will provide you with detailed information about VATICO at your TICO Day 28 visit, as you may be eligible to participate at that time. Again, thank you so much for your continued participation in the TICO study, and we hope you consider participating in the VATICO study.

For further information on the study, please call the site coordinator:

Name: ______________________________ Phone: ______________________

Supplementary Figure 10. Flipbooks. These contained detailed study information in simple terms. Some items were: study name, study aims, standard of care statement, general information about clinical trials, need for informed consent, study drugs, potential risk and benefits, side effects, placebo, randomization, schedule of study participation and activities, pregnancy and contraception requirements, protection of privacy, use of samples and data collected, funding source, URLs for study-specific webpages. The organization of flipbooks evolved as they were amended to add or remove investigational products and to reflect the master protocol, protocol appendices, and protocol amendments. The aims of the reorganization were to allow unaffected modules to remain unchanged, to optimize slide numbering so that addition or removal of slides affected only the numbering in that module, and to provide optional modules (e.g., Modules IV. and V.) that could be removed for a shorter flipbook. Ultimately, flipbooks were organized into modules as in the sample of images from flipbooks shown below:

I. Study Overview
   1. What you should know about this study
   2. What is a clinical research study?
   3. What is this study about?
   4. Will you get a study drug or placebo?
   5. What happens if you agree to be in this study?
   6. What does this study involve?
   7. Will you receive other care for COVID-19?
   8. What else will happen during this study?
   9. What do you need to know about sex, pregnancy, and breastfeeding during this study?
   10. Are there benefits of being in this study?

II. Study Drugs Being Given
   1. What drugs are being studied?
   2. What kind of study drug is [Study Drug 1]?
   3. What can you expect if you are placed in the [Study Drug 1] group?
   4. What could be the side effects from [Study Drug 1]?
   5. What kind of study drug is [Study Drug 2]?
   6. What can you expect if you are placed in the [Study Drug 2] group?
   7. What could be the side effects from [Study Drug 2]?

III. Placebo and Randomization
   1. What is a placebo?
   2. What is your chance of getting a study drug?
   3. Are there other risks or discomforts related to this study?

IV. Other Information about the Study
   1. What will happen to your samples and personal information?
   2. How will your privacy be protected?
   3. What else should you know about study participation?

V. Consent for Genetic Testing
   1. Additional consent for genetic testing on stored specimens

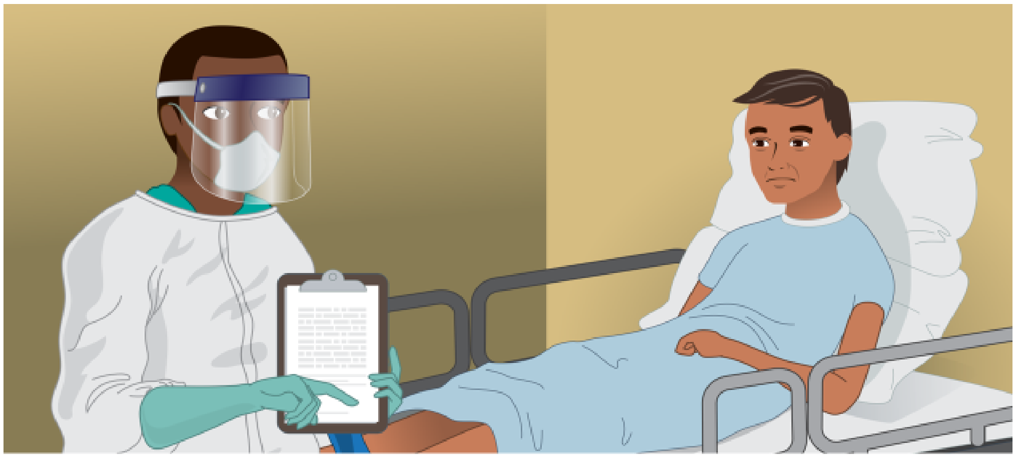

What you should know about this study

- We are talking to you about this clinical research study because you are in the hospital with COVID-19. You may wish to join this study.
- Please read this information carefully or have someone you trust read and explain it to you. Take as much time as you need. You can also talk to your family and friends about the study. Ask the study team to explain any words or information that you do not understand.
- You are a volunteer. If you join the study, you can change your mind later. You can decide not to take part, or you can leave the study at any time.
- There will be no loss of benefits if you decide not to join or later decide to leave the study. The medical care you are getting will not be affected.

1

ACTIV 3 v5
22JUN2021

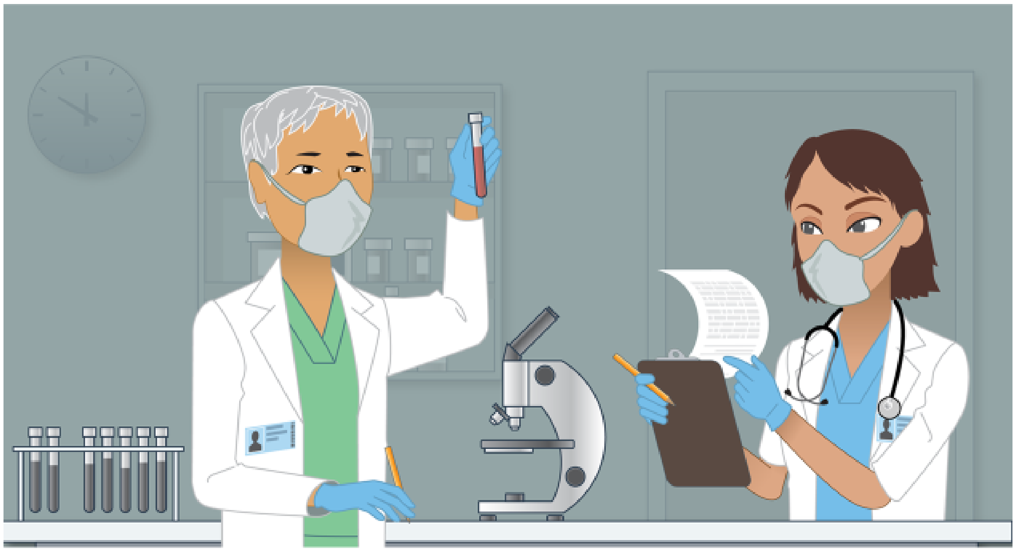

What is a clinical research study?

A clinical research study helps doctors find new ways to treat patients with a particular disease. One way to do this is by studying new drugs to see if they work and are safe. In a study, the drugs are "investigational," which means they have not been proven to help people with your disease. That is why studies are needed to find out if new drugs are safe and help people get better faster.

2

ACTIV 3 v5
22JUN2021

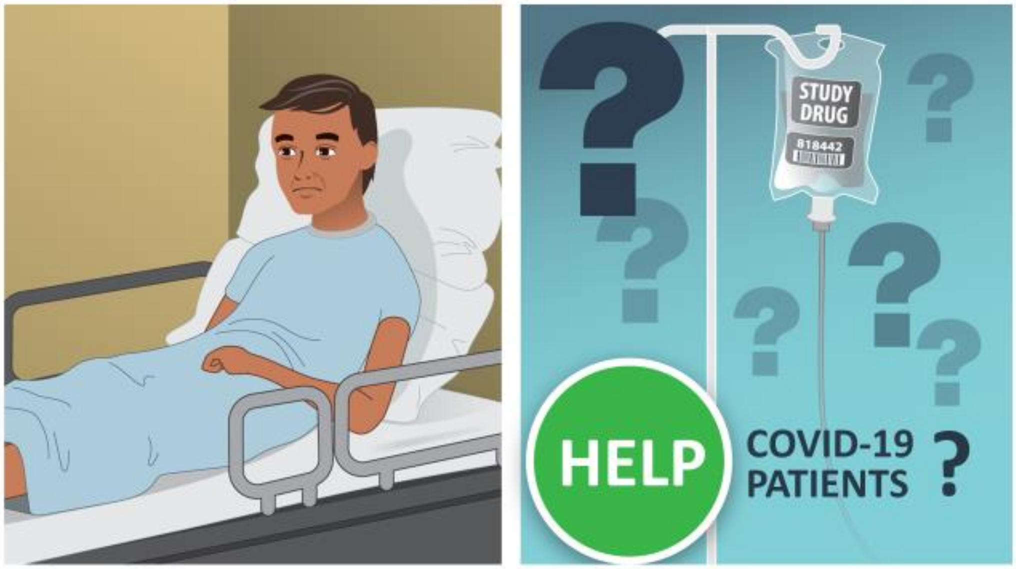

What is this study about?

We are looking for new treatments for patients in the hospital with COVID-19. We want to see if the study drugs are safe and will help people with COVID-19 get better and go home faster compared to people who do not get the study drug. People who do not receive the study drug will get a placebo

We expect to enroll about 1,000 people around the world for each study drug.

3

ACTIV 3 v5.1
17NOV2021

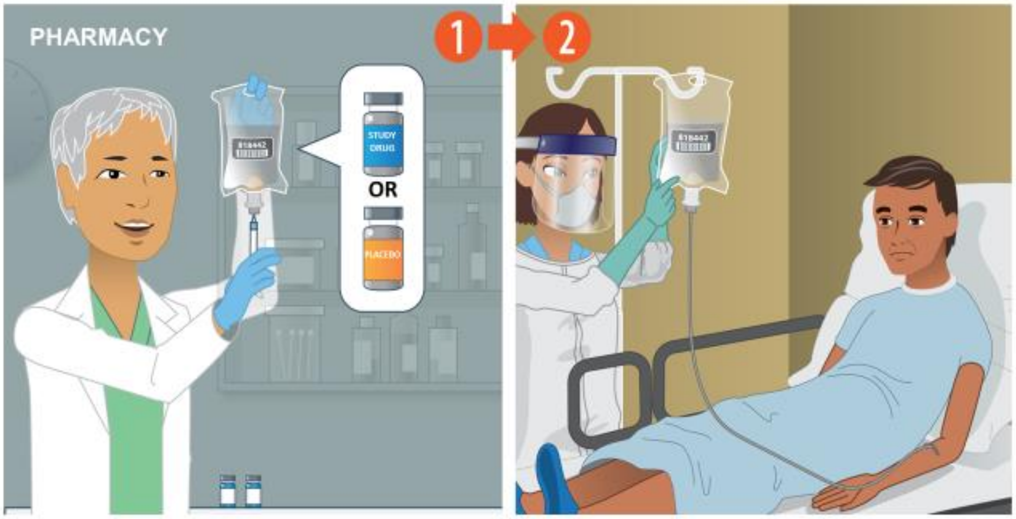

Will you get a study drug or placebo?

If you join the study, you will be randomly put into either a study drug group or the placebo group. People in the placebo group will not receive a study drug. They will get an infusion that has no drug in it (a placebo).

Your study group is decided by chance – like flipping a coin. You and the study staff will not know which study group you are in or if you are getting a study drug or the placebo.

4

ACTIV 3 v5
22JUN2021

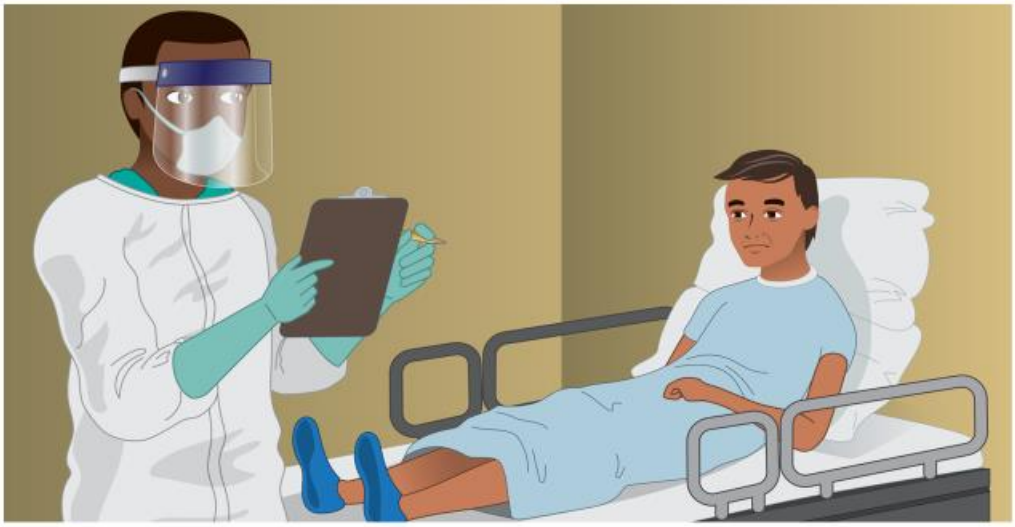

What happens if you agree to be in this study?

If you sign the consent form, it means you agree to be in the study.

After you sign the consent form, we will review your past and current health, give you a brief physical exam, collect a swab from your nose to test for the virus, and take blood for tests to check your health. We may do a pregnancy test if you are a woman who is able to have children. We will use this information to see if you qualify to be in this study and to monitor your health.

5

ACTIV 3 v5
22JUN2021

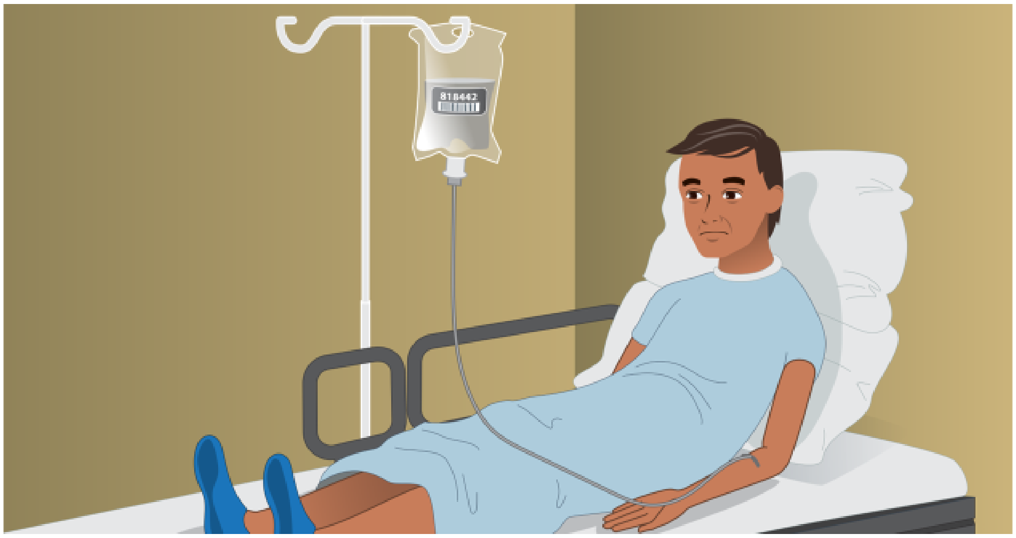

What does this study involve?

If you join the study, you will get an infusion of one of the study drugs or placebo. You may get an infusion that lasts for less than an hour or that goes for several days in a row. The infusion will be intravenous (IV or into one of your veins) while you are in the hospital. We will watch you closely for side effects during and after the infusion.

You will also be given other medications or treatments that you would usually receive in this hospital for your COVID-19 illness (standard care).

6

ACTIV 3 v5
22JUN2021

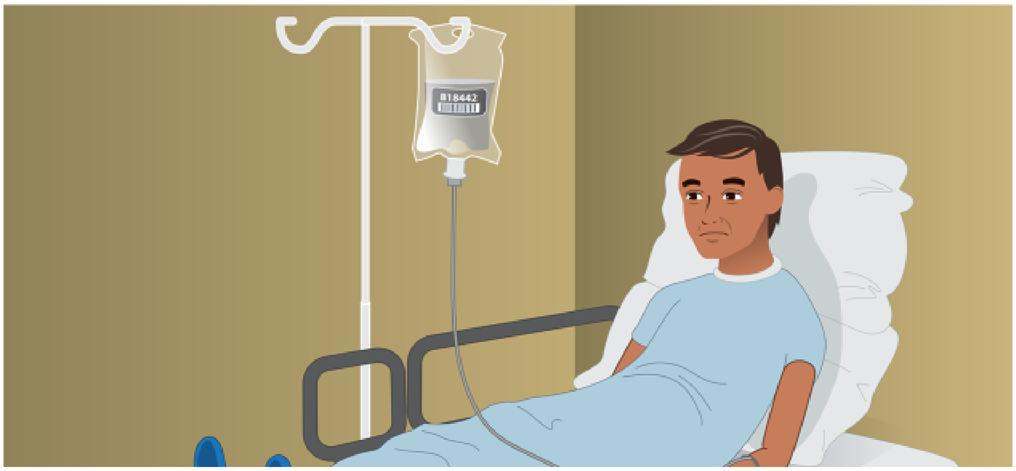

Will you receive other care for COVID-19?

People in the study will also receive standard care. In the United States, standard care includes a medicine approved for treating people with COVID-19 called remdesivir. It may decrease COVID-19 virus levels and shorten the time people with COVID-19 have to stay in the hospital. All people in this study will receive remdesivir as part of standard care.

Remdesivir is given as an infusion through a plastic tube into a vein in your arm each day for up to 10 days. Each remdesivir infusion lasts up to one hour. Standard care may also include steroids. Your doctor will advise you whether you should receive steroids.

7

ACTIV 3 v5.1
17NOV2021

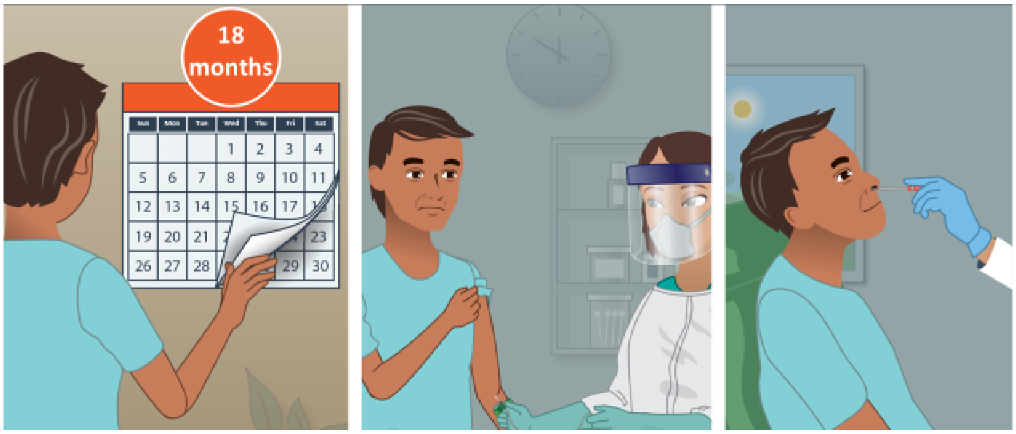

What else will happen during this study?

You will be in the study for a total of 18 months. Most of your study visits will be in the first 90 days. While you are in the hospital the study team will check your health every day during the first week.

After you leave the hospital, visits on Days 3, 5, 28 and 90 may take place in the study clinic or at your home. We will also call you up to 5 times in the first 90 days and at 6, 12, and 18 months to see how you are doing and ask if you have been in the hospital.

We may collect information from your medical records at the hospital as well as any other hospital or facility you may be admitted to, while you are in this study. At some study visits, we will also ask about medicines you have taken. We will collect a nose swab at the first visit and a blood sample at 6 visits.

8

ACTIV 3 v5.1
17NOV2021

Sex, Pregnancy, Breast Feeding, and Birth Control

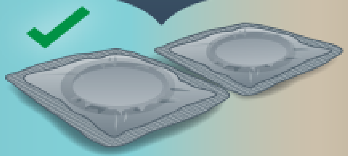

What do you need to know about sex, pregnancy, and breastfeeding during this study?

If you are pregnant or breastfeeding, you cannot join this study. If you or your partner can get pregnant and you join this study, you must agree not to have sex that could cause pregnancy. This means you should use an effective form of birth control (contraception) or stop having sex that could make you or your partner pregnant. If your partner is pregnant, you should stop having sex or use a condom. You should not donate sperm.

The study team will discuss acceptable forms of contraception with you and how long you will need to use them.

9

ACTIV 3 v5
22JUN2021

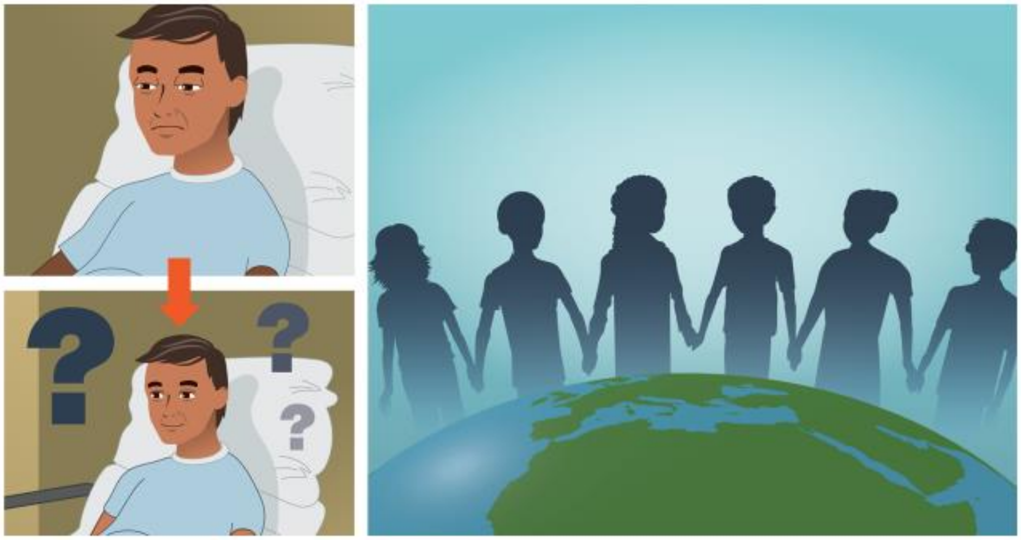

Are there benefits of being in this study?

If you get a study drug, it may help you get better, increase your chances of surviving, and go home faster. We do not know if a study drug will help. It is important to remember that some people in this study will get placebo and not a study drug.

By being in this study, you will help doctors learn more about how to treat people in the hospital with COVID-19. If the study drug is shown to be safe and effective, there may be a large health impact with many lives saved.

10

ACTIV 3 v5
22JUN2021

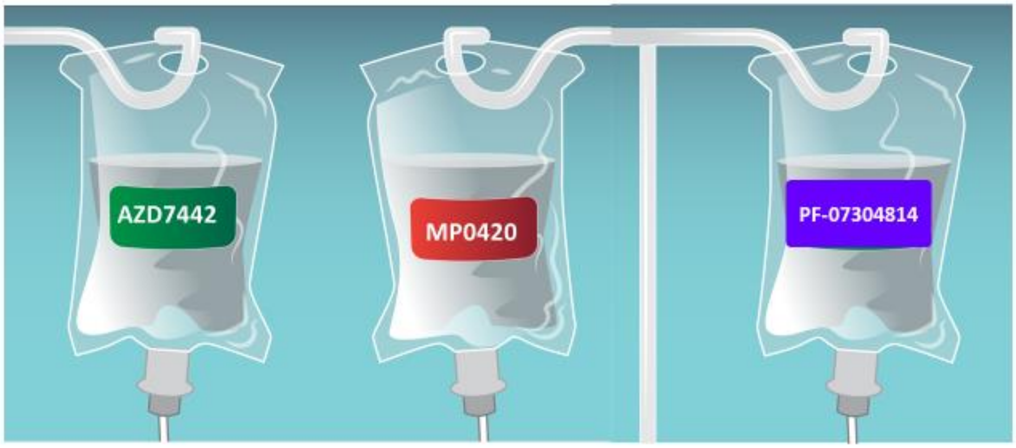

What drugs are being studied?

We are studying three different drugs.

1. AZD7442, which is made by a company called AstraZeneca.
2. MP0420, which is made by two companies, Molecular Partners and Novartis.
3. PF-07304814, which is made by a drug company called Pfizer.

Not all of the study medicines listed above may be available to you right now. If this is the case, the study team will tell you which ones are available.

1A

ACTIV 3 v3.3
27APR2021

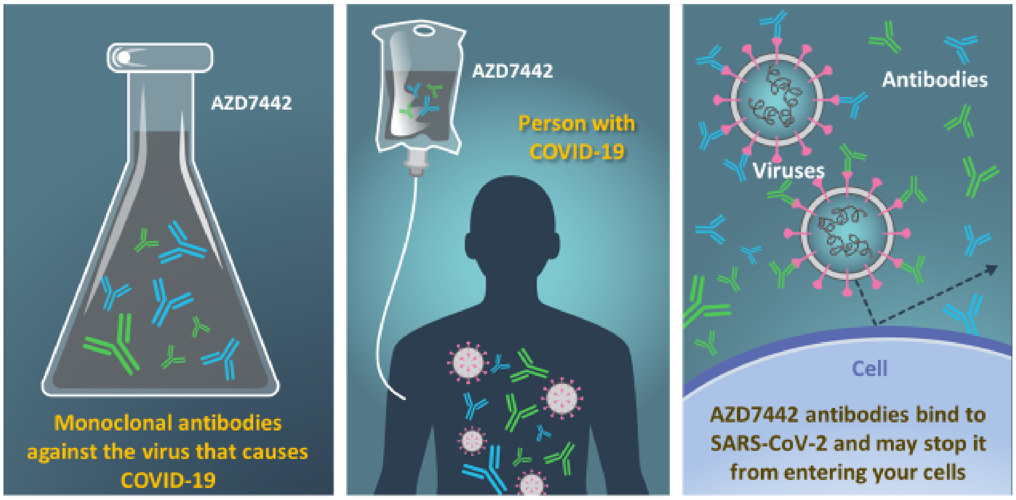

What kind of study drug is AZD7442?

AZD7442 is a study drug made of monoclonal antibodies (MAbs). When the virus that causes COVID-19 (SARS-CoV-2) enters your body, your immune system makes antibodies to fight it. Monoclonal antibodies can be made in a lab to act like natural antibodies.

AZD7442 has two MAbs that were identified from people who recovered from COVID-19 and made in a lab. Both MAbs try to block the virus that causes COVID-19. Each works on a different part of the virus. In lab tests, AZD7442 also worked against the variants (mutants or new versions) of the virus that were tested. We hope AZD7442 can help you get better faster from COVID-19, but we do not know if it will work in people.

2A

ACTIV 3 v5
22JUN2021

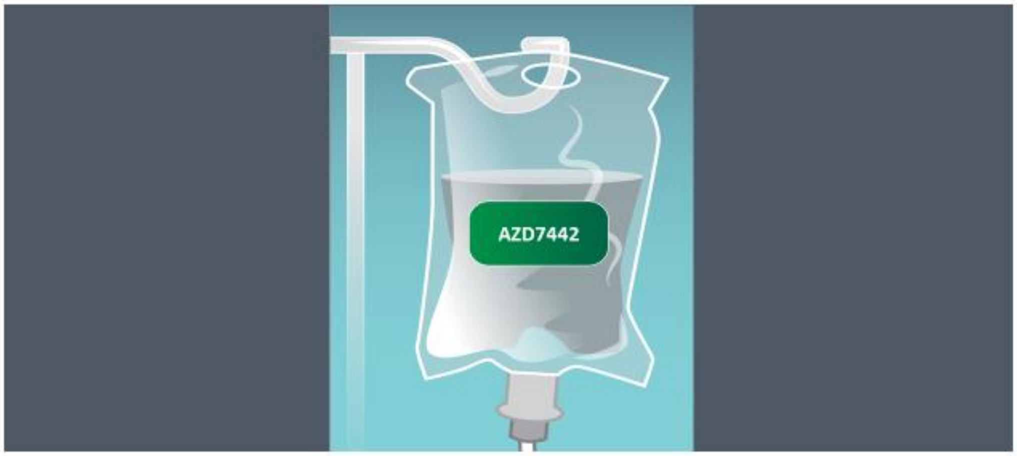

What can you expect if you are placed in the AZD7442 study group?

AZD7442 is given by intravenous (IV) infusion, through a plastic tube attached to a needle in your arm. The infusion will take about 30 minutes. We will watch over you closely during the infusion and for 2 hours after. We will treat you right away if you have a reaction.

If you are placed in the AZD7442 study group, we strongly advise you not to have sex that could make you or a partner pregnant for the full 18 months you are in the study. The study team will discuss acceptable forms of contraception with you.

3A

ACTIV 3 v5
22JUN2021

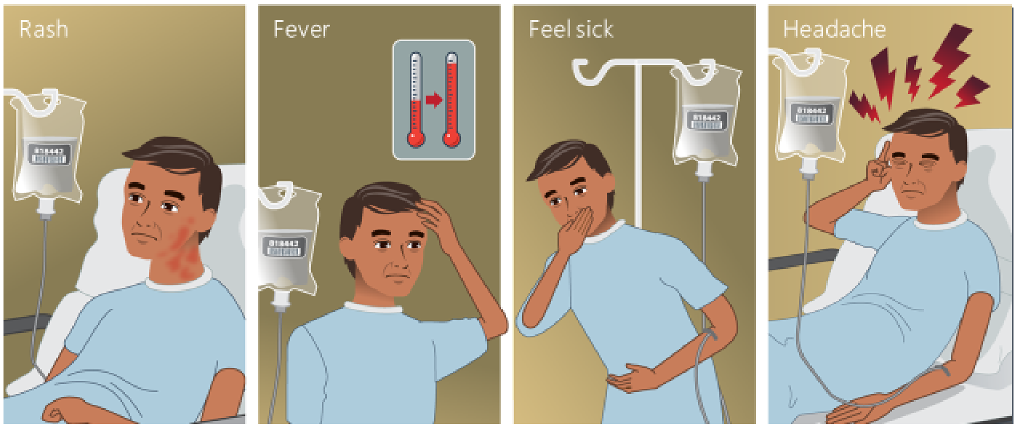

What could be the side effects from AZD7442?

Side effects from AZD7442 have occurred in other studies and can be severe and/or life threatening, but they are not common.

Side effects seen in people receiving monoclonal antibodies like AZD7442 include chills, itching, rash, headache, fever, muscle aches, feeling dizzy, a fast heart rate, difficulty breathing, and changes in some laboratory tests that measure blood cell counts.

Like all drugs, AZD7442 may also cause an allergic reaction. Allergic reactions are rare but can be serious. Allergic reactions may cause skin rash, itching, swelling of the face or other parts of the body, difficulty breathing, vomiting, diarrhea, a drop in your blood pressure, and chest tightness. Allergic reactions can be treated by slowing or stopping the infusion and giving medicines like Benadryl or other antihistamines and, if a reaction is severe, adrenaline.

4A

ACTIV 3 v5
22JUN2021

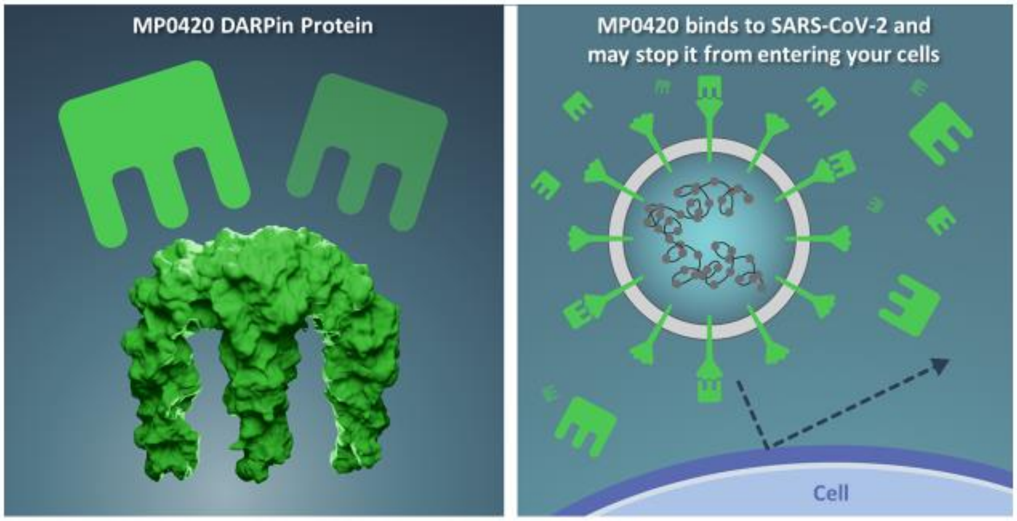

What kind of study drug is MP0420?

MP0420 is a protein called a DARPin that is made in a laboratory. It attaches to 3 different parts of the virus that causes COVID-19 (SARS-CoV-2). MP0420 works to try to keep SARS-CoV-2 out of your cells.

In lab tests, MP0420 also worked against the variants (mutants or new versions) of the virus that were tested. We hope MP0420 can help people with COVID-19 get better faster, but we do not know if it will—which is why we are doing the study.

5A
ACTIV 3 v5
22JUN2021

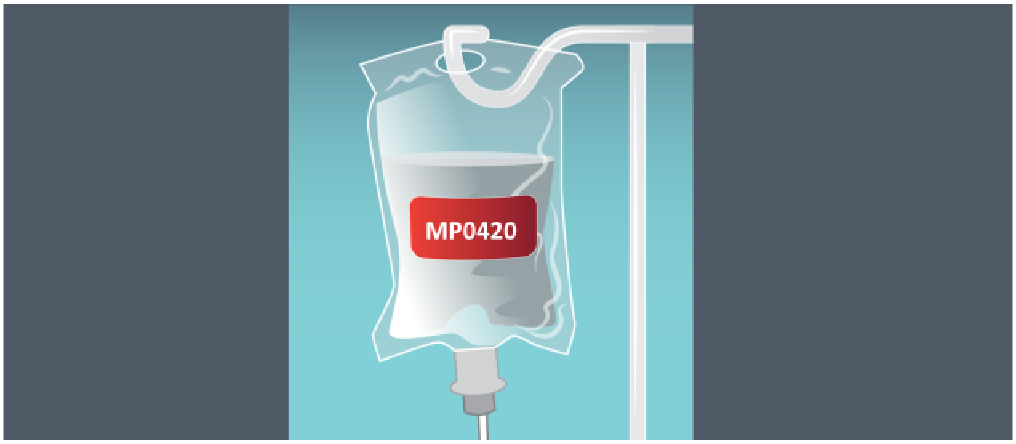

What can you expect if you are placed in the MP0420 study group?

MP0420 is given by intravenous (IV) infusion through a plastic tube attached to a needle in your arm. The infusion will take about an hour. We will watch you closely during the infusion and for 2 hours after. We will treat you right away if you have a reaction.

If you are placed in the MP0420 study group, we strongly advise you not to have sex that could make you or a partner pregnant for 11 weeks after you get the infusion. The study team will discuss acceptable forms of contraception with you.

6A
ACTIV 3 v5
22JUN2021

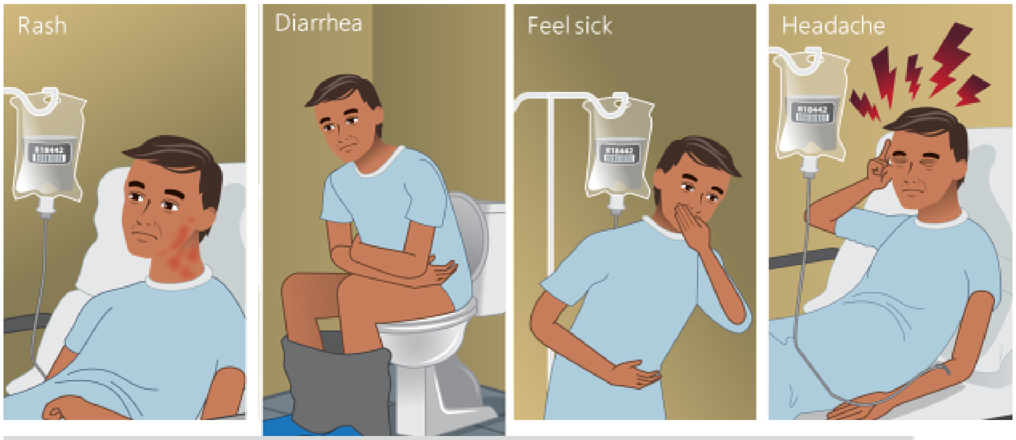

What could be the side effects from MP0420?

MP0420 was studied in 16 healthy people who did not have COVID-19. 12 of these people got MP0420 and 4 got a placebo (salt water). Of the 16 people, 3 had some mild side effects. One had a high liver test result and diarrhea. One had a headache. One person had skin redness on the arm where they had the infusion. These side effects all went away on their own on the same day as the infusion. We do not know whether these people got MP0420 or placebo.

MP0420 may cause an allergic reaction. Allergic reactions may cause skin rash, itching, swelling of the face or other parts of the body, difficulty breathing, vomiting, diarrhea, a drop in your blood pressure, and chest tightness.

7A
ACTIV 3 v3.3
27APR2021

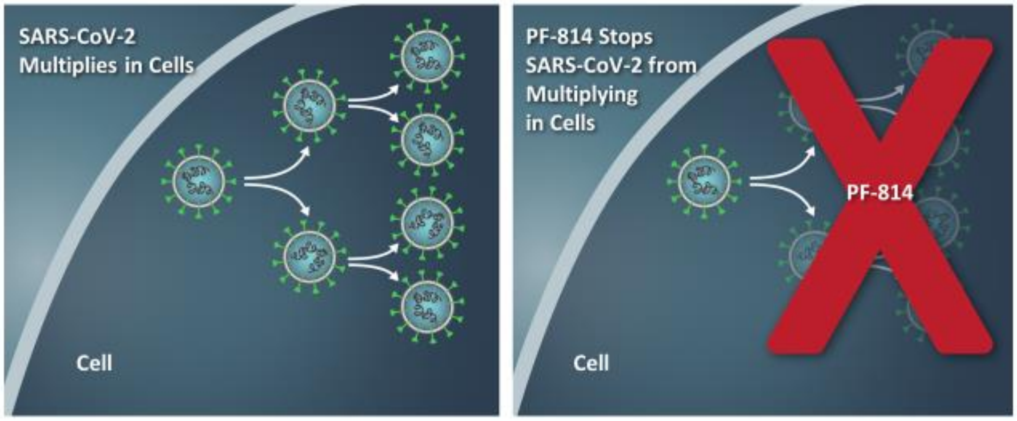

What kind of study drug is PF-07304814?

PF-07304814, also called PF-814, is a kind of drug called a protease inhibitor. It stops SARS-CoV-2, the virus that causes COVID-19, from multiplying. This means there could be fewer viruses for your body to fight, and you may get better faster from COVID-19.

You may not be able to be in the PF-814 study group if you have ever had a blood clot in a large vein (DVT, deep vein thrombosis,) or in your lung (PE, pulmonary embolism), if your liver is not working well, or if you are taking certain medications.

2A
ACTIV 3 v5.1
17NOV2021

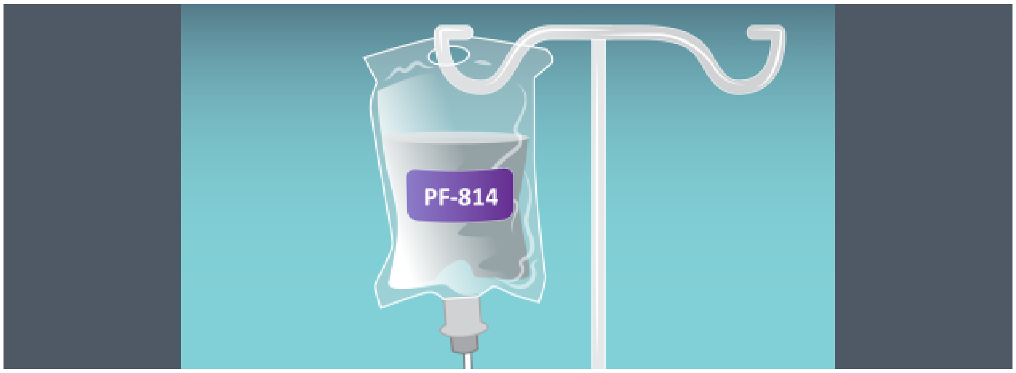

What can I expect if I am placed in the PF-814 study group?

PF-814 is given by intravenous (IV) infusion through a plastic tube into one of your veins. It will be given by a separate, new IV line. You will be given 250 mL (about 1 cup, 8 oz.) each day for 5 days or until you leave the hospital, if that comes first.

If you are placed in the PF-814 study group, you may need to have some extra blood taken before the infusion and on Day 5. This extra blood will be less than 3 mL (about half a teaspoon). Your doctor may also recommend taking extra blood on other days.

If you are placed in the PF-814 study group, we strongly advise you to not have sex that could make you or a partner pregnant for 5 weeks after you get the infusion. The study team will discuss acceptable forms of contraception with you.

3A

ACTIV 3 v5.1
17NOV2021

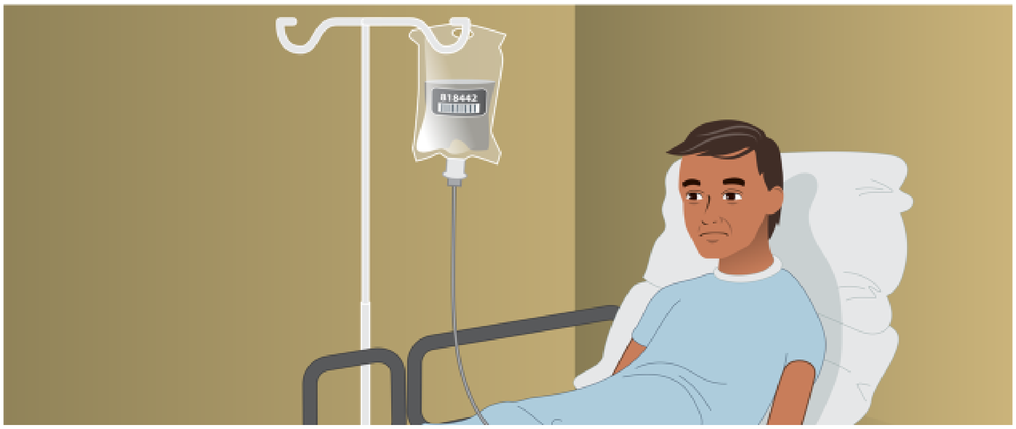

What could be the side effects from PF-814?

PF-814 was studied in healthy people who did not have COVID-19. It was well tolerated, and no one developed serious side effects. PF-814 was also studied in people in the hospital with COVID-19. Most side effects were mild or moderate. A few serious side effects that are known complications of COVID-19 were seen in both the PF-814 and placebo arms. They were not thought to be due to PF-814.

We will watch you carefully and treat you right away if you have a reaction or side effects that need treatment. In some cases, we may stop the PF-814 infusion for a time or stop it completely.

4A

ACTIV 3 v5.1
17NOV2021

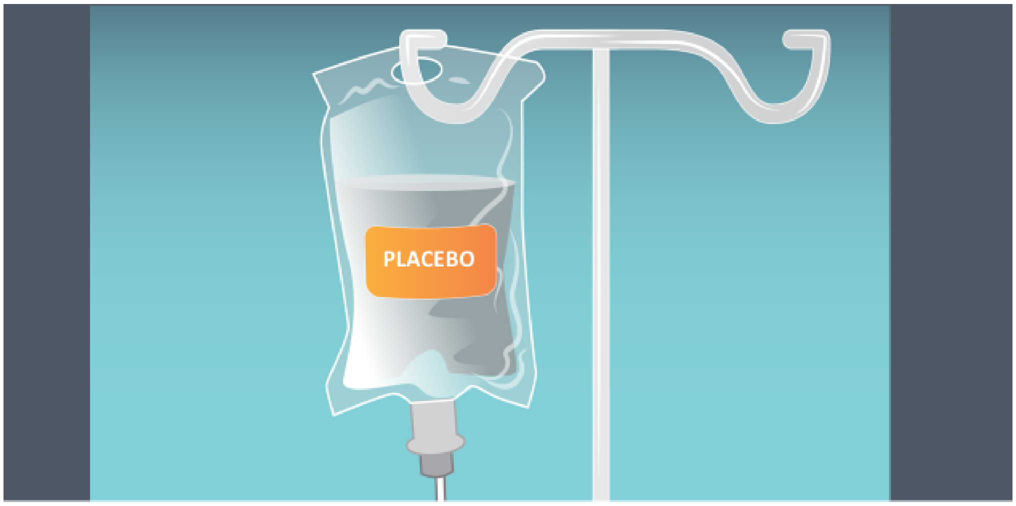

What is a placebo?

Placebos are being used in this study. A placebo is a liquid that looks like the study drug but does not have any drug in it. It should not make you feel better or worse. Placebos are given in the same amount and for the same time as the study drug.

The placebo in this study is salt water and is given as a drip or infusion through a plastic tube into a vein in your arm.

1B

ACTIV 3 v5.1
17NOV2021

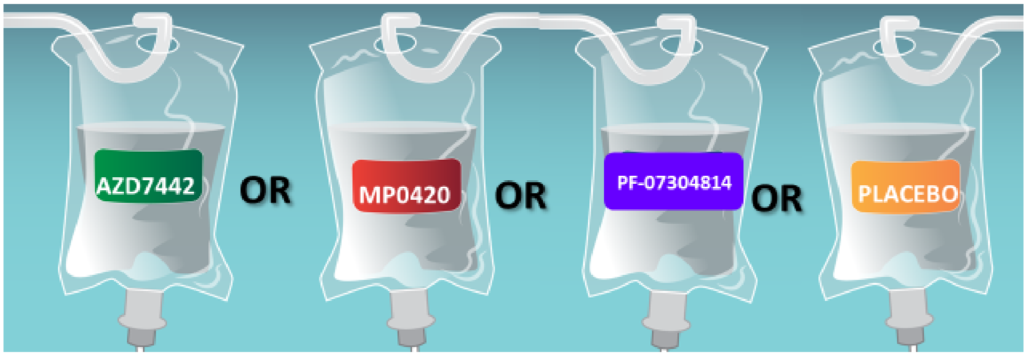

What is my chance of getting a study drug?

Whether you get one of the study drugs or placebo is decided by chance – like flipping a coin. Your chance of getting a study drug depends on how many of the 3 study drugs you would be able to receive. You will always have a 50% (50:50) chance or greater of getting study drug. You and the study staff will not know if you are getting a study drug or placebo.

If you can receive any of the three study drugs, you will have a 60% (60:40 or 3 in 5) chance of receiving one of the study drugs. If you can receive PF-07304814 and either AZD7442 or MP0420, you will have a 50% (50:50 or 1 in 2) chance of receiving a study drug. If you can receive either AZD7442 or MP0420 (but not PF-07304814), you will have a 67% (2:1 or 2 in 3) chance of getting a study drug.

Study Drugs Able to Get	Chance of Getting a Study Drug and not Placebo
AZ or MP	67% (2:1 or 2 in 3)
Any of the 3 (AZ or MP or PF)	60% (60:40 or 3 in 5)
AZ or PF, MP or PF, AZ only, MP only, PF only	50% (50:50 or 1 in 2)

2B

ACTIV 3 v3.3
27APR2021

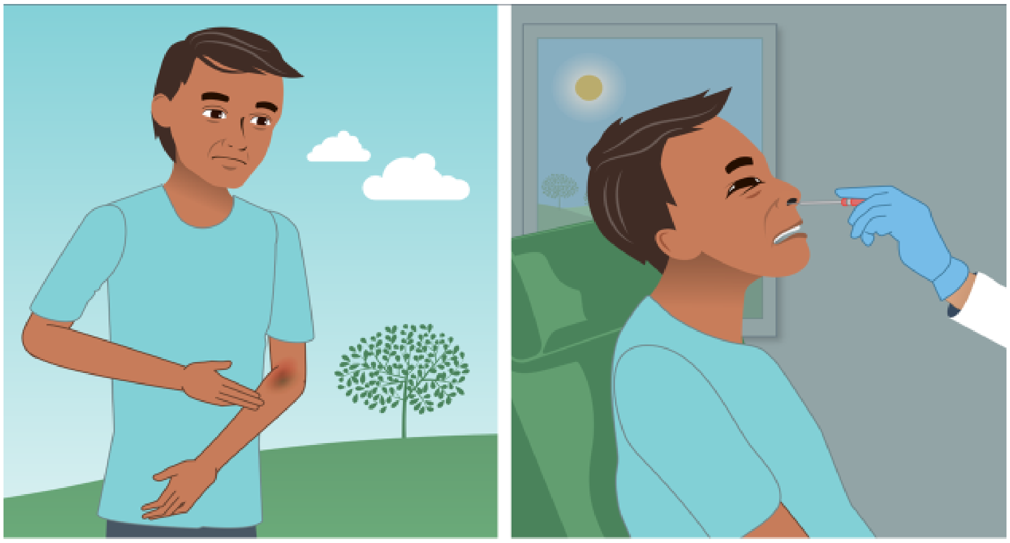

Are there other risks or discomforts related to this study?

The needle used to draw blood or place an IV line can hurt. You may get a bruise where the needle went in. Sometimes drawing blood causes people to feel lightheaded or even to faint. There is a very small risk of getting an infection where the needle went into the vein. This could be treated with antibiotics.

The nose swab may cause discomfort, sneezing, bleeding, or make your eyes water.

3B

ACTIV 3 v5.1
17NOV2021

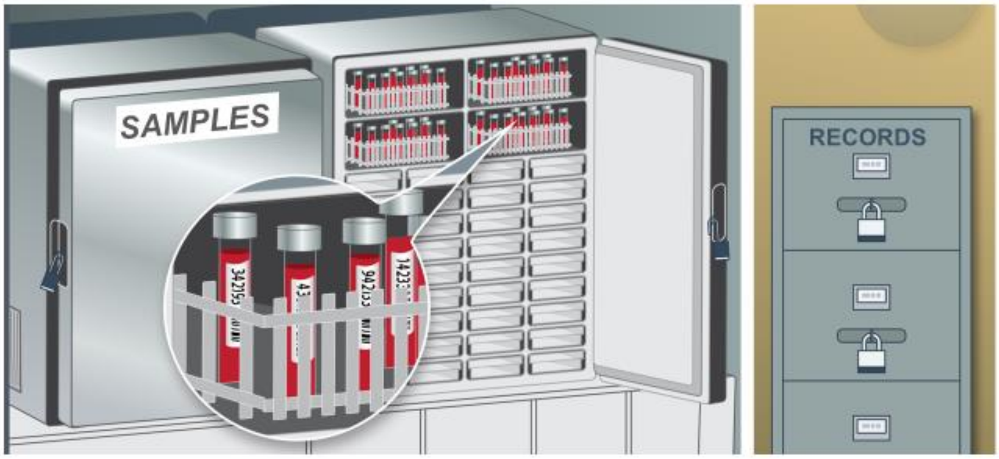

What will happen to your samples and personal information?

Your samples and study information will be coded. Your name and personal details are never used. Your coded information will be sent to the University of Minnesota in the United States (US). Your coded blood and swab samples will be tested and stored in a US central laboratory. Any unused samples and your coded data will be stored for future COVID-19 research tests.

We will not sell your samples. Your coded study information and samples may be shared with other researchers and the pharmaceutical companies that made the study drug, to help learn more about its effects. You and your doctor will not get results from these research tests. If you change your mind and decide you do not want us to store your study samples or information, please let us know.

1C

ACTIV 3 v5
22JUN2021

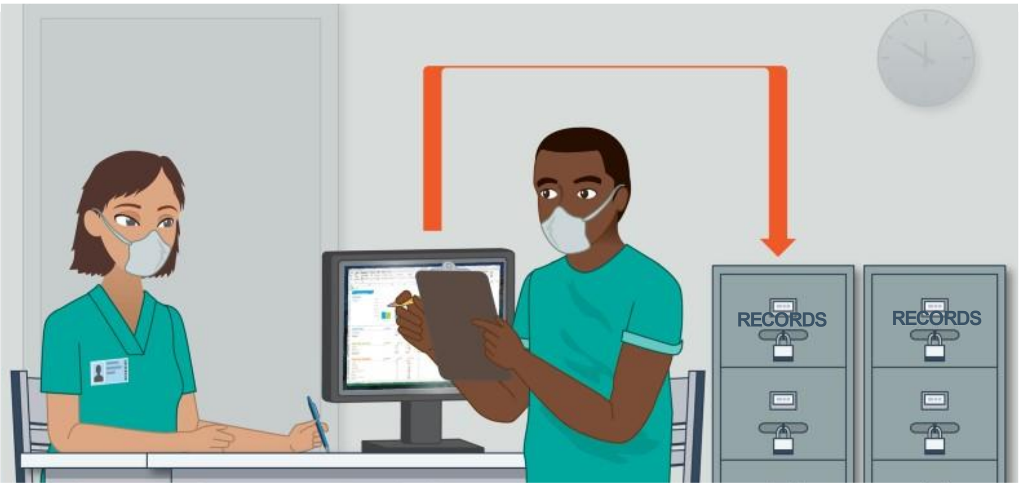

How will your privacy be protected?

Your personal information will be treated with great care. It will only be seen by people who are linked to the study and are required to protect your privacy, such as:

- The ethics committee that protects your rights as a participant in the study
- The US National Institutes of Health, the sponsor paying for the study
- Research staff and staff who monitor the study
- Health agencies in countries where the study is being done, such as the US FDA (Food and Drug Administration).

Your health information and samples will be coded to remove your name and personal details before they leave this study site. Your rights over your samples and data are described in the consent document.

2C
ACTIV 3 v5
22JUN2021

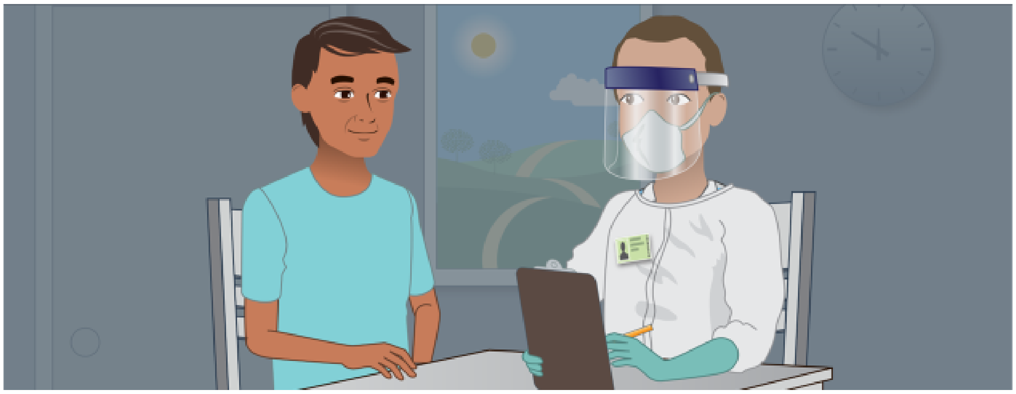

What else should you know about study participation?

- We will ask you for contact information for two people who are close to you in case we cannot reach you after you leave the hospital.
- We will give you the study drug (or placebo) and remdesivir at no cost. Information about hospital and other costs related to your illness is given in the informed consent document.
- Details about what will happen if you are hurt because of this study are given in the informed consent document.
- A description of this clinical trial will be available at ClinicalTrials.gov, and on the EudraCT website (eudract.ema.europa.eu).

3C
ACTIV 3 v5.1
17NOV2021

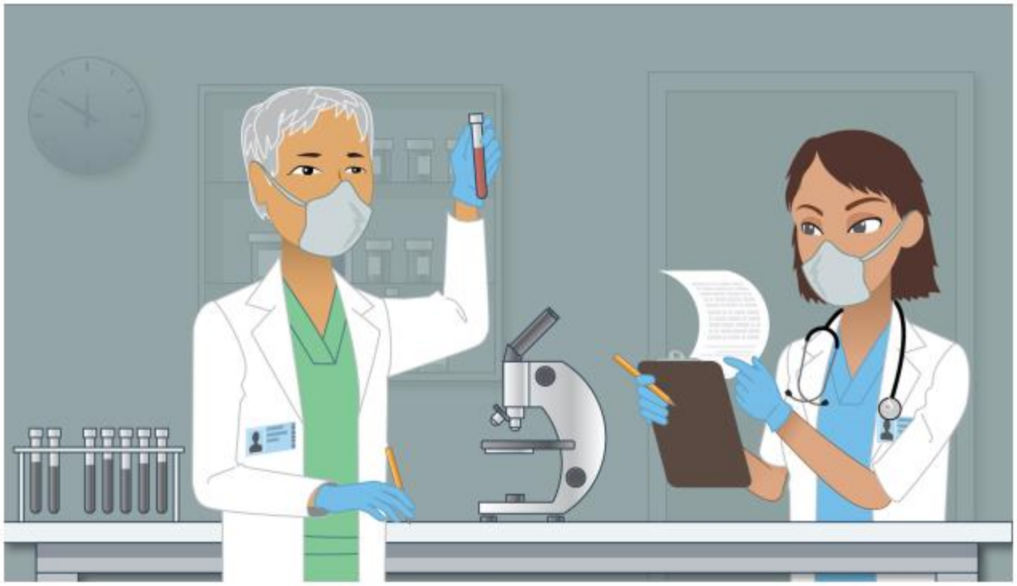

Additional consent for genetic testing on stored specimens

The study team would like your permission to collect a small amount of your blood and store it for researchers who will do genetic testing (testing on your genes) and other related tests in the future. These tests will help them understand how the genetic make-up of people affects the response to the COVID-19 virus.

1D

TESICO v1.0
27APR2021

Supplementary Figure 11. Participant Brochure. This was created as a Trifold, double-sided, single sheet with participant instructions to monitor temperature and oxygen saturation levels at home as required by the study. It contained study name, step-by-step approach for measuring temperature and peripheral oxygen saturation levels, instructions for what to do if temperature or oxygen saturation is out of range, customizable contact information, and a table for recording temperature and peripheral oxygen saturation measurements.

Temperature and Blood Oxygen Measurements

COVID-19 can make oxygen levels in your blood drop and may cause a fever. As part of this research study, you will be asked to measure and record your blood oxygen levels and temperature by mouth every day for 7 days.

This brochure describes how to measure your blood oxygen level and temperature by mouth at home, and when to seek medical advice. Study staff will contact you by phone to obtain these measurements.

What devices will you receive?

- Thermometer
- Pulse Oximeter – a small device that you place on your finger to measure blood oxygen levels (SpO_2% reading)

How often should you check your temperature and oxygen?

At least once per day, preferably at the same time each day. Check more often if you're feeling feverish or short of breath or have any other symptoms that concern you.

Instructions for Temperature Measurement

1. Wait 15 minutes after eating or drinking to take temperature.
2. Turn thermometer on and wait for a tone. The window will display 188.8F followed by a test temperature.
3. Place the thermometer under your tongue in the back corner of your mouth and close your lips.
4. When the thermometer beeps, take it out of your mouth and record temperature in **Daily Recordings** chart.

Instructions for Blood Oxygen Measurements

Follow the basic steps below. Check the specific device instructions for details. Remain calm and seated for 5 minutes prior to performing measurement.

1. **Prepare** – Prepare your fingertip. Make sure fingertip is clean, dry, warm, and free of fingernail polish and artificial nails.
2. **Warm** – Warm your hands if necessary and keep your fingers relaxed.
3. **Turn on** – Turn on the pulse oximeter per specific device instructions (remember to check the batteries).
4. **Place** – Place your finger into the pulse oximeter with your fingernail up and keep finger still.
5. **Wait** – Wait for the screen to show a number. **If the "SpO_2%" reading is less than 94, wait 3 minutes and repeat all steps carefully.**
6. **Repeat, if necessary** – If a number doesn't appear, carefully repeat steps 1 to 5 using a different finger.
7. **Record** – Use the table on the other side of this brochure to record your SpO_2% readings.

Checking Your Temperature and Oxygen Levels at Home

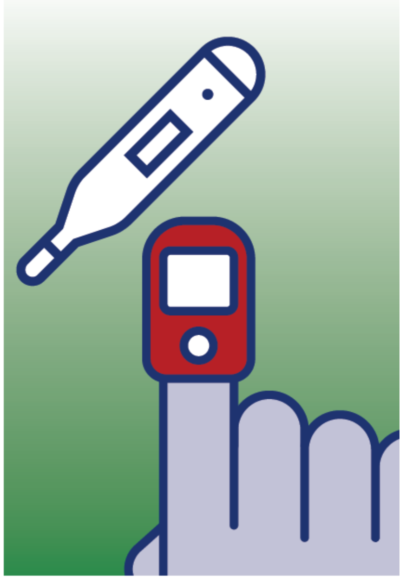

insight

OTAC INSIGHT012

Participant Name:

Oxygen (SpO_2%) Measurements

Oxygen reading is greater than or equal to 94

Record the reading.

Oxygen reading is less than 94

Wait 3 minutes and take another one. Record the readings. **If still less than 94, follow contact instructions.**

If before your COVID-19 illness, your oxygen measurements were usually less than 94, and/or you are on home oxygen, and oxygen reading is less than 90

Wait 3 minutes and take another one. Record the readings. **If still less than 90 or you have to increase your level of home oxygen, follow contact instructions.**

Contact Instructions

If SpO_2% readings are less than 94 (or less than 90 per instructions above) or you are having shortness of breath, contact:

During office hours: **Outside office hours:**

Seek medical help immediately if you experience any of the following COVID-19 symptoms:

- Feeling breathless or unable to catch your breath
- Persistent pain or pressure in the chest
- Difficulty waking up or staying awake
- Severe muscle aches or tiredness
- Shakes or shivers
- Illness is worsening quickly and/or you are feeling confused
- Unable to complete simple daily tasks like dressing yourself or making food
- Any other symptoms that are severe or concerning to you

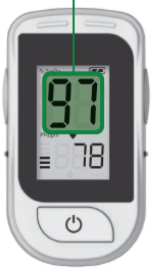

As shown above, the oxygen level number to record will appear under the "%SpO_2" setting on the oximeter.

The accuracy of pulse oximeter readings varies and may not be as accurate for some people with darker skin. Although the effect of skin color is probably not significant when pulse oximetry readings are above 94%, please follow the instructions for medical care if you have any concerns about your health.

Temperature and Oxygen Measurements

Use the table below to record temperature by mouth and oxygen (SpO_2%) daily at about the same time each day.

DAILY RECORDINGS		
Date and Time	Temperature	Oxygen Level
AM PM	°C/F	SpO_2%
AM PM	°C/F	SpO_2%
AM PM	°C/F	SpO_2%
AM PM	°C/F	SpO_2%
AM PM	°C/F	SpO_2%
AM PM	°C/F	SpO_2%
AM PM	°C/F	SpO_2%
AM PM	°C/F	SpO_2%
AM PM	°C/F	SpO_2%
AM PM	°C/F	SpO_2%

Supplementary Figure 12. Thank You Letters. The letters were designed to express gratitude and highlight the importance of participating in research, the benefits it brings to the community and society, as well as the contribution to the body of knowledge in a difficult time of pandemic. They also contained a summary of study results and instructions to contact the study site for additional information.

insight

International Network for Strategic Initiatives in Global HIV Trials
Leadership Group
University of Minnesota
2221 University Avenue Southeast, Suite 200
Minneapolis MN USA 55414-3080

ADD DATE

Dear Study Participant,

Thank you very much for being in the **TICO** (**T**herapeutics for **I**npatients with **CO**VID-19) and **VATICO** (**Va**ccina**t**ion Strategies for Recovered **I**npatients with **CO**VID-19) research studies. We would like to give you an update on these studies and an overview of the available study results.

We conduct clinical research studies, such as TICO, to find safe treatments that help people with COVID-19 recover faster and leave the hospital sooner. Studies can discover which treatments work best and which don't work. VATICO was done to find the best timing and number of COVID-19 vaccine doses to prevent future serious illness in people who had recovered from severe COVID-19.

In TICO, we enrolled 2,753 participants at 115 hospitals in Denmark, Greece, Nigeria, Poland, Singapore, Spain, Switzerland, Uganda, the United Kingdom, and the United States of America. Some TICO participants are still in follow up.

TICO compared six investigational drugs to placebos. Placebos look like the investigational drug but do not have any drug in them. Placebos should have no effect on the participant. We wanted to see whether the drugs are safe and help study participants. **Each TICO participant received only one of the investigational drugs in the table below, or placebo, in addition to standard care for COVID-19.** Here are the TICO study results so far. Results for the investigational drug you helped to study are highlighted. *[Site staff: please highlight the row with the arm in in which the participant was randomized.]*

Investigational Drug	Type of Drug	Showed Benefit?	Safe for Participants?
**LY-CoV555** (also known as LY3819253 or bamlanivimab, developed by Eli Lilly)	Monoclonal antibody	No.	Yes.
		Participants who had low levels of antibodies against the virus that causes COVID-19 seemed to do better than those with high levels of antibodies. Antibodies are substances produced by the immune system to fight the virus.	
**VIR-7831** (also known as sotrovimab or Xevudy, developed by GlaxoSmithKline and Vir Biotechnology)	Monoclonal antibody	No	Yes
**BRII-196 plus BRII-198** (also known as amubarvimab/romlusevimab, developed by Brii Biosciences)	Two monoclonal antibodies	No	Yes
**AZD7442** (also known as tixagevimab/cilgavimab or Evusheld, developed by AstraZeneca)	Two monoclonal antibodies	Yes. Did not improve the time to recover from COVID-19, but led to a 30% decrease in deaths. Up to 90 days after receiving AZD7442 or placebo, 9% of participants in the AZD7442 group died compared with 12% in the placebo group.	Yes

<table>
<tr><th>Investigational Drug</th><th>Type of Drug</th><th>Showed Benefit?</th><th>Safe for Participants?</th></tr>
<tr><td>MP0420
(also known as ensovibep, developed by Molecular Partners)</td><td>DARPin protein</td><td>No</td><td>Yes</td></tr>
<tr><td>PF-07304814
(developed by Pfizer)</td><td>Protease inhibitor anti-viral</td><td colspan="2">The benefit and safety could not be assessed because the study of this drug was not completed. The US Food and Drug Administration (FDA) paused the study of PF-07304814 when 58 participants had been enrolled. Pfizer decided to stop all further investigation of PF-07304814.</td></tr>
</table>

VATICO was designed to compare the following four COVID-19 vaccination strategies in TICO participants:

- One vaccine dose immediately after enrollment
- One vaccine dose 12 weeks after enrollment
- First vaccine dose immediately after enrollment plus a second dose 4 weeks later
- First vaccine dose 12 weeks after enrollment plus a second dose 4 weeks later

Enrollment into VATICO was stopped in February 2022 after 66 participants had been enrolled because the enrollment goal of 640 total participants was not going to be achieved. Some participants are still in follow up.

The dedication and willingness of participants like you have helped the TICO and VATICO studies to provide high-quality data. We are truly grateful for your commitment and effort in long-term studies like these where follow up was not always convenient or easy.

Your efforts have helped evaluate possible treatments for people hospitalized for COVID-19, which are much needed. The TICO study team has published articles in major medical journals that provide more details about what we have learned in TICO. The VATICO study team has published an article about the challenges faced and lessons learned in the study. If you would like copies of the papers, or have any questions about these studies, please contact your research study site.

What we have learned in TICO will help us design future studies to find treatments to help people in the hospital with COVID-19. We hope our experience with VATICO will help researchers do other studies that answer important questions about vaccination after recovery from COVID-19.

People like you who devote time and effort to clinical research studies make medical advancement possible. Thank you again for participating in these studies.

Sincerely,

Prof. James Neaton, PhD, Principal Investigator, INSIGHT

Prof. Jens Lundgren, MD, Chair, TICO and VATICO Studies

Supplementary Figure 13. Letter for Participants to Share with mRNA COVID-19 Vaccine Providers. This letter contained one page for the participant to keep and the other pages are for the participant to give to the provider. This was particularly important in a setting of vaccination requirement by many institutions and workplaces.

Letter for VATICO Participants to Share with mRNA Vaccine Providers

Dear VATICO Participant,

Thank you for agreeing to participate in the "Vaccination for Recovered Inpatients with COVID-19 (VATICO)" study. The study team recommends that you take this letter with you when you get your mRNA vaccine to ensure you get your vaccines as planned for the study. You can go to any type of vaccination center (e.g., a pharmacy, public health facility, or temporary vaccine administration center). This letter will help your vaccination center understand the study. The vaccination center staff may have questions about the study, as your study vaccination schedule differs from when the vaccination center would give most people (i.e., people who have not yet had COVID-19) their COVID-19 vaccines. They may ask you questions about the timing of the vaccine and number of doses. The attachment provides answers to their anticipated questions.

You were randomized to receive a single dose, or 2 doses, of the vaccine. Your window for receiving your first vaccine is _____/_____/_____ to _____/_____/_____. If applicable, your second dose will follow approximately 4 weeks later. Contact information for your site is provided at the end of the letter in case you or the vaccination center have further questions on the study.

As you know, these vaccines have been given to millions of people worldwide. Vaccination administration is part of your routine clinical care, and it is recommended, regardless of whether you had chosen to take part in this study or not, that you get vaccinated against COVID-19. If you have further questions on the study and your participation, please contact your study site.

Thank you,

The VATICO Study Team

Study Coordinator

Name: ________________________ Phone: _______________________

Site Investigator

Name: _______________________ Phone: _______________________

Letter for VATICO Participants to Share with mRNA Vaccine Providers

Dear Vaccine Provider,

The person presenting this letter is taking part in a study ("Vaccination for Recovered Inpatients with COVID-19" [VATICO]) to determine the optimal timing and number of doses of COVID-19 mRNA vaccines in people who have recovered from severe COVID-19 infection. This person has already taken part in an inpatient study ("Therapeutics for Inpatients with COVID-19" [TICO]) assessing treatments for people hospitalized with COVID-19, and has agreed to take part in this follow-up study. The questions and answers below are intended to inform you about the study and the participant's role.

Overview of the VATICO study objectives

The optimal timing and number of mRNA vaccine doses against severe acute respiratory syndrome coronavirus 2 (SARS CoV-2) in people who have recovered from severe COVID-19 infections has not been determined. To help answer this question, this open label study with mRNA vaccines is examining two different dosing schedules (immediate versus deferred) and number of doses (one versus two doses). Some of these participants may have received monoclonal antibodies or other investigational products during their hospital stay due to their participation in the TICO study.

All persons in the study will receive mRNA vaccination. The participant has received an additional paper with the schedule for their dose(s). It is recognized that this vaccination dosing regimen differs from standard dosing guidelines for the general population, including people who may have received monoclonal antibodies. However, it is important the participant receives their vaccination dose(s) in accordance with the protocol schedule, in order to meet the study objectives. Thank you for your assistance.

1) **Why is the VATICO study being performed?**
   Answer: This trial will try to provide evidence to optimize the vaccination dosing schedule for people who have recovered from severe COVID-19 infection. Specifically, it is trying to find out the best time to give the vaccine (immediate versus deferred) and the optimal number of doses of mRNA vaccine (one versus two doses) to administer to these individuals.

2) **Who is sponsoring the VATICO study?**
   Answer: The study is being Sponsored by the National Institutes of Health (NIH) and supported by their Division of Clinical Research. It is being conducted at clinical trials sites globally through networks coordinated by the University of Minnesota INSIGHT network. It has been approved by an Institutional Review Board.

3) **What vaccines are being studied?**

Answer: Two mRNA COVID-19 vaccines are being used in the VATICO study: Moderna mRNA-1273 and Pfizer BNT 162b2. Both vaccines have received emergency use authorization by the FDA in the US and by many other regulatory authorities in other countries.

4) **Why are you studying a different dosing schedule?**
Answer: The optimal timing and number of vaccinations against severe acute respiratory syndrome coronavirus 2 (SARS-CoV-2) for patients hospitalized due to COVID-19, after having recovered, has not yet been determined.

5) **Why early versus deferred dosing for the first vaccination?**
Answer: The study is comparing immediate vaccination after recovery to delayed vaccination after recovery. After the acute infection, more time might allow for a stronger immune response, but this has not yet been established.

6) **What is the rationale for studying one dose vs. two?**
Answer: People who have already had COVID-19 have already had their immune systems introduced to the SARS-CoV-2 virus which causes COVID-19 disease. In effect, their immune systems may be primed by their prior SARS-CoV-2 infection. Such people may only need one dose. However, two doses of mRNA vaccines are currently recommended in mRNA SARS-CoV-2 vaccination guidelines. This study will compare the immune responses of one versus two mRNA vaccines.

7) **What are the possible dosing regimens?**
Answer: There are 4 different dosing regimens that participants can be randomized to:
a) Immediate dosing with one dose only
b) Immediate dosing of first dose; second dose approx. 4 weeks later
c) Deferral of 12 weeks with one dose only
d) Deferral of 12 weeks with first dose; second dose approx. 4 weeks later

The participant has been randomized to option ____ (see above options for further information) and is eligible for vaccination with an mRNA vaccine between _____/_____/_____ to _____/_____/_____ (date range for dose 1) and _____/_____/_____ to _____/_____/_____ (date range for dose 2, if applicable).

8) **Who should I contact if I have more questions?**
Answer: For further information on the study, please call the participant's site investigator:

Name: ______________________ Phone: ______________________

Supplementary Figure 14. Proof of Recovery Letter and mRNA COVID-19 Vaccination Card.

These were designed to be a record of COVID-19 status and participation in a vaccine study to mitigate constraints on unvaccinated persons. It was a customizable document provided to sites to complete, print on local letterhead, and give to participants.

INSTRUCTIONS FOR SITES: Each VATICO participant needs to receive:
This letter (proof of recovery) below on Official letterhead:
Completed vaccination card (proof of vaccination & study participation): pg 2

INSERT OFFICIAL INSTITUTE LETTERHEAD with the name, address, and phone number of a licensed healthcare provider or public health official

---

To whom it may concern, this letter provides the following information:

For VATICO Participant
Patient Name: ______________________
Patient Address: ______________________
Patient Date of Birth: ______________________

Patient's health care provider:
Name: ______________________
Address: ______________________
Phone number: ______________________

Proof of Positive COVID-19 Test
- Date: ______________________
- Attached: patient needs paper or electronic copy of positive test results, dated no more than 90 days previously

The individual has recovered from COVID-19, is cleared to end isolation and therefore can travel, return to work or school, whichever is applicable.

VACCINATION RECORD CARD TO FILL OUT AND GIVE TO VATICO PARTICIPANT, 2 sided

VATICO COVID-19 Trial

Participant ID number: [Insert ID no.]
Participant Name:
Participant DOB:
DD-MMM-YYYY

The holder is a participant in the **VATICO vaccine trial**. They have received Moderna or Pfizer vaccine on the date and time as detailed overleaf.
In case of emergency please contact:
[Insert local contact details including out-of-hours]

VATICO COVID-19 vaccination card

Participant Name:
Participant DOB:
Vaccine 1: Name of vaccine: Moderna mRNA-1273 or Pfizer BNT 162b2
Batch Nos: Expiry date:
DATE VACCINE GIVEN:
DD-MMM-YYYY
Name of vaccine administrator:
Location of vaccine administator

Vaccine 2 (if applicable) Name of vaccine: Moderna mRNA-1273 or Pfizer BNT 162b2
Batch Nos: Expiry date:
DATE VACCINE GIVEN:
DD-MMM-YYYY
Name of vaccine administrator:
Location of vaccine administrator:

Supplementary Figure 15. Protocol Quick Reference Guide. This was developed in a two-page format and contains key points about the study for clinical and site staff. Some of the items included were study name, diagram of study design, timeline of protocol assessment activities, primary objective and endpoint, clinical assessment scale, detailed inclusion and exclusion criteria, and customizable local site contact information.

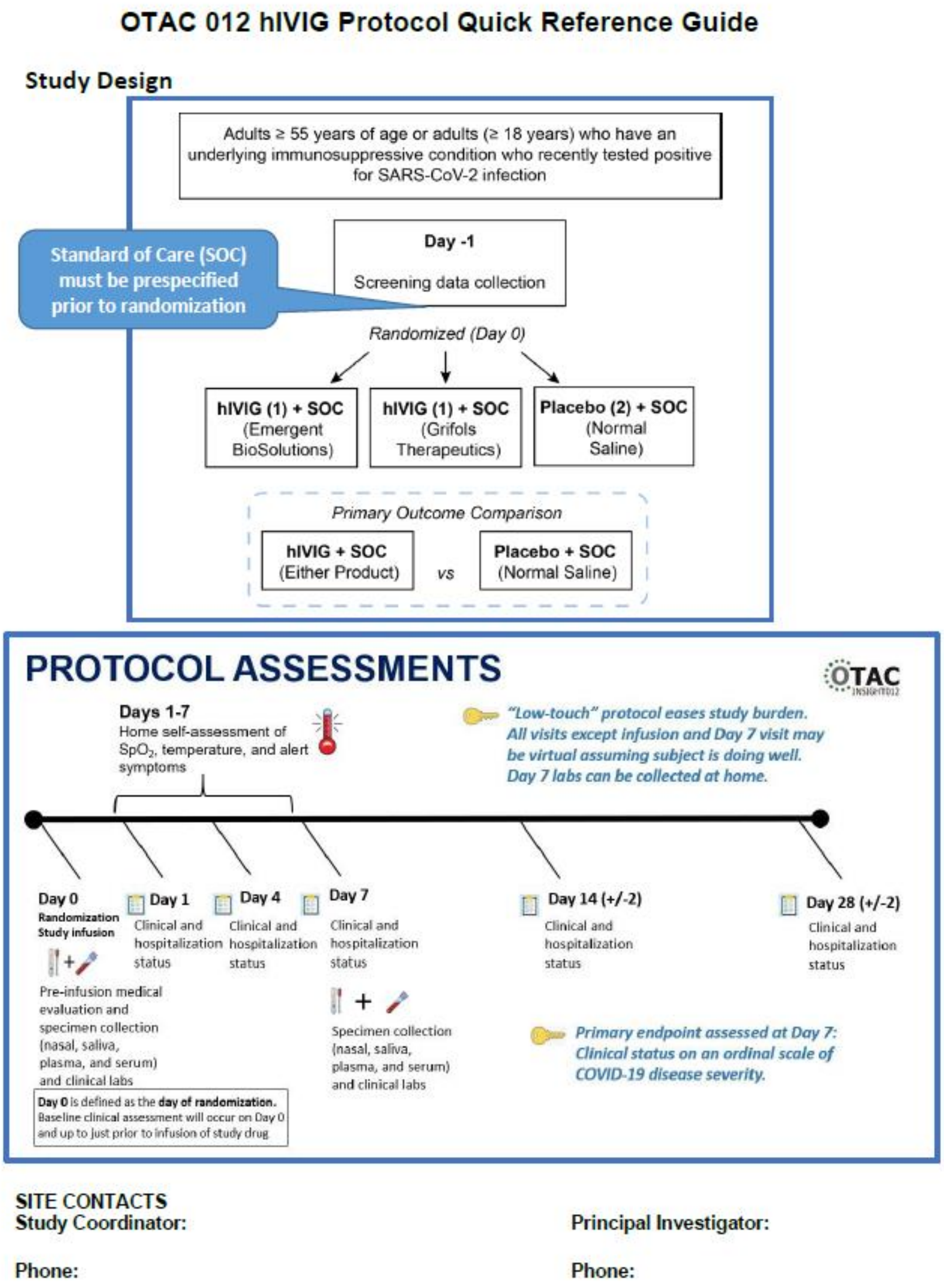

OTAC 012 hIVIG Protocol Quick Reference Guide

Primary objective: Compare the safety and efficacy of a single infusion of anti-COVID-19 hIVIG versus placebo among adults with recently diagnosed SARS-CoV-2 infection who do not require hospitalization.
Primary endpoint: Clinical status at Day 7, defined by an ordinal scale.

Ordinal Scale for Clinical Status		
	Category	Clinical Severity
Better ↑	1	Asymptomatic and no limitations in usual activity due to COVID-19
	2	Mild COVID-19 symptomatic illness or minor limitations to usual activity
	3	Moderate COVID-19 illness and major limitations to usual activity
		↑ Eligible for Enrollment ↑
	4	Severe COVID-19 or serious disease manifestation from COVID-19
Worse ↓	5	Critical illness from COVID-19 or Death

Inclusion Criteria

- ✓ ≥ 55 years of age **OR** ≥ 18 and immunocompromised/suppressed
- ✓ Positive test for SARS-CoV-2 within ≤ 5 days. Tests may include nucleic acid amplification test (NAAT) or any protocol-approved rapid test
- ✓ If symptomatic, symptom onset within ≤ 5 days
- ✓ Agrees not to participate in another treatment trial through Day 7, unless hospitalized or disease progresses significantly (defined by ordinal category 4 or 5)
- ✓ Participant provides written informed consent prior to study procedures and understands and agrees to adhere to study procedures

Exclusion Criteria

- ⃠ Asymptomatic and had symptoms of acute infection that have resolved (for >24 hours)
- ⃠ Asymptomatic and has received a vaccination for COVID-19 (≥1 dose)
- ⃠ Undergoing evaluation for hospital admission for medical management (not for public health purposes)
- ⃠ Evidence of pneumonia and/or hypoxia due to COVID-19
- ⃠ Prior receipt of immunoglobulin product or passive immune therapy for SARS-CoV-2 in the past 90 days
- ⃠ Known thrombotic diathesis, recent thrombosis, or procoagulant conditions or disorders
- ⃠ History of hypersensitivity to blood, plasma or IVIG excipients
- ⃠ Known IgA deficiency or anti-IgA antibodies
- ⃠ Medical condition for which receipt of 300 mL volume of IV fluid for study treatment may pose risk (for example, decompensated congestive heart failure)
- ⃠ In the opinion of the investigator, any condition for which participation would not be in the best interest of the participant or that could prevent or confound protocol assessment and adherence to planned study procedures through Day 28

SITE CONTACTS
Study Coordinator:

Phone:

Principal Investigator:

Phone:

Supplementary Figure 16. Study Summary Presentation. The PowerPoint presentation contained information about the scientific rationale for the study, study hypothesis, study product, potential advantages of study product over convalescent plasma and monoclonal antibodies, primary objective and endpoint, study design, inclusion and exclusion criteria, aim of the study, schedule of assessments, and duration of study participation. It was developed to provide a summary of the study for healthcare providers and other non-study medical personnel.

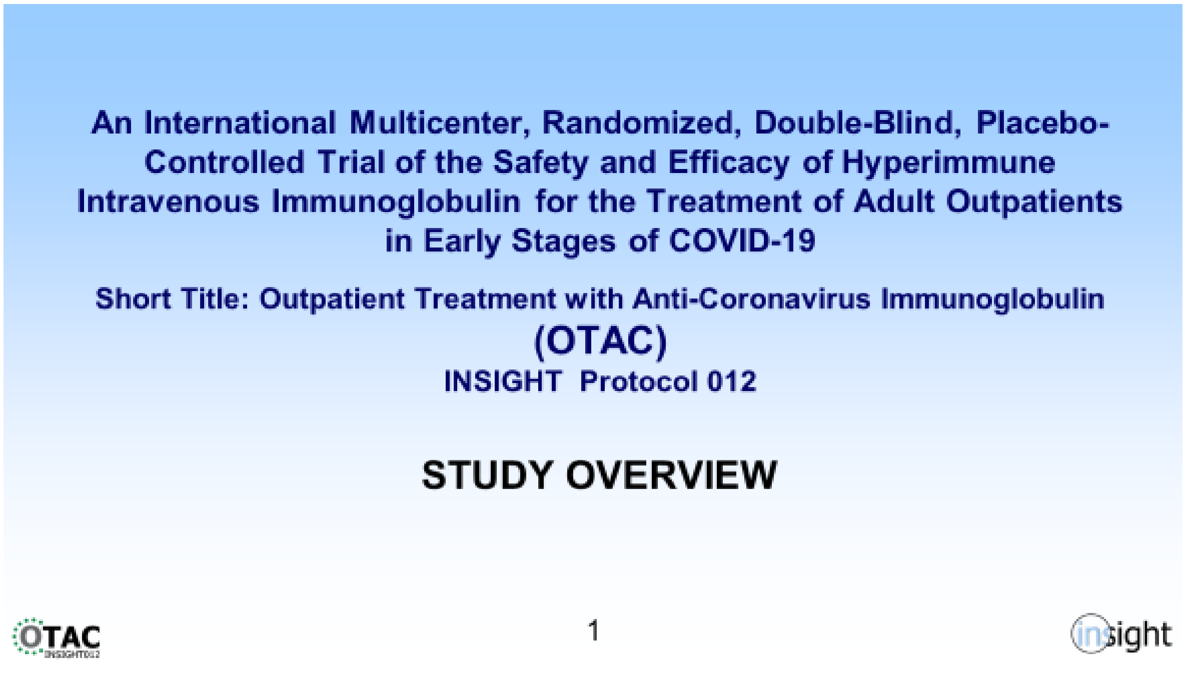

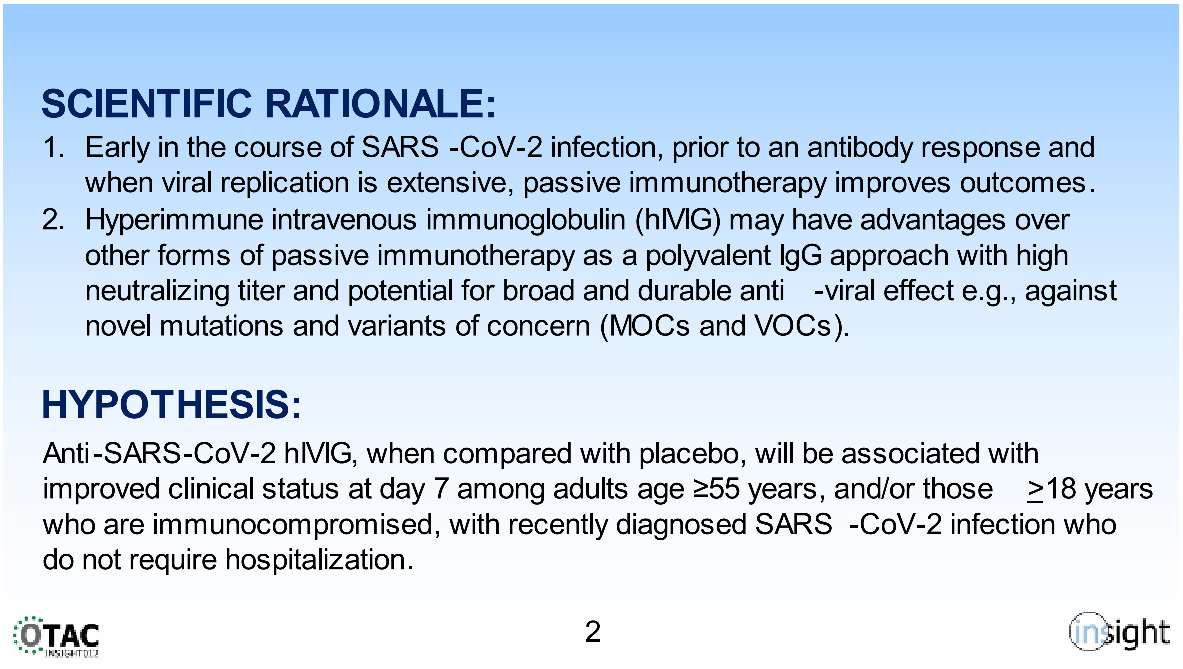

OTAC INSIGHT012 — What are advantages of hIVIG over Convalescent Plasma (CP)?

hIVIG is consistently higher potency

- Concentrated IgG without other plasma components
- Multiple recovered donors, broad polyvalent product
- Standardized with more consistent potency than CP
- Higher neutralizing potency than 'high-titer' CP

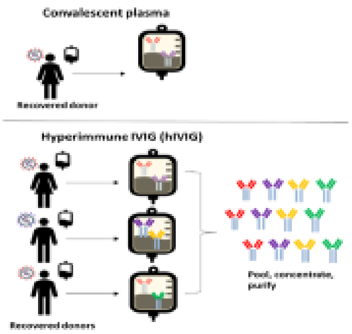

Potency of COVID-HIG and Low- and High-Titer CP

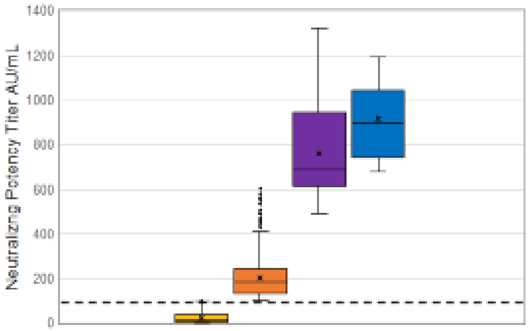

CP (Low titer <100 AU/mL) CP (High Titer ≥ 100 AU/mL)
[redacted] COVID-HIG [redacted] COVID-HIG

NOTE: FDA guidance for high-titer CP (i.e., by Ortho Vitros IgG ≥9.5) reflects dashed line at titer ~ ≥100AU/mL (AU = Alliance IgG standard units); Data presented for Grifols and Emergent HIG include 2020 lots

insight

OTAC INSIGHT012 — What are advantages of hIVIG over Monoclonal Antibodies (MAbs)?

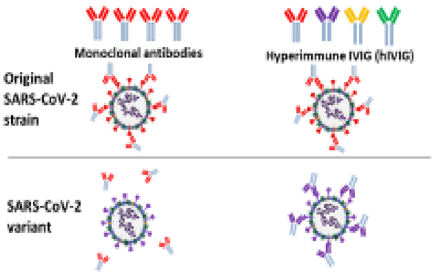

hIVIG has broader activity

- Pooled from multiple donors that have recovered from COVID
- Targeting more epitopes than either CP or individuaMAbs
- hIVIG maintains activity against VOCs (e.g., E484K)

Pseudovirus (Variant) Testing* by US -FDA:

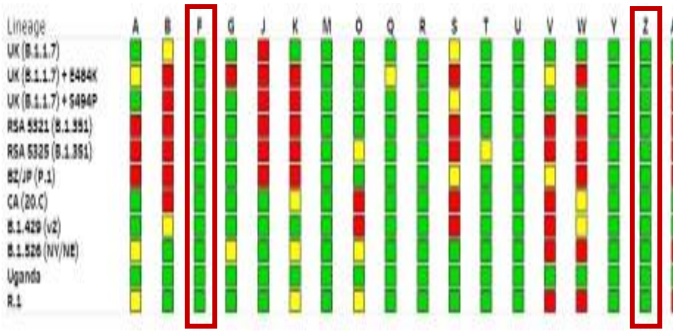

**Fold reduction criteria (ratio of WT IC_{50}/Variant IC_{50}) is green for <5-fold, yellow for >5<50 fold, and red for >50 fold.*

Key: ***Column F = Grifols hIVIG***
Column Z = Emergent hIVIG
Other columns = SARS-CoV2 monoclonal antibodies

insight

OTAC INSIGHT012

PRIMARY OBJECTIVE AND ENDPOINT

Primary objective: Compare the safety and efficacy of a single infusion of anti-COVID-19 hIVIG versus placebo among adults with recently diagnosed SARS-CoV-2 infection who do not require hospitalization.

Primary endpoint: Clinical status at Day 7, defined by an ordinal scale.

Better ↑ / Worse ↓

Ordinal Scale for Clinical Status	
**Category**	**Clinical Severity**
1	Asymptomatic <u>and</u> no limitations in usual activity due to COVID-19
2	Mild COVID-19 symptomatic illness or minor limitations to usual activity
3	Moderate COVID-19 illness <u>and</u> major limitations to usual activity
	↑ Eligible for Enrollment ↑
4	Severe COVID-19 or serious disease manifestation from COVID-19
5	Critical illness from COVID-19 or Death

6 insight

OTAC INSIGHT012

STUDY DESIGN

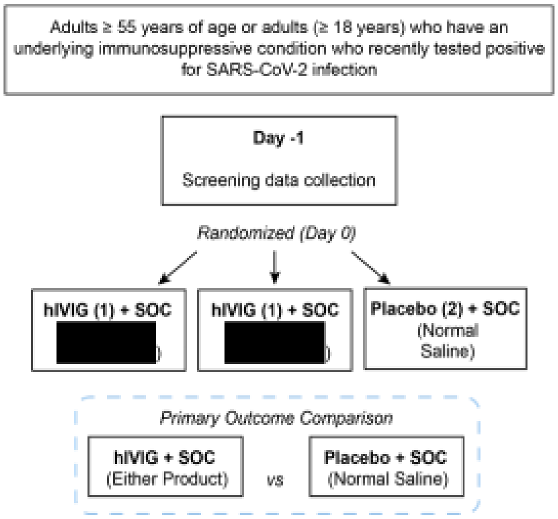

- **Standard of Care (SOC):** available and endorsed by guidelines; **<u>pre-specified before randomization</u>**
  - **Stratum 1**: SOC does not include anti-SARS-CoV-2 monoclonal antibody (MAb)
  - **Stratum 2**: SOC with intent to give anti-viral MAb; expectation is up to 20% of overall study

Primary Endpoint: Clinical Status on ordinal scale at Day 7	
**Key Efficacy Subgroup Stratum 1 (n=660)**	**Entire Study Population Stratum 1 + 2 (n=820)**
hIVIG + SOC **(no MAb)**	hIVIG + SOC **(all types)**
versus	versus
Placebo + SOC **(no MAb)**	Placebo + SOC **(all types)**

7 insight

Eligibility Criteria

***OTAC is evaluating the value of standard of care (SOC) + hIVIG versus SOC alone.* Flexible concomitant SOC is allowed in keeping with local guidelines.**

Inclusion

- ≥55 years of age **OR**
  ≥18 and immunocompromised/suppressed
- Positive test for SARS-CoV-2 within ≤ 5 days. Tests may include nucleic acid amplification test (NAAT) or any protocol-approved rapid test
- If symptomatic, symptom onset within ≤ 5 days
- Agrees not to participate in another treatment trial through Day 7, until hospitalized or unless disease progresses significantly (defined by ordinal category 4 or 5)
- Participant provides written informed consent prior to study procedures and understands and agrees to adhere to planned study procedures through Day 28

Exclusion

- Asymptomatic and had symptoms of acute infection that have resolved (for >24 hours)
- Asymptomatic and has received a vaccination for COVID-19 (≥1 dose)
- Undergoing evaluation for hospital admission for medical management (not for public health purposes)
- Evidence of pneumonia and/or hypoxia due to COVID-19
- Prior receipt of immunoglobulin product or passive immune therapy for SARS-CoV-2 in the past 90 days
- Known thrombotic diathesis, recent thrombosis, or procoagulant conditions or disorders
- History of hypersensitivity to blood, plasma or IVIG excipients
- Known IgA deficiency or anti-IgA antibodies
- Medical condition for which receipt of 300 mL volume of IV fluid for study treatment may pose risk (for example, decompensated congestive heart failure)
- In the opinion of the investigator, any condition for which participation would not be in the best interest of the participant or that could prevent or confound protocol assessments

OTAC INSIGHT012

PROTOCOL ASSESSMENTS

Days 1-7
Home self-assessment of SpO_2, temperature, and alert symptoms

"Low-touch" protocol eases study burden. All visits except infusion and Day 7 visit may be virtual if participant is doing well. Day 7 labs can be collected at home.

Day 0
Randomization
Study Infusion
Pre-infusion medical evaluation and specimen collection (nasal, saliva, plasma, serum, and clinical labs)

Day 1
Clinical status (symptom) and hospitalization

Day 4
Clinical status (symptom) and hospitalization

Day 7
Clinical status (symptom) and hospitalization
Specimen collection (nasal, saliva, plasma, serum and clinical labs)

Day 14 (+/-2)
Clinical and hospitalization status

Day 28 (+/-2)
Clinical and hospitalization status

Primary endpoint assessed at Day 7: Clinical status on an ordinal scale of COVID-19 disease severity.

Day 0 is defined as the **day of randomization**
Baseline clinical assessment will occur on day 0 and up to just prior to infusion of study drug

10

OTAC INSIGHT012

Supplementary Figure 17. FAQ: Hyperimmune IVIG in OTAC. Developed to provide information about the study product, anti-coronavirus hyperimmune intravenous globulin (hIVIG), for study staff, it contained information about the rationale for studying hIVIG, its potential activity against SARS-CoV-2 variants, potential advantages over convalescent plasma and monoclonal antibodies, concomitant use of monoclonal antibodies, and relevance to different populations globally.

insight

OTAC INSIGHT012

INSIGHT 012: Outpatient Treatment with Anti-Coronavirus Immunoglobulin (OTAC)

FAQ: Hyperimmune IVIG in OTAC

1. What is the potential value of hIVIG in early COVID-19 disease among outpatients?

Passive immunotherapy has demonstrated benefit in early COVID-19 disease and/or in patients at risk of disease progression. Multiple randomized controlled trials (RCT), evaluating monoclonal antibodies (mAbs) in adults with early disease, have shown that passive immunotherapy, especially mAb combinations, is effective in preventing disease progression and the need for hospitalization. This has led to an FDA Emergency Use Authorization (EUA) of several mAbs, including bamlanivimab + etesevimab, and REGEN-COV (casirivimab + imdevimab). Additionally, while outcomes from CP trials have been mixed, positive outcomes have been observed when high titer plasma is infused early in the course of infection rather than in the hospitalized setting. Hyperimmune IVIG may have advantages over CP and monoclonal antibodies, as explained in the answers to subsequent questions. The hypothesis is that by providing a broad array of high potency neutralizing antibodies to outpatients early in the disease, before the patient has had a chance to produce endogenous antibody, we can lower viral burden and prevent progression to severe disease. These potent antibodies are expected to block the virus, including newer viral variants, from entering cells, blocking the virus from reproducing itself to high levels. *These observations suggest the potential benefit of hIVIG for patients with early disease with risk factors for disease progression, such as those under study in OTAC.*

2. What are the advantages of hIVIG over CP?

Hyperimmune IVIG is a standardized and concentrated product derived from multiple donors providing a broader range of passive immunity, targeting multiple antigenic epitopes, and delivering consistent potency compared to "convalescent plasma," which is typically obtained from an individual COVID-19 patient (Figure 1).

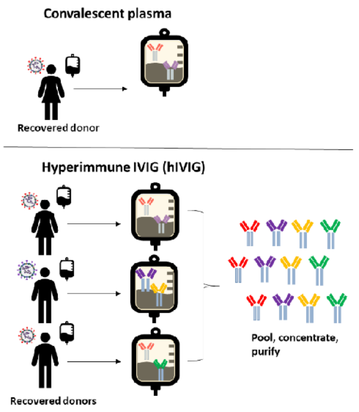

Figure 1

3. If monoclonal antibodies have been approved for emergency use in COVID-19 outpatients, why should we study hIVIG?

SARS-CoV-2 mutations have rendered certain monoclonal antibodies less effective. Hyperimmune immunoglobulin (hIVIG) can exert *broad activity against SARS-CoV-2 variants of concern (VOC)* as shown by recent in vitro data.

Hyperimmune IVIG is a polyclonal product derived from multiple donors. This provides antibodies active against a spectrum of viral genotypes. This contrasts with monoclonal antibodies (mAbs), which bind specifically to a single viral protein.

In the case of mAbs, a single mutation can negatively impact the efficacy of the product. Conversely, hIVIG, targeting many epitopes, is expected to maintain binding and antiviral activity (Figure 2). Laboratory testing by the FDA of hIVIG products from the two companies providing hIVIG for OTAC demonstrated maintenance of activity against viruses engineered to express SARS-CoV-2 protein that bears the same mutations and/or deletions as key SARS-CoV-2 variants of concern (Figure 3), such as those harboring the E484K mutation, as well as other mutations that may affect potency of vaccine-induced antibody, CP, or certain monoclonal antibodies.

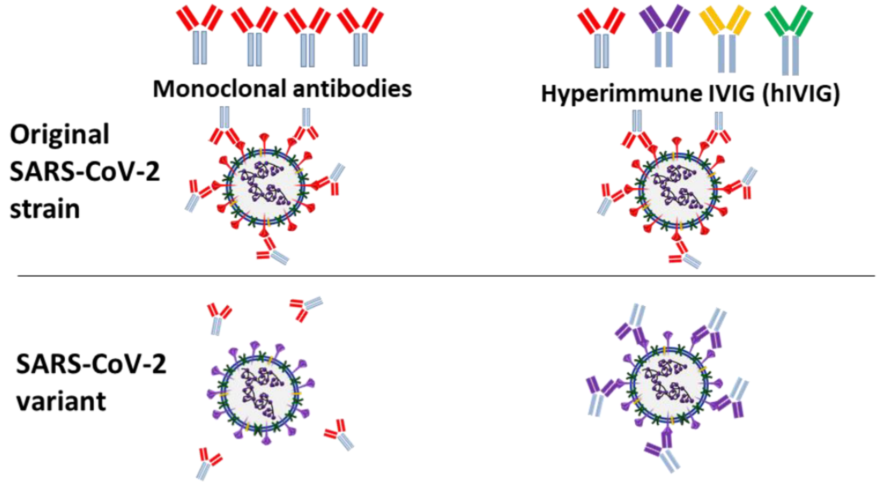

Figure 2: Polyclonal hIVIG is expected to maintain activity against viral variants because of the breadth of the antibody repertoire compared with monoclonal antibody.

In the next figure (Figure 3), in vitro data show that "pseudovirus" bearing mutations associated with different variants of concern may exhibit significantly decreased susceptibility to a number of monoclonal antibodies but not hIVIG. Green signifies no meaningful reduction in neutralizing titer.

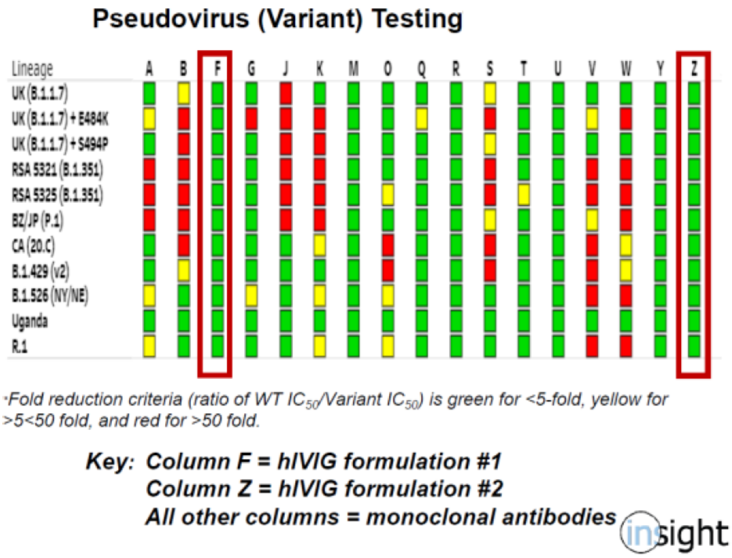

Figure 3

4. ***Can I still use a monoclonal antibody in a patient enrolled in this study if I feel that is in the patient's best interest?***

OTAC has been deliberately designed to allow for the concomitant use of a monoclonal antibody or monoclonal antibody combination if that is deemed to be in the patient's best interests and consistent with local standard of care (SOC) and treatment guidelines. Ideally, we will be able to show that hIVIG is superior to placebo and, among those patients who receive a monoclonal as part of SOC, that hIVIG + SOC containing a monoclonal is superior to SOC with a monoclonal alone (i.e., without hIVIG). This study design will provide important data on hIVIG given in addition to standard of care of various types, and therefore increase generalizability of results.

5. ***If I have a vaccinated patient who develops symptomatic COVID-19, can I still consider them for this study? They should already have antibody on board, so is there any reason for studying hIVIG in these patients?***

As mentioned above, SARS-CoV-2 variants may escape an individual's immune response to a vaccine, but may be less likely show resistance to hIVIG with high titer polyclonal antibodies. As such, there may be benefit in treating these vaccinated patients with "breakthrough" infection. Vaccinated subjects with symptoms of COVID-19 are eligible to enroll in the study. In addition, this study is deliberately enrolling patients (individuals aged >55 and those who are immunocompromised or immunosuppressed) who may have an attenuated immune response to a vaccine. In such a case, exogenous antibody (passively supplied hIVIG) may be helpful. Only data from the study will answer the question, which needs to address this very pragmatic concern now that an increasing proportion of the population is being

vaccinated. The 012 OTAC trial of hIVIG coincides with vaccination roll out, and we hope the trial will be able to determine what the impact of hIVIG is in addition to standard of care among participants who are infected with SARS-CoV-2 despite being vaccinated.

6. Will the results of this study be relevant to the patients we treat and those in other parts of the world?

This is an international trial in 6 continents with sites in the US, Mexico, UK, Greece, Uganda, Denmark, Spain, Indonesia, India, and, possibly other countries. These sites cut across a spectrum of ethnicities, racial backgrounds, and standards of care, and the study has been proactively designed to account for differences in SOC during statistical analysis. Global reach also helps us to collect adequate data for multiple variants of concern, which vary in frequency geographically. Thus, the study results will be generalizable and will allow subset analyses.

Supplementary Figure 18. Dear Colleague Letter. This was created in a one-page format and included medical language, study name, eligible population, study aims, information about the study drug (hIVIG), schedule of study participation activities, and customizable site staff contact information.

OTAC INSIGHT012

OUTPATIENT TREATMENT with ANTI-CORONAVIRUS IMMUNOGLOBULIN

insight

Dear Colleague,

The Outpatient Treatment with Anti-Coronavirus Immunoglobulin (OTAC) study is an outpatient clinical research study sponsored by the National Institutes of Health (NIH). OTAC is currently enrolling people who have tested positive for SARS-CoV-2 within the past 5 days, and who are either 55 years of age or older or who are 18 years or older and immunocompromised (or on immunosuppressive therapy). Eligible participants must be either asymptomatic or have experienced an onset of symptoms within the past 5 days.

The OTAC study compares the safety and efficacy of a single infusion of hyperimmune intravenous immune globulin (hIVIG) versus placebo among adults with recently diagnosed SARS-CoV-2 infection who do not require hospitalization. Passive immune therapy with antibodies targeting SARS-CoV-2 is permitted, if recommended as part of local standard of care.

Eligible consenting participants will be randomized and receive either placebo or the study drug, hIVIG. All patients will also receive standard of care. Following screening and randomization, participants will receive study drug or placebo and then be followed in the study for approximately 28 days, during which 4 additional study visits will take place in the study clinic, at home, or over the phone.

Qualifying participants must:

- Be either 55 years of age or older OR ≥ 18 years of age and immunocompromised/suppressed.
- Have a positive test for SARS-CoV-2 within the last 5 days. Tests may include nucleic acid amplification test (NAAT) or any protocol-approved rapid test.
- If symptomatic, symptom onset must be within the last 5 days.
- If asymptomatically infected, must not have previously received any vaccine doses for COVID-19.
- Not be undergoing evaluation for admission to hospital for medical management.
- Not have evidence of pneumonia and/or hypoxia due to COVID-19.
- Agree not to participate in another treatment trial through Day 7, unless disease progresses significantly.
- Provide written informed consent prior to study procedures and understand/agree to adhere to planned study procedures through Day 28.
- A suitable candidate to participate in the study in the opinion of the investigator.

I invite you to speak with any interested patients who you think may be eligible for this clinical research study. Please send the OTAC referral form to our research fax at _______. If you have any questions and would like to discuss further, you can contact our research nurse, _________ at _________ or myself at __________. Your assistance with increasing awareness of this study is appreciated.

Sincerely,

Supplementary Figure 19. Site Survey Questionnaire. This is the survey instrument that was used to evaluate the usefulness of the study-specific materials by the study site personnel.

Survey of ICCs/Sites About Use of GPP/Study Promotion Materials

Thank you for your participation in the INSIGHT/DCR COVID-19 studies. A number of materials were produced to assist with recruitment, consent, and study conduct. We welcome your feedback so that we can understand which tools and materials were most helpful and how you used them, learn about any successes and challenges you had with using them, and determine how we can improve the tools and materials we provide for future INSIGHT/DCR COVID-19 studies like STRIVE.

INSIGHT/DCR Good Participatory Practices (GPP) Team

1. Does your site use Advarra as a central Institutional Review Board (IRB) for approval of participant-facing materials?
    - Yes
    - No

   Comments:
2. Does your site require additional/separate IRB/Ethics Committee (EC) approval for participant-facing materials?
    - Yes
    - No

   Comments:
3. Please provide information that we should have to ensure your site can use the materials produced. This may include characteristics of the materials that are likely to be rejected by your local/national IRB/ EC; geographic, cultural, and other features that may make the materials more or less appealing or appropriate for your populations; the time by which you require Flipbooks or other materials for submission to your IRB/EC; and unsuitable materials that your site cannot use. Please be specific so that we can understand adjustments we need to make.
   Comments:

4. Which languages did your site use for participant-facing materials (select all that apply)?
- Arabic
- Bahasa
- Chinese, Simplified
- Chinese, Traditional
- Danish
- English
- French
- French (West Africa)
- Greek
- Haitian Creole
- Hebrew
- Hindi
- Hmong
- Khmer
- Korean
- Lao
- Luganda
- Oromo
- Portuguese (Brazil)
- Portuguese (Portugal)
- Punjabi
- Russian
- Somali
- Spanish (Latin America)
- Spanish (Spain)
- Thai
- Tongan
- Vietnamese
- Other
  Please specify

5. My site enrolled participants in the Outpatient Treatment with Anti-Coronavirus Immunoglobulin (**OTAC**) Study (An International Multicenter, Randomized, Double-Blind, Placebo-Controlled Trial of the Safety and Efficacy of Anti-Coronavirus Hyperimmune Intravenous Immunglobulin for the Treatment of Adult Outpatients in Early Stages of COVID-19 (INSIGHT 012))
    - Yes
    - No

   Comments:

   [If Yes, go to questions about OTAC materials. If No, skip to questions about TICO]

6. Please provide information about the purposes for which your site used the OTAC tools and materials. Check all that apply for each of the tools and materials your site used. Check "Other" if the tool or material was (also) used for a purpose not specified.

**OTAC**	Inform potential participants, families, or caregivers	Assist with participant consent	Inform or educate site staff	Solicit referrals from health care providers in your institution	Solicit referrals from health care providers outside your institution	Other
Study Overview Slide Presentation						
2-page Quick Reference Guide						
Dear Colleague Letter						
FAQ: hIVIG						
Study Overview Video						
Flipbook						
Audio Flipbook						
Video Flipbook						
Recruitment Flyers						
CombatCOVID Webpages						
Electronic Tablet						

7. For each tool or material that you indicated "Other" in the previous question, please specify below how else your site used that tool or material.
   Study Overview Slide Presentation
   2-page Quick Reference Guide
   Dear Colleague Letter
   FAQ: hIVIG
   Study Overview Video
   Flipbook
   Audio Flipbook

Video Flipbook
Recruitment Flyers
CombatCOVID Webpages
Electronic Tablet

8. How helpful were the OTAC tools and materials which your site used? Please rate them using the following scale:
   * Not useful   **Minimally useful   *** Somewhat useful   ****Very useful
   *****Extremely useful
   Please rank the **three** tools or materials your site found most useful for OTAC. Select 1 for the most useful, 2 for the next most useful, and 3 for the third most useful.

**OTAC**	How helpful was each of the tools or materials your site used? Please use the drop-down menu to rate.	Please rank the **top 3** most useful tools or materials your site used.
Study Overview Slide Presentation		
2-page Quick Reference Guide		
Dear Colleague Letter		
FAQ: hIVIG		
Study Overview Video		
Flipbook		
Audio Flipbook		
Video Flipbook		
Recruitment Flyers		
CombatCOVID Webpages		
Electronic Tablet		

9. How helpful was the OTAC Participant Brochure for Temperature and Oximetry Monitoring?
   * Not useful   **Minimally useful   *** Somewhat useful   ****Very useful
   *****Extremely useful   Not used
   Comments:
10. What difficulties or challenges did you/your site face with using the OTAC tools and materials?
    Comments:
11. What improvements would you suggest for the OTAC tools and materials?
    Comments:
12. What additional tools and materials would be helpful for OTAC?
    Comments:
13. My site enrolled participants in the Therapeutics for Inpatients with COVID-19 (**TICO**) Study (A Multicenter, Adaptive, Randomized, Blinded Controlled Trial of the Safety and Efficacy of Investigational Therapeutics for Hospitalized Patients with COVID-19 (INSIGHT 014))
    - Yes

- No

Comments:

[If Yes, go to questions about TICO materials. If No, go to questions about TESICO.]

14. Please provide information about the purposes for which your site used the TICO tools and materials. Check all that apply for each of the tools and materials your site used. Check "Other" if the tool or material was (also) used for a purpose not specified.

**TICO**	Inform potential participants, families, or caregivers	Assist with participant consent	Inform or educate site staff	Solicit referrals from health care providers in your institution	Solicit referrals from health care providers outside your institution	Other
Study Overview Video						
Flipbook						
Audio Flipbook						
Video Flipbook						
D28 VATICO Recruitment Letter (Long)						
D28 VATICO Recruitment Letter (Short)						
D28 VATICO Recruitment Letter (Pictures)						
CombatCOVID Webpages						
Electronic Tablet						

15. For each tool or material that you indicated "Other" in the previous question, please specify below how else your site used that tool or material.

Study Overview Video

Flipbook

Audio Flipbook

Video Flipbook

D28 VATICO Recruitment Letter (Long)

D28 VATICO Recruitment Letter (Short)

D28 VATICO Recruitment Letter (Pictures)

CombatCOVID Webpages

Electronic Tablet

16. How helpful were the TICO tools and materials which your site used? Please rate them using the following scale:

* Not useful **Minimally useful *** Somewhat useful ****Very useful *****Extremely useful

Please rank the **three** tools or materials your site found most useful for TICO. Select 1 for the most useful, 2 for the next most useful, and 3 for the third most useful.

**TICO**	How helpful was each of the tools or materials your site used? Please use the drop-down menu to rate.	Please rank the **top 3** most useful tools or materials your site used.
Study Overview Video		
Flipbook		
Audio Flipbook		
Video Flipbook		
D28 VATICO Recruitment Letter (Long)		
D28 VATICO Recruitment Letter (Short)		
D28 VATICO Recruitment Letter (Pictures)		
CombatCOVID Webpages		
Electronic Tablet		

17. What difficulties or challenges did you/your site face with using the TICO tools and materials?
Comments:

18. What improvements would you suggest for the TICO tools and materials?
Comments:

19. What additional tools or materials would have been helpful for TICO?
Comments:

20. My site enrolled participants in the Vaccination for Recovered Inpatients with COVID-19 (**VATICO**) Substudy (SARS-CoV-2 vaccination strategies in previously hospitalized and recovered COVID-19 patients (INSIGHT 016))
    - Yes
    - No

Comments:

[If Yes, go to questions about VATICO materials. If No, skip to questions about TESICO.]

21. Please provide information about the purposes for which your site used the VATICO tools and materials. Check all that apply for each of the tools and materials your site used. Check “Other” if the tool or material was (also) used for a purpose not specified.

VATICO	Inform potential participants, families, or caregivers	Assist with participant consent	Inform or educate site staff	Solicit referrals from health care providers in your institution	Solicit referrals from health care providers outside your institution	Other
Participant Letter for Vaccination Centers						
Proof of Recovery and Vaccination Card						
Participant Letter and Study Information						
Flipbook						
Audio Flipbook						
Video Flipbook						

22. For each tool or material that you indicated "Other" in the previous question, please specify below how else your site used that tool or material.
    Participant Letter for Vaccination Centers
    Proof of Recovery and Vaccination Card
    Participant Letter and Study Information
    Flipbook
    Audio Flipbook
    Video Flipbook

23. How helpful were the VATICO tools and materials which your site used? Please rate them using the following scale:
    * Not useful **Minimally useful *** Somewhat useful ****Very useful
    *****Extremely useful
    Please rank the **three** tools or materials your site found most useful for VATICO. Select 1 for the most useful, 2 for the next most useful, and 3 for the third most useful.

VATICO	How helpful was each of the tools or materials your site used? Use the drop-down menu to rate.	Please rank the **top 3** most useful tools or materials your site used.
Participant Letter for Vaccination Centers		
Proof of Recovery and Vaccination Card		
Participant Letter and Study Information		
Flipbook		
Audio Flipbook		
Video Flipbook		

24. What difficulties or challenges did you/your site face with using the VATICO materials?
    Comments:

25. What improvements would you suggest for the VATICO materials?
Comments:

26. What additional tools and materials would have been helpful for VATICO?
Comments:

27. My site enrolled participants in the Therapeutics for Severely Ill Inpatients with COVID-19 (**TESICO**) Study (A Multicenter, Adaptive, Randomized, Blinded Controlled Trial of the Safety and Efficacy of Investigational Therapeutics for Hospitalized Patients with Acute Respiratory Distress Syndrome Associated with COVID-19 (INSIGHT 015))
    - Yes
    - No

    Comments:

[If Yes, go to questions about TESICO materials. If No, skip to final questions.]

28. Please provide information about the purposes for which your site used the TESICO tools and materials. Check all that apply for each of the tools and materials your site used. Check "Other" if the tool or material was (also) used for a purpose not specified.

**TESICO**	Inform potential participants, families, or caregivers	Assist with participant consent	Inform or educate site staff	Solicit referrals from health care providers in your institution	Solicit referrals from health care providers outside your institution	Other
Study Overview Video						
Poster						
Flipbook						
Audio Flipbook						
Video Flipbook						
CombatCOVID Webpages						
Electronic Tablet						

29. For each tool or material that you indicated "Other" in the previous question, please specify below how else your site used that tool or material.
    Study Overview Video
    Poster
    Flipbook
    Audio Flipbook
    Video Flipbook
    CombatCOVID Webpages
    Electronic Tablet

30. How helpful were the TESICO tools and materials which your site used? Please rate them using the following scale:
* Not useful **Minimally useful *** Somewhat useful ****Very useful
*****Extremely useful
Please rank the **three** tools or materials your site found most useful for TESICO. Select 1 for the most useful, 2 for the next most useful, and 3 for the third most useful.

**TESICO**	How helpful was each of the tools or materials your site used? Use the drop-down menu to rate.	Please rank the **top 3** most useful tools or materials your site used.
Study Overview Video		
Poster		
Flipbook		
Audio Flipbook		
Video Flipbook		
CombatCOVID Webpages		
Electronic Tablet		

31. What difficulties or challenges did you/your site face with using the TESICO tools and materials?
Comments:

32. What improvements would you suggest for the TESICO tools and materials?
Comments:

33. What additional tools and materials would be helpful for TESICO?
Comments:

34. Other than low COVID-19 cases in your community, what challenges to recruitment, consent, or study conduct did your site face that were not addressed by the tools and materials?
Comments:

35. Please provide any additional comments or suggestions.
Comments or suggestions:

THANK YOU FOR TAKING THE TIME TO COMPLETE THIS SURVEY!

Your feedback is very much appreciated.
